# Amide nitrogen pyramidalization changes lactam amide spinning

**DOI:** 10.1038/s41467-018-08249-9

**Published:** 2019-01-28

**Authors:** Yuko Otani, Xin Liu, Hisashi Ohno, Siyuan Wang, Luhan Zhai, Aoze Su, Masatoshi Kawahata, Kentaro Yamaguchi, Tomohiko Ohwada

**Affiliations:** 1 Graduate School of Pharmaceutical Sciences, University of Tokyo, 7-3-1 Hongo, Bunkyo-ku, Tokyo 113-0033 Japan; 2 Faculty of Pharmaceutical Sciences at Kagawa Campus, Tokushima Bunri University, 1314-1 Shido, Sanuki, Kagawa 769-2193 Japan

## Abstract

Although *cis*-*trans* lactam amide rotation is fundamentally important, it has been little studied, except for a report on peptide-based lactams. Here, we find a consistent relationship between the lactam amide *cis/trans* ratios and the rotation rates between the *trans* and *cis* lactam amides upon the lactam chain length of the stapling side-chain of two 7-azabicyclo[2.2.1]heptane bicyclic units, linked through a non-planar amide bond. That is, as the chain length increased, the rotational rate of *trans* to *cis* lactam amide was decreased, and consequently the *trans* ratio was increased. This chain length-dependency of the lactam amide isomerization and our simulation studies support the idea that the present lactam amides can spin through 360 degrees as in open-chain amides, due to the occurrence of nitrogen pyramidalization. The tilting direction of the pyramidal amide nitrogen atom of the bicyclic systems is synchronized with the direction of the semicircle-rotation of the amide.

## Abstract (web summary)

Cis-trans lactam amide rotation is a fundamental process and its understanding might aid molecular design. Here, the authors report the synthesis and study of bicyclic lactams which undergo spin through 360 degrees as in open-chain amides, due to the occurrence of nitrogen pyramidalization.

## Introduction

**Fig. 1:**
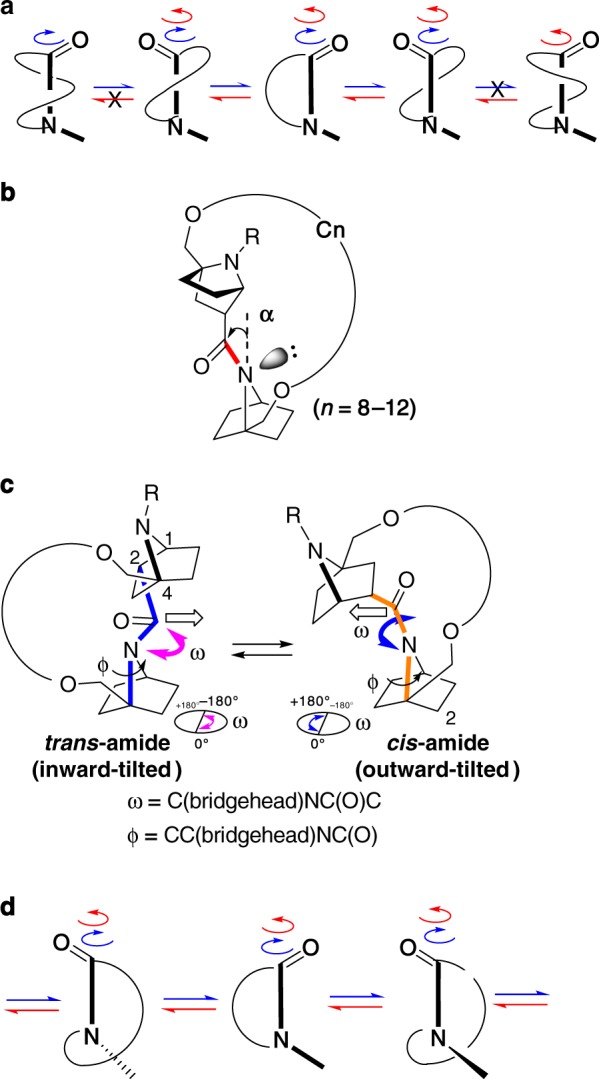
Lactam amide rotations. **a** Hindered rotation of coiled lactam amides. **b** Nitrogen pyramidalization of tertiary amide lactams of 7-azabicyclo[2.2.1]heptane. **c** Equilibrium of *trans* and *cis* amides in the bicyclic N-pyramidal lactam. **d** Spinning of N-pyramidal lactam through 360 degrees

Rotation of amides is of fundamental importance^1,2^, being involved in a wide range of structural phenomena ranging from protein folding^3–6^ to the conformational multiplicity of cyclic peptides^7–11^. In the case of cyclic amides (lactams), rotation of the amide is influenced by the intervening chain length and nature of the rotatable bonds. If a lactam amide bond could spin through 360 degrees as in open-chain amides, a simple correlation between the ratio of amide *trans/cis* isomers and the rotational rate(s) between *trans* and *cis* amide conformers should be observed, e.g., as the rotation rate from *trans* to *cis* isomers increases, the ratio of *cis* isomer increases. While there have been few studies of the rotation rate of lactam amides, the rotation rate of α−proline amide upon intramolecular disulfide bond formation between two flanking cysteine (Cys) residues was reported to show an inconsistent dependency of *cis*-*trans* isomer ratios and rates of *cis*-*trans* interconversion on chain length. This is probably because spinning of the amide bond is discontinuous and also because the energy barrier of amide rotation is relatively high (~20 kcal/mol)^12–14^. Therefore, this observation suggested that the rotation of lactam amide is restricted^15–17^, and as a general trend, the amide bond in lactams cannot spin through 360 degrees, probably due to ring coiling (Fig. 1a). For example, a simple N-methyl tertiary amide lactam, a medium-ring-sized 1-methylazacycloundecan-2-one (11-membered ring) (**S-A**, Supplementary Fig. 1) and a simple secondary amide lactam, a large-ring-sized 1-azacyclononadecan-2-one (19-membered ring) (**S-B**, Supplementary Fig. 2), exhibited discontinuous spinning of the lactam amide bond, not through 360° (see also Supplementary Fig. 3).

On the other hand, while non-planar amides have been studied intensively^18–26^, their characteristic structural features and the consequent reduction of amide rotational barriers have been little utilized in molecular architecture, mainly because of the instability of the amide bond under conventional transformation conditions. However, examination of tertiary amides of 7-azabicyclo[2.2.1]heptane has shown that this bicyclic system induces nitrogen pyramidalization (Fig. 1b)^27^, and these amides are stable enough to be subjected to further transformations^28^.

The rotational barriers of amide *cis-trans* isomerization of nitrogen-pyramidal amides are reduced in solution (~17 kcal/mol), indicating more facile amide rotation as compared with conventional planar amides (>20 kcal/mol). Inversion of the amide nitrogen atom is a low-energy process (calculated activation energy: less than 3 kcal/mol), and nitrogen pyramidalization is also a facile process^27,29^. Further, derivatives bearing a carboxylic acid functionality at the C_2_-position can be regarded as conformation-constrained β-proline surrogates. In these bicyclic β-proline surrogate 7-azabicyclo[2.2.1]heptane-2-carboxylic acids (Fig. 1c), it is shown that the presence of the bridgehead substituent at the C_4_-position completely biases the amide *cis*-*trans* equilibrium to one side (*trans* amide), irrespective of solvent^30–32^. This led us to consider that side-chain stapling of the bicyclic amide dimer would affect the thermodynamics and kinetics of non-planar amide lactam rotation in a very definite manner (Fig. 1c). Therefore, we expected that this bicyclic system would provide a suitable scaffold to examine the stapling length dependency of lactam amide ratio and rotation (see below). Here, we show a simple relationship; i.e., a consistent increase of *trans* ratio in accordance with a consistent deceleration of amide *trans* to *cis* rotation, together with concomitant acceleration of amide *cis* to *trans* rotation, as the side-chain staple length is increased in bicyclic dimers, eventually affording a very large dynamic range of variation in amide *cis*-*trans* ratios, extending to complete inversion of the amide *cis*-*trans* equilibrium, depending upon the side-chain staple length (Fig. 1d).

## Results

### Analysis of amide *cis-trans* isomerization

**Fig. 2:**
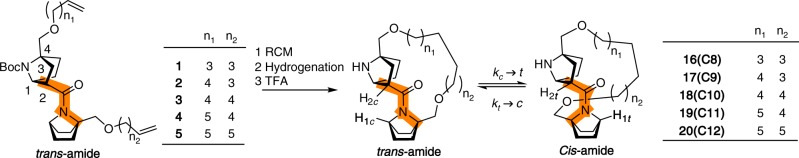
Lactams containing two 7-azabicyclo[2.2.1]heptane bicyclic units. Stapling of model bridgehead-substituted bicyclic dimers with various lengths of alkyl side chain by ring-closing metathesis (RCM) and hydrogenation

We used ring-closing metathesis (RCM)^33,34^ to staple model bridgehead-substituted bicyclic dimers with various lengths of alkyl side chain (Fig. 2). Cyclic dimer lactams (**16–20**) with linkers of different lengths were synthesized as racemic mixtures (for details, see Supplementary Methods). The RCM reactions of **1–5**, which are Boc-protected open-chain model dimers with alkenyl side chains of various lengths, were performed using the Grubbs I catalyst to afford stapled olefin products **6–10** as mixtures of *E*-olefins and *Z*-olefins in good yields (Supplementary Fig. 18). Hydrogenation of the alkenes gave N-Boc saturated compounds **11–15**, respectively (Supplementary Fig. 18). Some of the structures were confirmed by means of X-ray crystallographic analyses (see below).

The Boc group was removed by treatment of **11–15** with TFA, followed by base treatment to give the dimer free amines (**16(C8)**, **17(C9)**, **18(C10)**, **19(C11)**, and **20(C12)**) (for chemical structures, see Fig. 2), where the number C*n* indicates the number (*n*) of carbons between two ether oxygen atoms of the linker. The amide *cis/trans* ratio of the dimer lactams (**16(C8)**-**20(C12)**) was estimated from the NMR spectra in CD_2_Cl_2_. We found that the ratio changed dramatically depending upon the linker length (Table 1). The open-chain amide analogue **1–5** (which can be regarded as equivalent to a cyclic molecule with an infinite linker length) takes exclusively the *trans*-amide structure (Table 1). The ratio of *cis*-amide in the lactam analogs is increased as the linker length is decreased and the ratio changes from predominantly *trans* (**20(C12)**, 94% *trans*, *K*_c/t_ = 0.06) to almost exclusively *cis* (**16***(Z)***-NH(C8)(Z-olefin)**, 99.9% *cis*, *K*_c/t_ > 999) (Table 1). X-Ray crystallographic analysis of the synthetic intermediate unsaturated derivatives provided supporting evidence for the structural assignments (see below, Supplementary Figs. 14–16). As the chain length decreased, the energy difference between *cis* and *trans* lactam amides changed (*ΔΔG*_*cis-trans*_ *=* *ΔG*_*cis*_*–ΔG*_*trans*_, see Table 1): *ΔΔG*_*cis-trans*_ (in CD_2_Cl_2_); **19(C11)**: + 1.08 kcal/mol (*trans* more stable than *cis*); **18(C10)**: + 0.59 kcal/mol (*trans* more stable than *cis*); **17(C9)**: −1.08 kcal/mol (*cis* more stable than *trans*) (Table 1): *cis* structure became more stable than *trans* structure. Also, there is a clear difference in the preference for *trans*- or *cis*-amide between lactams **17(C9)** and **18(C10)**. We found that **17(C9)** and **18(C10)** exhibit similar *trans/cis* ratios in CD_2_Cl_2_ (**17**: 14/86; **18**: 73/27) and protic CD_3_OD (**17**: 24/76; **18**: 77/23), showing small dependency of solvent polarity on *cis/trans* isomer distribution.

**Table 1:** Equilibrium constants of amide cis-trans isomerization at −50 °C

	Linker length	*trans/cis* (%)^a^	*K*_c/t_^b^	ΔΔ*G*_cis-trans_ (25 °C)
**6**(Z)-NH (CD_2_Cl_2_)	C8	<0.1 />99.9^c^	>999	< −4.10
**6**(E)-NH (CD_2_Cl_2_)	C8	16/84	5.25	−0.98
**16** (CD_2_Cl_2_)	C8	8/92	11.5	−1.45
**17** (CD_2_Cl_2_)	C9	14/86	6.14	−1.08
**18** (CD_2_Cl_2_)	C10	73/27	0.37	+0.59
**19** (CD_2_Cl_2_)^d^	C11	86/14	0.16	+1.08
**20** (CD_2_Cl_2_)	C12	94/6	0.06	+1.63
**1** (CD_2_Cl_2_)	Open	>99.9/<0.1	<0.001	>+ 4.10
**16** (CD_3_OD)	C8	16/84	5.25	−0.98
**17** (CD_3_OD)	C9	24/76	3.17	−0.68
**18** (CD_3_OD)	C10	77/23	0.30	+0.72

^a^Errors in the ratios of *cis* and *trans* are within ± 1%

^b^*K*_c/t_ = [*cis*]/[*trans*]

^c^The *trans* rotamer was below the detection limit

^d^Measured at −47.8 °C

### Chain-length-dependent amide rotation kinetics

**Fig. 3:**
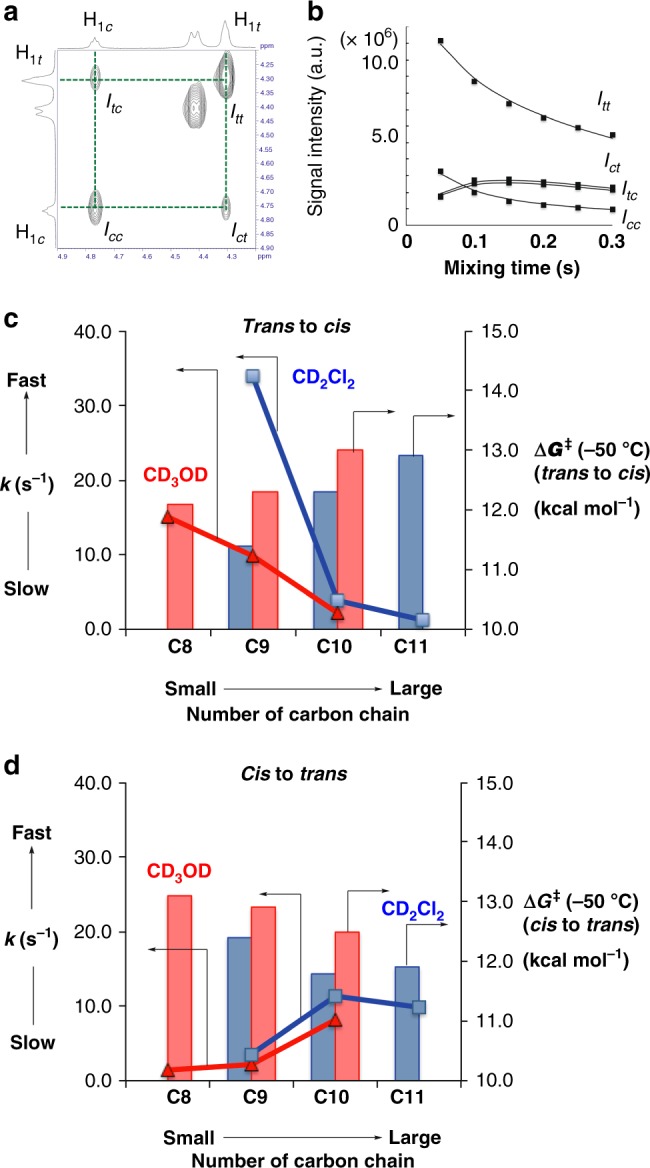
Kinetic studies of lactam amide rotations. **a** EXSY spectrum of **18(C10)** in CD_2_Cl_2_ at −50.3 °C with a mixing time of 50 ms. **b** Plots of the signal intensities of **18(C10)** versus the mixing time (−50.3 °C). Solid lines indicate the best fit of the data to the theoretical equation. **c** Dependency of isomerization rate of *trans* to *cis* amide isomers on the staple length. **d** Dependency of isomerization rate of *cis* to *trans* amide isomers on the number of chain carbon atoms. Blue square and blue line: the rotation rate in CD_2_Cl_2_ at −50.3 °C; red triangle and red line: the rotation rate in CD_3_OD at −42.6 °C. Blue bar: the rotation barrier (Δ*G*^‡^_(−50.0 °C)_) in CD_2_Cl_2_, red bar: Δ*G*^‡^_(−50.0 °C)_ in CD_3_OD

The rotational kinetics of the amide bond of the bicyclic dimer with different linker lengths (**16(C8)**, **17(C9)**, **18(C10)** and **19(C11)**) was estimated by means of exchange spectroscopy (EXSY)^35–39^ (Fig. 3a, b). We measured the kinetics of **17(C9)**, **18(C10)**, and **19(C11)** in CD_2_Cl_2_, and **16(C8)**, **17(C9)**, and **18(C10)** in CD_3_OD (Fig. 3c, d). The EXSY spectra were recorded with six mixing times (*T*_m_) of 20, 40, 60, 100, 200, and 300 ms at 215.4 K, 217.9 K, 220.4 K, 222.9 K, and 225.4 K in CD_2_Cl_2_ and with six mixing times (*T*_mix_) at 222.8 K, 226.8 K, 230.6 K, 234.4 K, and 238.2 K in CD_3_OD. The signal intensities of the four peaks due to one pair of exchanging bridgehead protons were obtained from the peak heights (Fig. 3a): *I*_*tt*_ is the peak intensity of the auto peak of **H**_**1**_ in the *trans* conformation (i.e., H_1*t*_), *I*_*cc*_ is that of the auto peak of **H**_**1**_ in the *cis* conformation (i.e., H_1*c*_), *I*_*tc*_ is that of the cross peak from *trans* to *cis* conformation, and *I*_*ct*_ is that of the cross peak from *cis* to *trans* conformation. (Fig. 2). The assignments were confirmed by the observation of NOE between H_1*c*_ and H_2*c*_ (Supplementary Figs. 4–7). The exchange rate (*k*_*t*→*c*_) from *trans* to *cis* conformation and that (*k*_*c*→*t*_) from *cis* to *trans* conformation at each temperature were estimated by fitting to a modified version of the theoretical equation reported previously (Fig. 3b, Table 2, Supplementary Figs. 8–13)^39,40^. The free energies of rotation (∆*G*_*t*→*c*_^‡^ and ∆*G*_*c*→*t*_^‡^) were obtained from the enthalpy of activation (∆*H*^‡^) and the entropy of activation (∆*S*^‡^), which were derived from the slope and intercept of the Eyring plot of the rate against temperature (Table 2).

**Table 2:** Rate constants and activation parameters for amide cis-trans isomerization

		*trans* → *cis*			
	Solvent	*k*_*t→c*_ (s^−1^*)*	Δ*H*^‡^_*t*→*c*_	Δ*S*^‡^_*t*→*c*_	Δ*G*^‡^_*t*→*c*_^a^
			(kcal·mol^−1^)	(cal·mol^−1^·K^−1^)	(kcal·mol^−1^)
**17(C9)**	CD_2_Cl_2_	33.9^b^	7.9 ± 0.5	−15.6 ± 2.0	11.4 ± 0.1
**18(C10)**	CD_2_Cl_2_	3.9^b^	9.2 ± 0.7	−13.9 ± 3.3	12.3 ± 0.1
**19(C11)**	CD_2_Cl_2_	1.2^b^	10.8 ± 0.8	−9.1 ± 3.6	12.9 ± 0.2
**16(C8)**	CD_3_OD	6.0^b^	11.3 ± 0.3	−3.5 ± 1.1	12.1 ± 0.1
**17(C9)**	CD_3_OD	3.8^b^	12.6 ± 0.6	1.4 ± 2.5	12.3 ± 0.1
**18(C10)**	CD_3_OD	0.9^b^	11.7 ± 0.7	−5.8 ± 3.2	13.0 ± 0.1
**Ac-Ah2c-NHMe** ^c^	CD_3_OD	0.19^d^	17.0 ± 1.0	−2.2 ± 3.1	17.5 ± 1.9

		*cis* → *trans*			
	Solvent	*k*_*c→t*_ (s^−1^*)*	Δ*H*^‡^_*c→t*_	Δ*S*^‡^_*c→t*_	Δ*G*^‡^_*c→t*_^a^
			(kcal·mol^−1^)	(cal·mol^−1^·K^−1^)	(kcal·mol^−1^)
**17(C9)**	CD_2_Cl_2_	3.6^b^	5.3 ± 0.3	−31.9 ± 1.2	12.4 ± 0.1
**18(C10)**	CD_2_Cl_2_	11.4^b^	9.3 ± 0.9	−11.0 ± 4.2	11.8 ± 0.2
**19(C11)**	CD_2_Cl_2_	9.8^b^	12.4 ± 0.9	2.2 ± 3.9	11.9 ± 0.2
**16(C8)**	CD_3_OD	0.6^b^	9.0 ± 0.7	−18.4 ± 2.9	13.1 ± 0.1
**17(C9)**	CD_3_OD	0.8^b^	10.8 ± 0.9	−9.9 ± 3.8	13.0 ± 0.2
**18(C10)**	CD_3_OD	2.8^b^	13.5 ± 0.9	4.7 ± 4.2	12.5 ± 0.1
**Ac-Ah2c-NHMe** ^c^	CD_3_OD	0.17^d^	15.9 ± 0.3	−6.6 ± 0.8	17.4 ± 0.5

Note: Parameters were obtained from unbiased estimates of the standard deviations of least-squares parameters and are reported at the 95% confidence level (ref. ^44^)

^a^Values at −50.0 °C

^b^Values at −50.3 °C

^c^Ref. ^40^

^d^Values at 14.1 °C

The rotation rates in CD_3_OD were apparently smaller than those in CD_2_Cl_2_, suggesting a possible contribution of solvation effects in the rotation process, such as entropy, polarity and viscosity of the solvent (Table 2). The rotation rate *k*_*t*→*c*_ showed a consistent decrease along the sequence from **16(C8)** to **18(C10)** (in CD_3_OD) and from **17(C9)** to **19(C11)** (in CD_2_Cl_2_) (Fig. 3c). On the other hand, the rotation rate from *cis* to *trans* isomer (*k*_*c*→*t*_) showed a small but consistent increase along the sequence from **16(C8)** to **18(C10)** (in CD_3_OD) and from **17(C9)** to **19(C11)** (in CD_2_Cl_2_) (Fig. 3d). Finally, as the number of ring carbon atoms (*C*_*n*_) increases along the sequence from **16(C8)** to **19(C11)**, the magnitude of the rotation rate from *cis* to *trans* isomer (*k*_*c*→*t*_) overwhelms that of the rotation rate from *trans* to *cis* isomer (*k*_*t*→*c*_), which is consistent with the large dynamic range of variation in amide *cis*-*trans* ratios, extending to complete inversion of the *cis*-*trans* equilibrium from *cis* isomer to *trans* isomer.

In fact, these changes in the rotation rate (*k*) are consistent with the changes in the free energy of rotation (∆*G*_*t*→*c*_^‡^ and ∆*G*_*c*→*t*_^‡^): ∆*G*_*t*→*c*_^‡^ (*trans* to *cis*) decreased with reduction in the size of the stapling ring (Fig. 3c and Table 2), while ∆*G*_*c*→*t*_^‡^ (*cis* to *trans*) showed a slight increase (Fig. 3d and Table 2). The obtained rotational barriers of *trans* to *cis* isomer (Δ*G*_*t*→*c*_^‡^) (at 223.2 K) for **17(C9)**-**19(C11)** ranged from 11.4 kcal/mol, 12.3 kcal/mol to 12.9 kcal/mol in CD_2_Cl_2_, and those for **16(C8)**-**18(C10)** ranged from 12.1 kcal/mol, 12.3 kcal/mol to 13.0 kcal/mol in CD_3_OD (Table 2). These values are significantly reduced compared to those of the parent open-chain bicyclic amide (**Ac-Ah2c-NHMe**) (ca. 17 kcal/mol)^40^. As the linker becomes shorter, the enthalpy of activation (Δ*H*^‡^) decreases and entropic ordering is increased. We also saw a similar trend in CD_3_OD.

### Different ring size effects on *cis* and *trans* amide structures

**Fig. 4:**
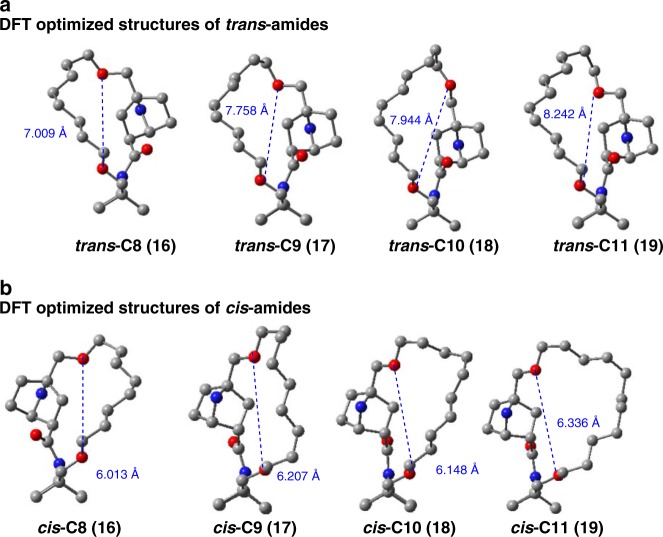
DFT-optimized structures of **16(C8)**-**19(C11)**. **a** DFT-optimized *trans*-isomers of **16(C8)**-**19(C11)**. **b** DFT-optimized *cis*-isomers of **16(C8)**-**19(C11)**. The distance between the ether oxygen atoms in the linker is shown in blue. Brown/gray: carbon atom; blue: nitrogen atom; red: oxygen atom. All hydrogen atoms are omitted for clarity

In order to examine the ring size of the experimentally studied systems, we calculated the energy-minimum structures of lactams **16**–**19** by means of DFT methods (see Supplementary Data and Supplementary Methods). The DFT calculated structures of *trans*-amide structures showed that, as expected, decrease in the stapling chain number (*n*) is accompanied with decrease of the ring size, as exemplified by a large decrease of the distance between the two ether oxygen atoms (*d*_*o-o*_), which is related to the radius of the ring (*trans* forms: **16(C8)**: 7.01 Å; **17(C9)**: 7.76 Å; **18(C10)**: 7.94 Å; **19(C11)**: 8.24 Å) (Fig. 4a). On the other hand *cis*-amide structures showed a small change in the ring size as exemplified by the distance (*d*_*o-o*_) between the two ether oxygen atoms in the ring, and there was a fluctuation at **18(C10)**: the *cis* forms: **16(C8)**: 6.01 Å; **17(C9)**: 6.21 Å; **18(C10)**: 6.15 Å; **19(C11)**: 6.34 Å) (Fig. 4b). From the observed *cis/trans* ratio (Table 1), a short ring linking two adjacent bridgehead groups stabilizes *cis* amide structures, while the *cis* amide was destabilized by increase of chain length.

The dependency of the rotation rate from *trans* to *cis* isomer (*k*_*t*→*c*_) on the chain length can be accounted for by the fact that the increase of the ring size (e.g., elongation of *d*_*O-O*_) led to increase of the *trans* ratio (Table 1), which indicates that increase of the ring size stabilized the *trans* amide structure, probably due to release of the *trans*-lactam ring strain (Fig. 4a). When the initial *trans* structure was stabilized, the activation energies for amide rotation to reach the TS structure may be increased, and therefore amide rotation becomes less feasible, i.e., the rotation rate from *trans* to *cis* isomer (*k*_*t*→*c*_) becomes smaller.

**Fig. 5:**
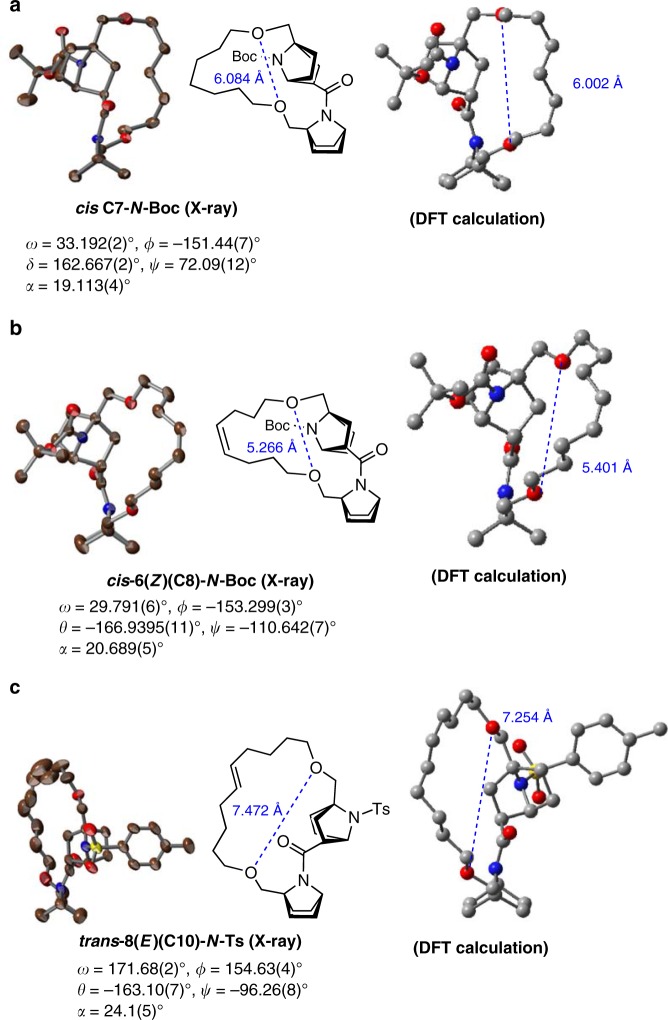
ORTEP drawings (50% probability) of the crystal structures of some derivatives. **a** The crystal structure of *N*-Boc derivative of **C7** in the *cis* amide form. **b** The crystal structure of *N*-Boc derivative of **6**(*Z*) (**C8**) in the *cis*-amide form, and **c** the crystal structure of *N*-tosylated derivative of **8**(*E*) (**Ts-8**(*E*)(**C10**)) in the *trans*-amide form. All three structures were crystallized as racemates in centrosymmetric space groups. DFT-optimized structures are also shown. The distance between the ether oxygen atoms in the linker is shown in blue. Brown/gray: carbon atom; blue: nitrogen atom; red: oxygen atom. All hydrogen atoms are omitted for clarity

For **18(C10)** the change in the rotation rate fluctuated slightly (Fig. 3d, in CDCl_3_), probably because there is a structural inconsistency in *cis* **18(C10)** due to shrinkage of the ring size at **18(C10)** (*d*_*O-O*_: 6.15 Å) as compared with **17(C9)** (*d*_*O-O*_: 6.21 Å), in spite of the increased size of the carbon chain (Fig. 4b). Therefore, although there is a fluctuation, there is a tendency that the rotation rate from *cis* to *trans* isomer (*k*_*c*→*t*_) increases (Fig. 3d) as the stable *cis* isomer become more unstable along the sequence from **16(C8)** to **19(C11)**, judging from the observed decrease in the *cis* ratio (Table 1). This destabilization of the *cis* amide structure upon increase in the ring size is probably due to the increase in strain to maintain the fixed distance between the two ether oxygen atoms. The extension of these two ether linkages is forced to take a parallel direction, as seen in the crystal structures of the *cis* amides, i.e., ***cis*-C7-N-Boc** and ***cis*−6Z-C8-N-Boc** (Fig. 5a, b).

### Tilting direction of the pyramidal nitrogen atom

While the crystal structures (Fig. 5a, b, c) are different from the real system (**16**–**19**) due to the presence of the unsaturation of the alkyl chain and N-substitution (Boc or Ts), it seemed that the amides of the ground states (*trans* and *cis* isomers) were tilted in the opposite direction to the bridgehead substituent; this is particularly apparent in the cases of the **C8** (***cis*−6(Z)(C8)-N-Boc**) and **C10** (***trans*−8(E)(C10)-N-Ts**) analogues (Fig. 5b, c). The DFT-optimized structures, starting from the X-ray structures of ***cis*-C7-N-Boc**, ***cis*−6(Z)-C8-N-Boc**, and ***trans*−8(E)(C10)-N-Ts**, supported this trend (Fig. 5a–c). The tilting of the amide on the same side of the bridgehead substituent induced significant destabilization (calculated as more than 60 kcal/mol). The calculated averaged tilt angles (*α*) were 25.0° and 20.7° for the *trans*-ground state and *cis*-ground state, respectively (Fig. 1b and Fig. 4a, b). These structural features are a consequence of the facile nitrogen pyramidalization of the bicyclic lactam amide.

### Computational evaluation of the lactam amide rotation

DFT calculation was performed to identify features of the rotational process. As a result of an extensive search of amide rotation processes, transition structures for amide rotation of the 2(*S*)-enantiomers of **17(C9)**, **18(C10)**, and **19(C11)** were identified (Supplementary Fig. 53, Supplementary Methods and Supplementary Data); these may represent one of the lowest-energy processes. Both the magnitude of the rotational barrier and the relative stability of *cis-trans* rotamers coincided well with the experimental values. The calculated values of free energy of rotation from *trans* to *cis* (∆*G*^‡^_*t*→*c*_) in CH_2_Cl_2_ at 25 °C were 9.6 kcal/mol, 12.7 kcal/mol and 13.6 kcal/mol in **17(C9)**, **18(C10)**, and **19(C11)**, respectively (Supplementary Table 1). On the other hand, the calculated values of free energy of rotation from *cis* to *trans* (∆*G*^‡^_*c*→*t*_) in methanol at 25 °C were 14.5 kcal/mol, 11.9 kcal/mol and 11.8 kcal/mol in **16(C8)**, **17(C9)**, and **18(C10)**, respectively (Supplementary Table 2). Therefore, the computations reproduced the tendency that the rotational barrier, especially ∆*G*^‡^_*c*→*t*_ (Supplementary Table 2 and Fig. 3d), decreases as the alkyl linker is elongated.

**Fig. 6:**
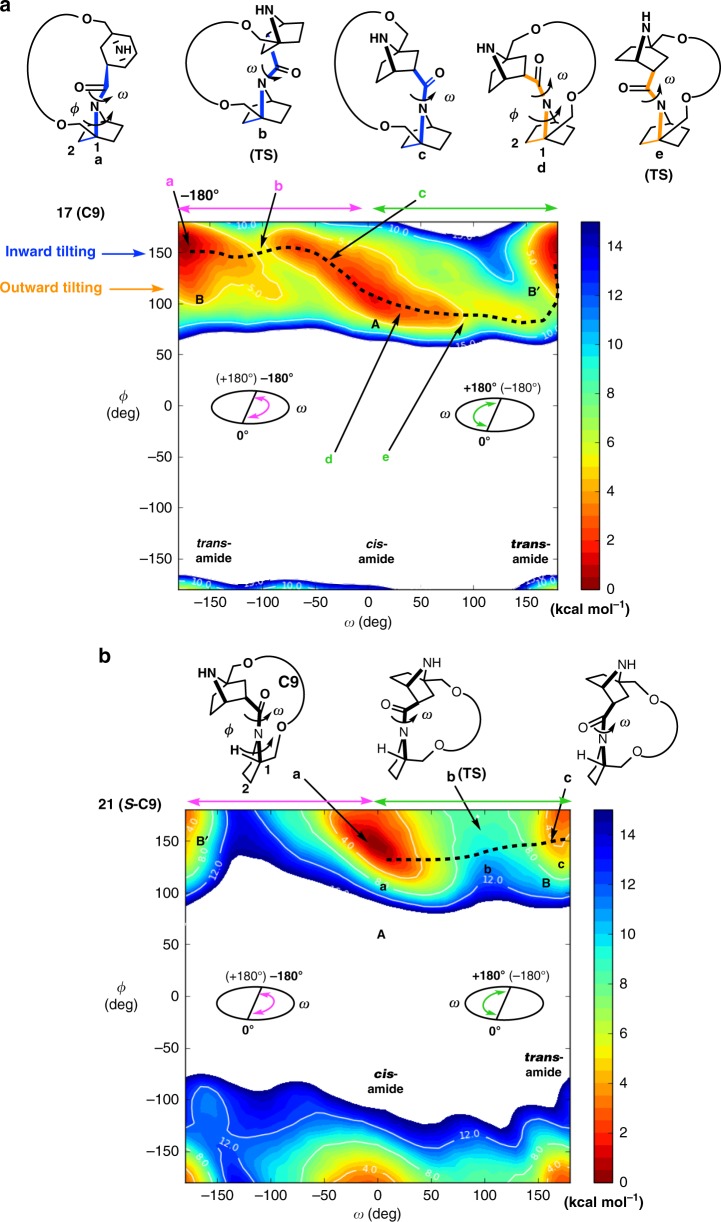
The landscape of lactam amide rotation. **a** Metadymamic simulations of bicycle lactam **17(C9)** in chloroform at 300 K. Dashed lines indicate the rotational pathways. The tilting direction of the pyramidal nitrogen atom of the bicyclic systems is synchronized with the direction of the semicircle-rotation of the amide. **b** Metadymamic simulations of monocycle lactam **21(*S*-C9)** in chloroform at 300 K. Dashed lines indicate the rotational pathways. Rotation of the lactam amide bond is restricted to a south semicircle-rotation angle (e.g., 0° to + 180°)

Metadynamics simulations^41^ of bicyclic amides **17(C9)** and **19(C11)** (Fig. 6a and Supplementary Fig. 54) revealed that the present system is an example in which 360 degree rotation of lactam nitrogen can occur owing to nitrogen pyramidalization: in principle, lactam amide rotation (dihedral angle *ω*, ∠C(bridgehead)NC(O)C in Fig. 1c) is restricted to a south semicircle-rotation angle (e.g., ω:0° to +180°) or a north semicircle-rotation angle (e.g., *ω*:−180° to 0°) **(**Fig. 1c) with respect to the amide dihedral angle (ω) due to the ring coiling (see Fig. 1b), but in the present case (**17(C9)** and **19(C11)**), lactam amide rotation can undergo continuous north semicircle-rotation with inward-tilted amide nitrogen pyramidalization (dihedral angle *ϕ* = 150° in Fig. 6a and Supplementary Fig. 54, B to A)) and at the same time continuous south semicircle-rotation with outward-tilted amide nitrogen pyramidalization (dihedral angle *ϕ* = 100° in Fig. 6a and Supplementary Fig. 54, A to B’). Similar diagrams were obtained in the cases of **16(C8)** and **18(C10)** (for example, Supplementary Fig. 55). X-Ray crystallographic analysis shows that **6**(*Z*)(**C8**) takes *cis*-amide structure with outward-tilted amide nitrogen pyramidalization, consistent with the simulated *cis* amide structures ***d*** in **17(C9)** (Fig. 6a) and **19(C11)** (Supplementary Fig. 54), respectively. In contrast, the *trans* amide of the *N*-tosylated derivative of **8**(*E)* (**Ts-8**(*E*)(**C10**)) (crystal structure, Fig. 5c) takes the structure with inward-tilted amide nitrogen pyramidalization, which is consistent with the simulated *trans* amide structures ***a*** in **17(C9)** (Fig. 6a) and **19(C11)** (Supplementary Fig. 54), respectively. The rotation is dual-directional. The difference between the energy barrier (~4 kcal/mol at **b**) in north semicircle-rotation (**a**→**d**, Fig. 6a: *trans*→*cis*) and the energy barrier (~7 kcal/mol at **e**) in south semicircle-rotation (**d**→**e**, Fig. 6a: *cis*→*trans*) is consistent with the difference in observed rotation speed (*k*_*t*_→_*c*_ > *k*_*c*_→_*t*_) of **17(C9)** (Table 2**)**. As already discussed, the DFT-calculated *cis* amides of **16(C8)**-**19(C11)** also show outward-tilted amide nitrogen pyramidalization (Fig. 4b), while the DFT calculated *trans* amides of **16(C8)**-**19(C11)** show inward-tilted amide nitrogen pyramidalization (Fig. 4a).

On the other hand, metadynamics simulations of virtual monocyclic pyrrolidine amides (**21(*S*-C9)** and **22(*S*-C11)**, Fig. 6b and Supplementary Fig. 56), in which the lower 7-azabicyclo[2.2.1]heptane is replaced with a monocyclic pyrrolidine derivative, show that one dihedral angle around the amide nitrogen atom is allowed, and the rotation with respect to the amide bond is restricted to a south semicircle-rotation angle (e.g., 0° to + 180°, Fig. 6b and Supplementary Fig. 56, A to B): a north semicircle-rotation (e.g., angle *ω*: −180° to 0°, Fig. 6b and Supplementary Fig. 56, B’ to A) is inhibited by the high activation barrier. The *R*-configuration model compounds ((**23(*R*-C9)** and **24(*R*-C11)**, Supplementary Figs. 57 and  58) showed similarly restricted amide rotation. Monocyclic pyrrolidine amides take a planar amide structure, so nitrogen pyramidalization is probably crucial for the 360-degree spinning of lactam amide. A similar restriction in the rotation was also found in the case of the replacement of the lower bicyclic amide with the simple non-cyclic N-methyl amide (**25 (C-9)** and **25 (C-11)**, Supplementary Fig. 3), probably due to the planar amide.

## Discussion

Our findings indicate that side-chain stapling of these bicyclic dimer amides results in monotonous relationships between the rotational rate between *cis* and *trans* lactam amide and the ratio of the *cis*/*trans* isomers, depending on the ring size: as the ring size of the stapled structure is increased, the rotational rate of *trans* to *cis* conformer is decreased and that of *cis* to *trans* conformer is increased, resulting in an increase in the *trans/cis* ratio. Complete inversion of the amide equilibrium, affording an extreme range of *cis/trans* ratios (approximately 1/0~0/1), depending on the linker length, was observed. Our observations and simulations are consistent with the idea that the present lactam amide can spin through 360 degrees due to the occurrence of nitrogen pyramidalization. The tilting direction of the pyramidal nitrogen atom of the bicyclic systems is synchronized with the direction of the semicircle-rotation of the amide (Fig. 1c and d). The present results provided a new insight into the amide rotation of lactams, which are a ubiquitous and divergent class of compounds. The rotational features of lactam amides observed here might be generalized and find applications in molecular design.

## Methods

### Synthesis

Details of synthesis are given in the Supplementary Methods and Supplementary Figs. 18–52.

### Synthesis of 16(C8)

To a solution of **11** (99.6 mg, 0.2030 mmol) in CH_2_Cl_2_ (6 mL) was added TFA (1 mL), and the whole was stirred for 2 h at 0 °C. The reaction mixture was poured into 10% aqueous solution of Na_2_CO_3_. The whole was extracted with CH_2_Cl_2_, dried over Na_2_SO_4_, and evaporated. Column chromatography (CHCl_3_/MeOH = 15/1) gave compound **16(C8)** (45.2 mg, 57%) as a white solid.　M.p.: 101.5 °C–102.0 °C.

^1^H-NMR (CDCl_3_): 4.699 (1 H, t, *J* = 5.2 Hz), 3.983 (1 H, d, *J* = 11.6 Hz), 3.658 (1 H, d, *J* = 11.2 Hz), 3.581–3.446 (6 H, m), 3.356 (1 H, dt, *J* = 4.8 Hz, 8.8 Hz), 3.311–3.254 (1 H, m), 2.20–1.20 (27 H, m). ^13^C-NMR (CD_2_Cl_2_): 170.73, 73.16, 72.04, 71.77, 70.14, 69.10, 67.97, 61.29, 56.79, 48.70, 35.79, 33.47, 32.43, 30.01, 29.06, 28.83, 28.76, 28.46, 28.21, 26.68, 26.21, 25.76. HRMS (ESI, [M + H]+): Calcd. For C_23_H_39_N_2_O_3_^+^: 391.2955, Found: 391.2963.

### Synthesis of 17(C9)

To a solution of **12** (20.6 mg, 0.04082 mmol) in CH_2_Cl_2_ (0.9 mL) was added 0.15 mL of TFA, and the whole was stirred for 1 h at 0 °C and 1 h at rt. The solvent was evaporated and 10% aqueous solution of Na_2_CO_3_ was added to the residue to adjust the pH to 10. The aqueous layer was extracted with CH_2_Cl_2_, dried over Na_2_SO_4_, and evaporated. Column chromatography (CHCl_3_/MeOH = 30/1 to 20/1 to 10/1) and preparative TLC (CHCl_3_/MeOH = 20/1 and 0.1% Et_3_N) gave compound **17(C9)** (8.6 mg, 52%) as a white solid. ^1^H-NMR (CDCl_3_): 4.682 (1 H, brs), 3.960 (1 H, d, *J* = 11.2 Hz), 3.813–3.792 (1 H, m), 3.88–3.72 (1 H, brs), 3.680 (1 H, d, *J* = 10.0 Hz), 3.610 (1 H, d, *J* = 10.0 Hz), 3.569–3.430 (4 H, m), 3.52–3.39 (1 H, m), 2.20–1.18 (28 H, m). ^13^C-NMR (CDCl_3_): 169.50, 72.54, 72.23, 71.81, 69.86, 69.69, 68.11, 61.15, 57.20, 46.61, 35.21, 33.05, 31.53, 30.12, 29.83, 29.27, 28.76, 28.56, 28.42, 27.60, 26.32, 25.27. HRMS (ESI, [M + H]^+^): Calcd. For C_24_H_41_N_2_O_3_^+^: 405.3112, Found: 405.3125.

### Synthesis of 18(C10)

To a solution of **13** (68.2 mg, 0.131 mmol) in CH_2_Cl_2_ (3 mL) was added TFA (0.5 mL) and the whole was stirred for 4 h at 0 °C and 1 h at 10 °C. The reaction mixture was poured into 10% aqueous solution of Na_2_CO_3_. The whole was extracted with CH_2_Cl_2_, dried over Na_2_SO_4_. Then the solvent was evaporated. Column chromatography (CHCl_3_:MeOH = 30:1 to 20:1 to 10:1) and preparative TLC (CHCl_3_/MeOH = 16/1 and 0.2 % NEt_3_) gave **18(C10)** (52.3 mg, 95%) as colorless oil.

^1^H-NMR (50 °C, CD_2_Cl_2_): 4.517 (1 H, brs), 4.116 (1 H, m), 3.888 (1 H, d, *J* = 10.4 Hz), 3.695 (1 H, t, *J* = 4.8 Hz), 3.629 (2 H, *J* = 1.2 Hz), 3.570–3.463 (4 H, m), 3.189 (1 H, m), 2.500–1.272 (31 H, m). ^13^C-NMR (50 °C, CD_2_Cl_2_): 170.82, 74.10, 71.52, 71.49, 70.14, 68.87, 67.95, 60.81, 57.85, 48.83, 36.88, 32.95, 32.75, 31.99, 29.91, 29.47, 29.22, 28.98, 28.62, 26.73, 26.50, 26.09, 23.06. HRMS (ESI, [M + H]^+^): Calcd. For C_25_H_43_N_2_O_3_^+^: 419.3268, Found: 419.3296.

### Synthesis of 19(C11)

To a solution of **14** (37.4 mg, 0.0702 mmol) in CH_2_Cl_2_ (1.8 mL) was added TFA (0.3 mL), and the whole was stirred for 1 h at 0 °C and for 1.5 h at rt. The reaction mixture was poured into 10% aqueous solution of Na_2_CO_3_ and the whole was extracted with CH_2_Cl_2_. The organic layer was dried over Na_2_SO_4_ and evaporated. Column chromatography (AcOEt/MeOH = 20/1 to CHCl_3_/MeOH = 10/1 and 2% Et_3_N) and preparative TLC (CHCl_3_/MeOH = 16/1 and 0.2% Et_3_N) gave **19(C11)** (7.2 mg, 24%) as a colorless oil.

^1^H-NMR (CD_2_Cl_2_, 50 °C): 4.398 (1 H, m), 4.123(1 H, brs), 3.873 (1 H, d, *J* = 10.4 Hz), 3.653 (1 H, t, *J* = 4.8 Hz), 3.561–3.391 (6 H, m), 3.067 (1 H, m), 2.081–1.260 (33 H, m). ^13^C-NMR (CD_2_Cl_2_, 50 °C): 170.97, 74.10, 71.60, 71.37, 70.06, 68.50, 67.98, 60.62, 58.23, 49.34, 36.58, 32.96, 32.48, 30.58, 30.27, 30.10, 29.88, 29.38, 29.31, 29.18, 28.72, 26.90, 26.73, 23.10. HRMS (ESI, [M + H]^+^): Calcd. For C_26_H_45_N_2_O_3_^+^: 433.3425, Found: 433.3429.

### Synthesis of 20(C12)

To a solution of **15** (20.4 mg, 0.03734 mmol) in CH_2_Cl_2_ (0.9 mL) was added TFA (0.15 mL) and the whole was stirred for 5 h at 0 °C and evaporated. 10% Aqueous solution of Na_2_CO_3_ was added to the residue to adjust the pH to 10. The whole was extracted with CH_2_Cl_2_ and the combined organic layer was dried over Na_2_SO_4_ and evaporated. Column chromatography (CHCl_3_/MeOH = 30/1 to 20/1 to 10/1) gave compound **20(C12)** (14.9 mg, 89%) as a white solid.

Mp. 98.0–99.5 °C. ^1^H-NMR (CD_2_Cl_2_): 4.348 (1 H, brs), 4.053 (2 H, s), 3.663–3.644 (1 H, m), 3.575 (2 H, d, *J* = 9.6 Hz), 3.526–3.437 (4 H, m), 3.071–3.021 (1 H, m), 2.20–1.05 (35 H, m). ^13^C-NMR (CD_2_Cl_2_):170.82, 73.52, 71.12, 70.87, 70.06, 68.52, 68.18, 60.60, 58.42, 49.24, 35.95, 32.42, 32.33, 30.46, 30.31, 29.91, 29.72, 29.50, 29.20, 29.08, 28.31, 27.49, 26.73, 26.06. HRMS (ESI, [M + H]^+^): Calcd. For C_27_H_47_N_2_O_3_^+^: 447.3581, Found: 447.3608.

### Single crystal X-ray diffraction experiment

The diffraction experiments for Boc-**(C7)** and **6**(*Z*)**(C8)** were performed in a Bruker APEX II CCD detector (MoKα: *λ* = 0.71073 Å). Structure solution and refinement were performed by using SHELXS-97 and SHELXL-97^42^. The diffraction experiment for **Ts-8**(*E*)**(C10)** was performed in a Rigaku AFC7R (CuKα: *λ* = 1.54178 Å). Absorption correction was performed by psi-scan. Structure solution and refinement were performed with SHELXS-2013^42^ and SHELXL-2013^43^.

### Metadynamics simulations

Metadynamics calculations were performed with Desmond using OPSL3 force field (Schrodinger Inc., U.S.A.). The simulation conditions are as follows: Temperature = 300.0 K, Pressure = 1.01325 bar, Ensemble = NPT, Solvent = CHCl_3_.

## Supplementary Information

Supplementary Information

Supplementary Data 1

## Data Availability

The authors declare that the data supporting the findings of this study are available within the paper and its Supplementary Information files. The X-ray crystallographic coordinates for structures reported in this study have been deposited at the Cambridge Crystallographic Data Centre (CCDC), under deposition numbers 1828742(**6(*****Z*****)(C8)**), 1583295 (**Boc-C7**), 1583296 (**Ts-8(*****E*****)(C10)**). These data can be obtained free of charge from The Cambridge Crystallographic Data Centre via www.ccdc.cam.ac.uk/data_request/cif.
