# Supplementary material for: Amide nitrogen pyramidalization changes lactam amide spinning
Source: Nat Commun. 2019 Jan 28;10:461. doi: 10.1038/s41467-018-08249-9 (PMC6349922; doi:10.1038/s41467-018-08249-9)
Supplement: Supplementary file 1 — Supplementary Information [file 41467_2018_8249_MOESM1_ESM.pdf]

Supplementary Information

Amide nitrogen pyramidalization changes lactam amide spinning

Yuko Otani *et al*

[1] *Graduate School of Pharmaceutical Sciences, University of Tokyo, 7-3-1 Hongo, Bunkyo-ku, Tokyo, 113-0033, Japan*

[2] *Department of Pharmaceutical Sciences at Kagawa Campus, Tokushima Bunri University, 1314-1 Shido, Sanuki, Kagawa 769-2193, Japan.*

E-mail: otani@ mol.f.u-tokyo.ac.jp, ohwada@mol.f.u-tokyo.ac.jp

Table of Contents **Page**

Supplementary Figures 1-3: Additional graphics to the main text

Supplementary Figure 1. Metadynamics calculations of 1-methylazacycloundecan-2-one **S-A**. **3**

Supplementary Figure 2. Metadynamics calculations of a simple secondary amide lactam, a large-ring-sized 1-azacyclononadecan-2-one (19-membered ring) **S-B**. *Cis* and *trans* isomers were separated by high activation barriers. **3**

Supplementary Figure 3. Metadymamics simulations of C9-N-Me amide (**25-C9**) and C11-N-Me amide (**25-C11**), in which the bicyclic amide was replaced with a non-cyclic N-Me tertiary amide. **4**

Supplementary Figure 4. NOESY spectrum of **18(C10)** in CD_2Cl_2 at -53 °C. **5**

Supplementary Figure 5. COSY spectrum of **18(C10)** in CD_2Cl_2 at -53 °C. **5**

Supplementary Figure 6. NOESY spectrum of **17(C9)** in CD_2Cl_2 at -53 °C. **6**

Supplementary Figure 7. NOESY spectrum of **19(C11)** in CD_2Cl_2 at -53 °C. **6**

Supplementary Figure 8. Experimental amide rotation rates k_{ct} and k_{tc} for **17(C9)** and Eyring plots (CD_2Cl_2). **7**

Supplementary Figure 9. Experimental amide rotation rates k_{ct} and k_{tc} for **18(C10)** and Eyring plots (CD_2Cl_2). **7**

Supplementary Figure 10. Experimental amide rotation rates k_{ct} and k_{tc} for **19(C11)** and Eyring plots (CD_2Cl_2). **8**

Supplementary Figure 11. Experimental amide rotation rates k_{ct} and k_{tc} for **16(C8)** and Eyring plots (CD_3OD). **9**

Supplementary Figure 12. Experimental amide rotation rates k_{ct} and k_{tc} for **17(C9)** and Eyring plots (CD_3OD). **9**

Supplementary Figure 13. Experimental amide rotation rates k_{ct} and k_{tc} for **18(C10)** and Eyring plots (CD_3OD). **10**

Supplementary Figure 14. X-ray Crystal Structure and Crystallographic Data of Boc-**(C7)**. **11**

Supplementary Figure 15. X-ray Crystal Structure and Crystallographic Data of **6**(*Z*)**(C8)**. **12**

Supplementary Figure 16. X-ray Crystal Structure and Crystallographic Data of **Ts-8**(*E*)**(C10)**. **13**

Supplementary Figure 17. The ratio of cis-amide of the linked Boc-dimers in $CDCl_3$ at -28.4 °C. **14**

Supplementary Methods

1) General methods and the detail of Synthesis **15**

2) Computational Studies **15**

Supplementary Figure 18. Synthesis of the bridgehead-substituted uncyclized dimers **1-5** **17**

Supplementary Figure 19-52 ^{1}H and ^{13}C-NMR Charts of synthesized compounds **39**

Supplementary Figure 53. DFT-calculated structures of the transition structure of amide bond rotation of **17(C9), 18(C10)** and **19(C11)**. **73**

Supplementary Table 1. The relative free energy ($\Delta\Delta G_{cis\text{-}trans}$) (kcal/mol) and the free energy of rotation from trans to cis ($\Delta G_{t\text{-}c}^{\ddagger}$) of **17(C9)**, **18(C10)**, and **19(C11)** in CH_2Cl_2 at 25 °C. **73**

Supplementary Table 2. The relative free energy ($\Delta\Delta G_{cis\text{-}trans}$) (kcal/mol) and the free energy of rotation from cis to trans ($\Delta G_{c\text{-}t}^{\ddagger}$) of **16(C8)**, **17(C9)** and **18(C10)** in methanol. **74**

Supplementary Figure 54. Metadymamic simulations of the landscape of bicyclic lactam amide rotation. Bicycle lactam **19(C11).** **75**

Supplementary Figure 55. Metadymamic simulations of the landscape of bicyclic lactam amide rotation. Bicycle lactam **16(C8)**. **76**

Supplementary Figure 56. **T**he landscape of lactam amide rotation of monocycle lactam **22(*S*-C11)**. **77**

Supplementary Figure 57. Model reference planar amide lactams for metadymamic simulations **78**

Supplementary Figure 58. Metadymamic simulations of *R*-isomers **23 (R-9)** and **24 (R-11)** **78**

Supplementary References **79**

Supplementary Figures : Additional graphics to the main text

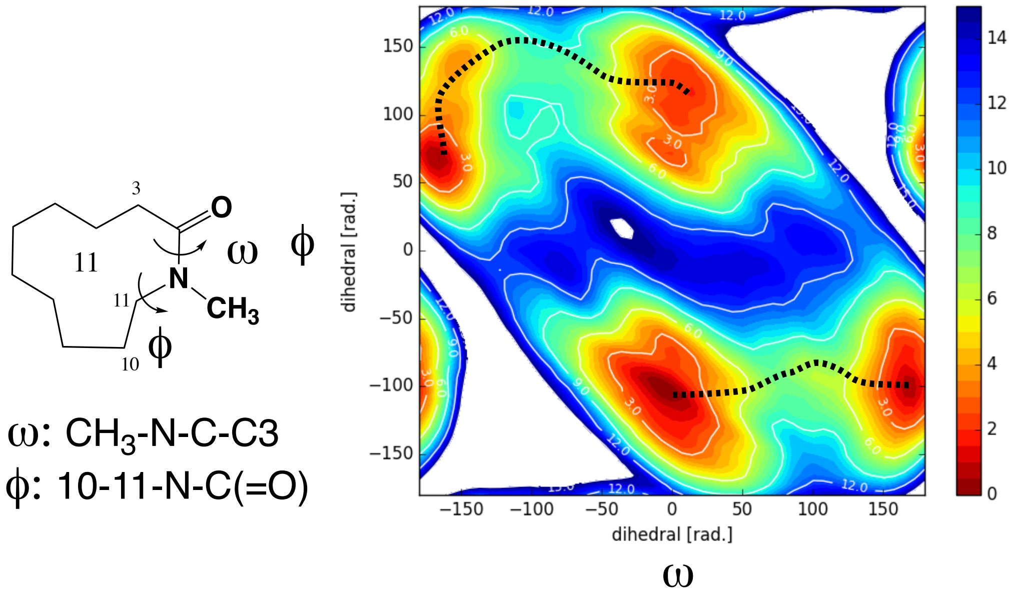

Supplementary Figure 1. Metadynamics calculations of 1-methylaza-cycloundecan-2-one (11-membered ring) **S-A**. ($CHCl_3$, 300K, 30 nsec, NPT)

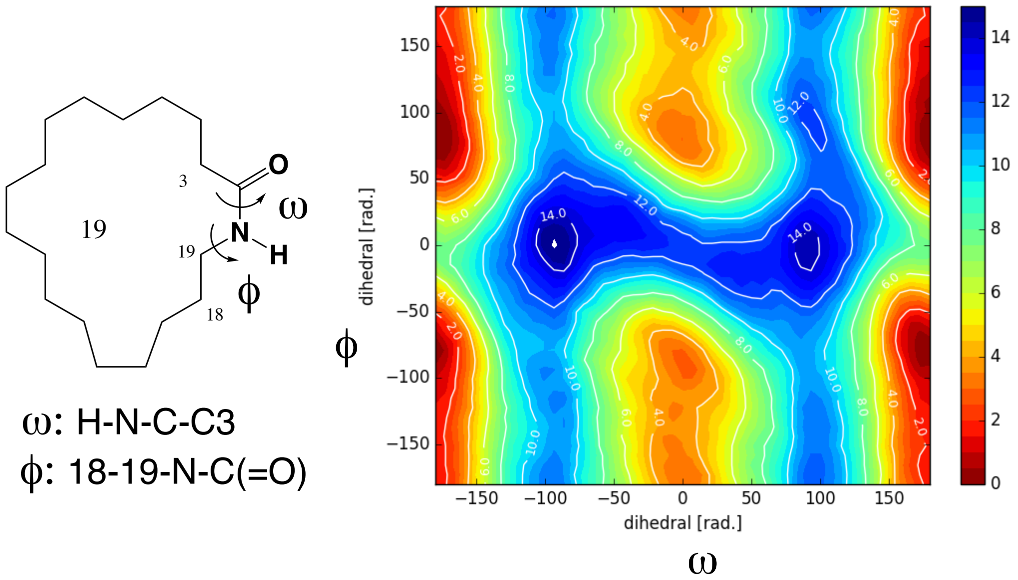

Supplementary Figure 2. Metadynamics calculations of a simple secondary amide lactam, a large-ring-sized 1-azacyclononadecan-2-one (19-membered ring) **S-B**. *Cis* and *trans* isomers were separated by high activation barriers.

a

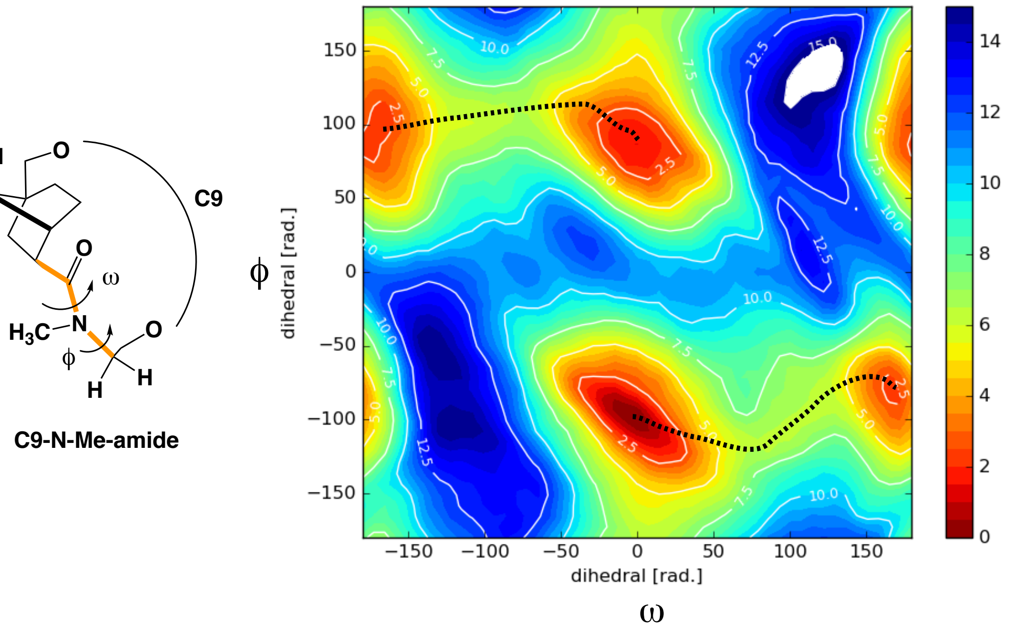

b

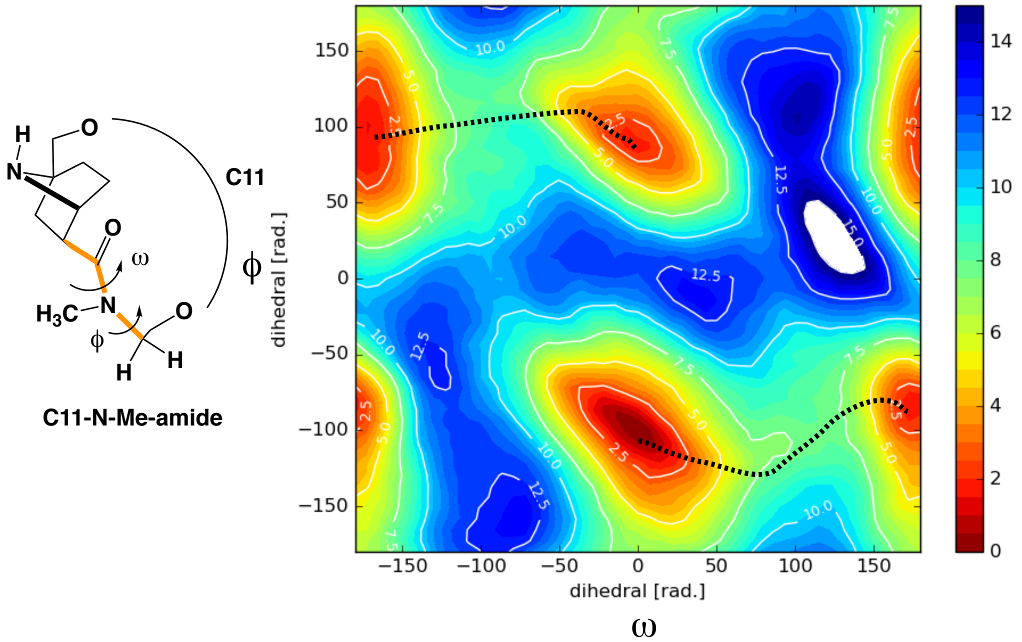

Supplementary Figure 3. Metadymamics simulations of C9-N-Me amide (**25-C9**) (**a**) and C11-N-Me amide (**25-C11**) (**b**), in which the bicyclic amide was replaced with a non-cyclic N-Me tertiary amide.

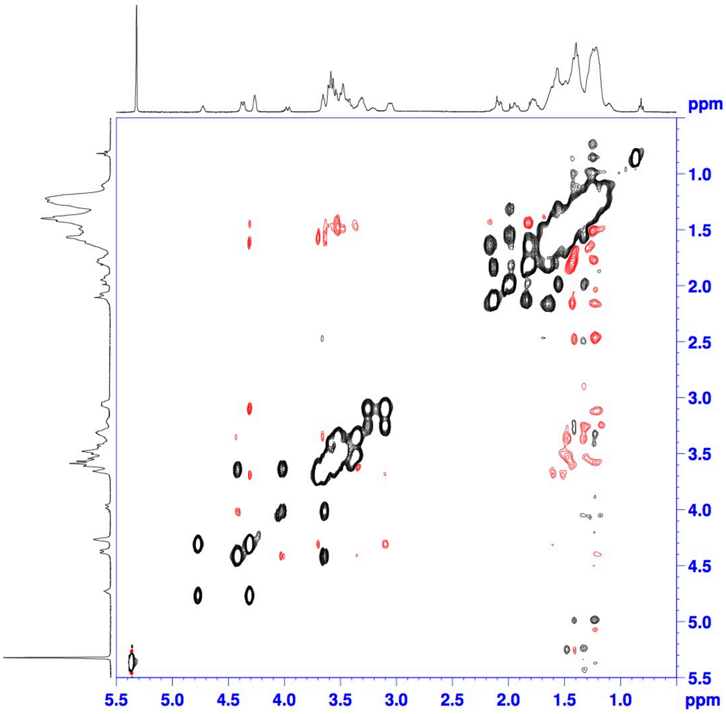

Supplementary Figure 4. NOESY spectrum of **18(C10)** in CD_2Cl_2 at -53 °C (mixing time = 300 ms).

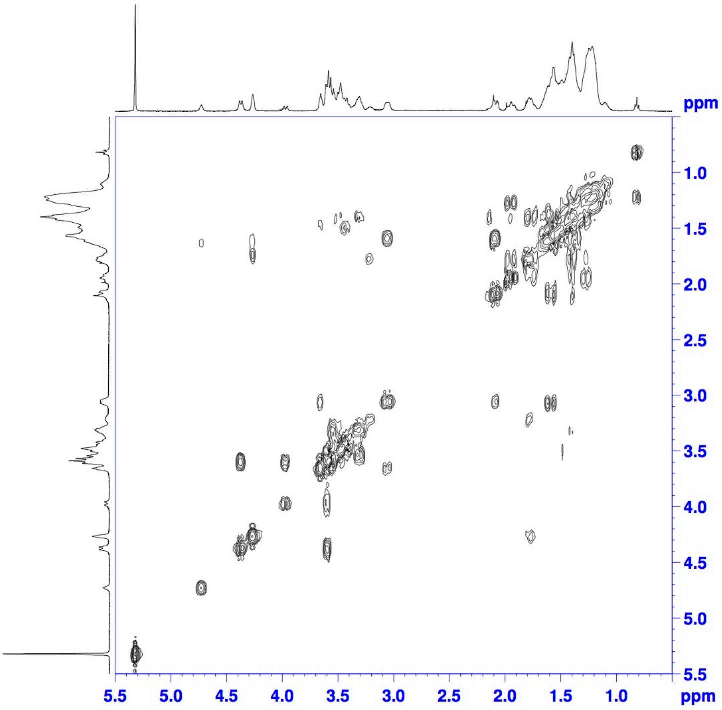

Supplementary Figure 5. COSY spectrum of **18(C10)** in CD_2Cl_2 at -53 °C**.**

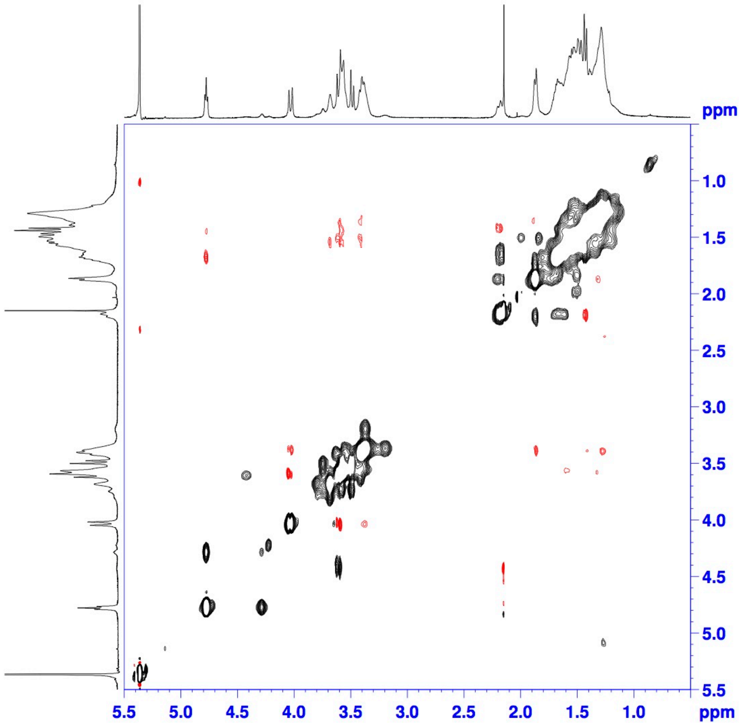

Supplementary Figure 6. NOESY spectrum of **17(C9)** in CD_2Cl_2 at -53 °C (mixing time = 300 ms).

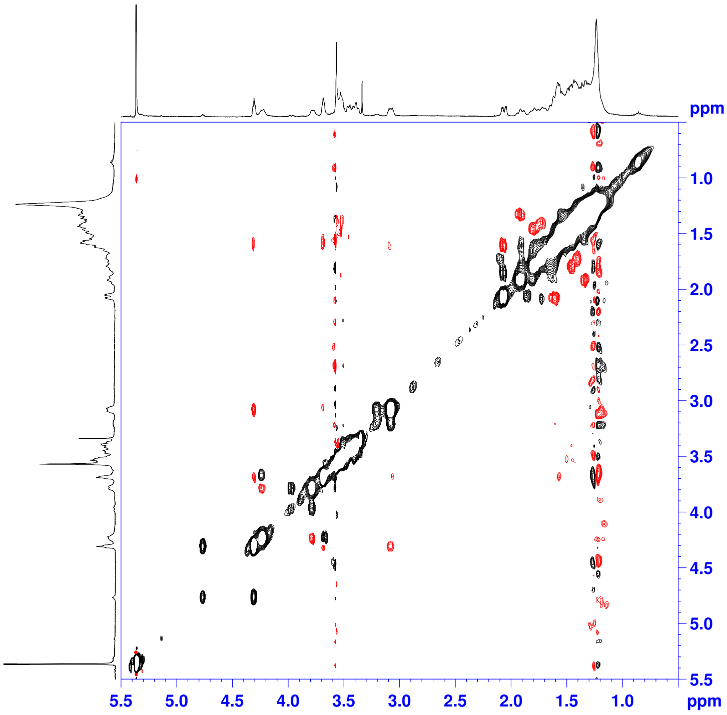

Supplementary Figure 7. NOESY spectrum of **19(C11)** in CD_2Cl_2 at -53 °C (mixing time = 300 ms).

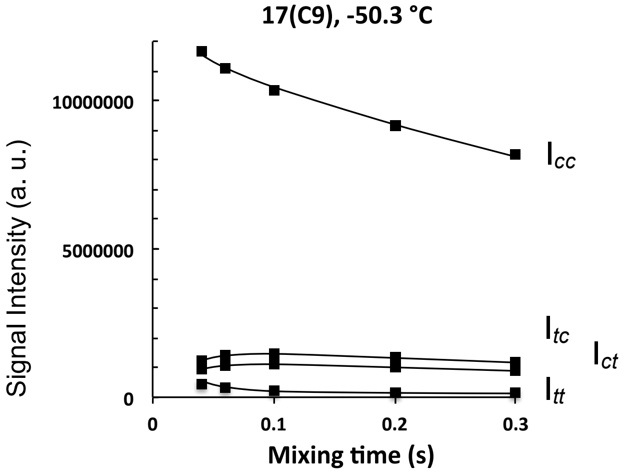

Temperature	k_{tc}	k_{ct}
-47.8	44.03	4.07
-50.3	33.88	3.57
-52.8	27.16	3.02
-55.3	21.95	2.72
-57.8	18.62	2.23

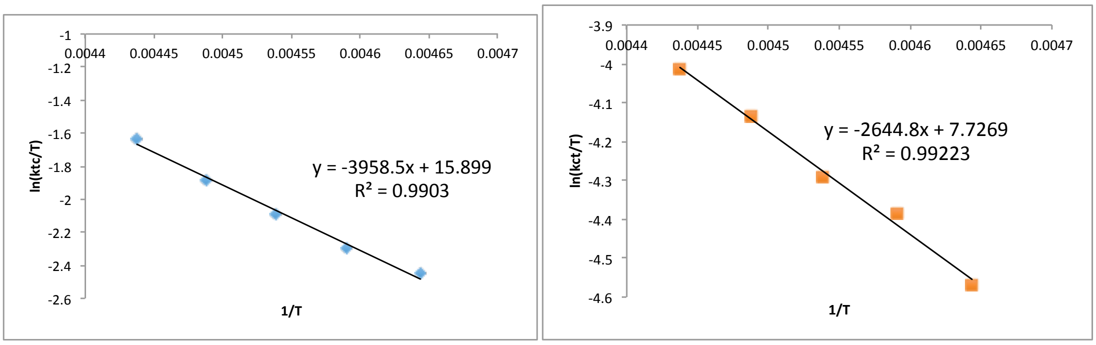

Supplementary Figure 8. Experimental amide rotation rates for **17(C9)** and Eyring plots (CD_2Cl_2).

Temperature	k_{tc}	k_{ct}
-47.8	5.52	16.81
-50.3	3.85	11.41
-52.8	3.43	10.37
-55.3	2.52	7.93
-57.8	1.96	5.68

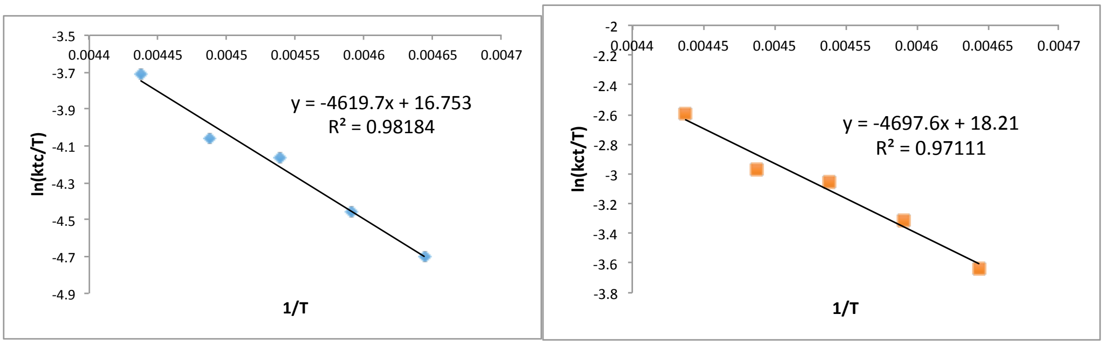

Supplementary Figure 9. Experimental amide rotation rates for **18(C10)** and Eyring plots (CD_2Cl_2).

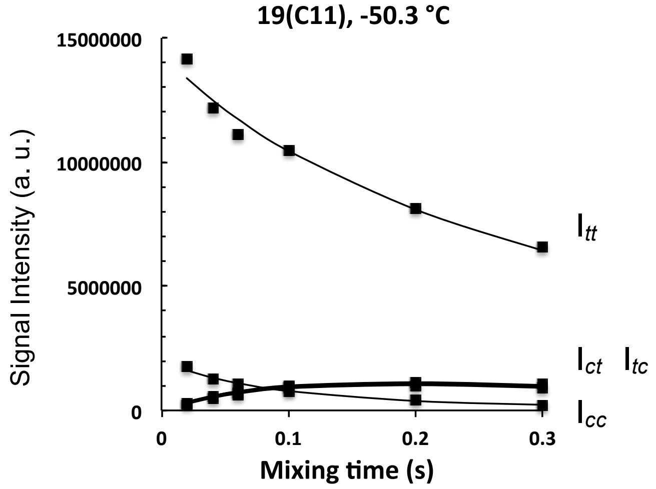

Temperature	k_{tc}	k_{ct}
-47.8	1.48	12.07
-50.3	1.24	9.94
-52.8	0.81	6.27
-55.3	0.67	4.96
-57.8	0.47	3.23

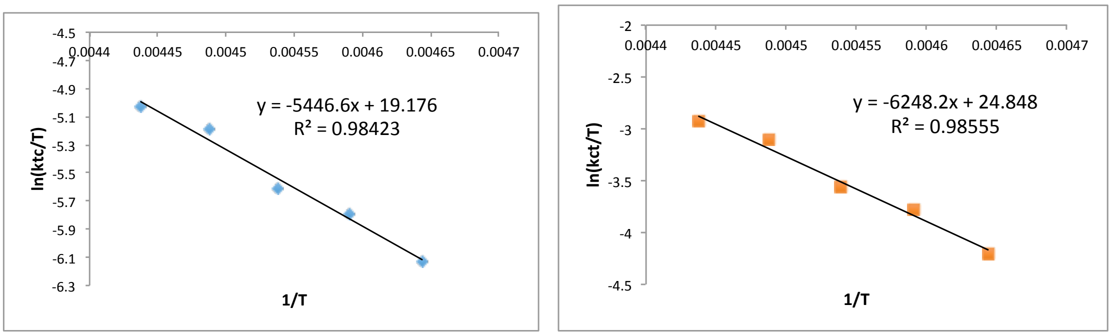

Supplementary Figure 10. Experimental amide rotation rates for **19(C11)** and Eyring plots (CD_2Cl_2).

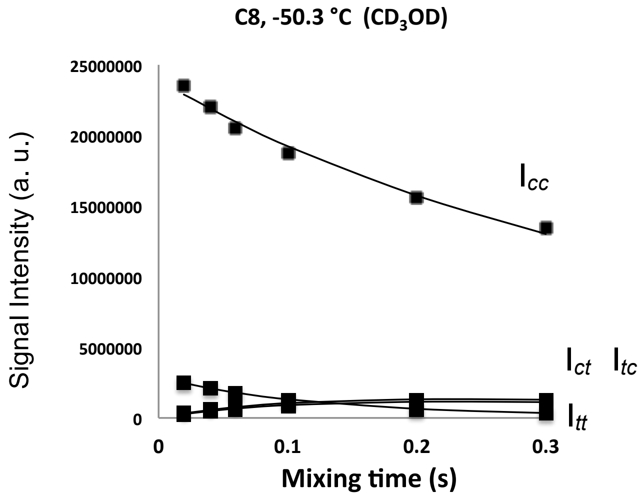

Temperature	k_{tc}	k_{ct}
-34.9	34.422	2.334
-38.7	22.597	2.007
-42.6	14.960	1.337
-46.4	10.170	0.894
-50.3	5.999	0.623

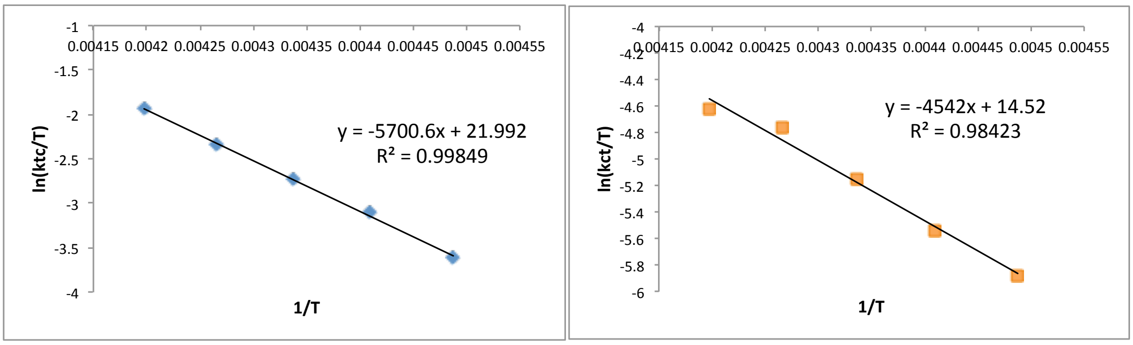

Supplementary Figure 11. Experimental amide rotation rates for **16(C8)** and Eyring plots (CD_3OD).

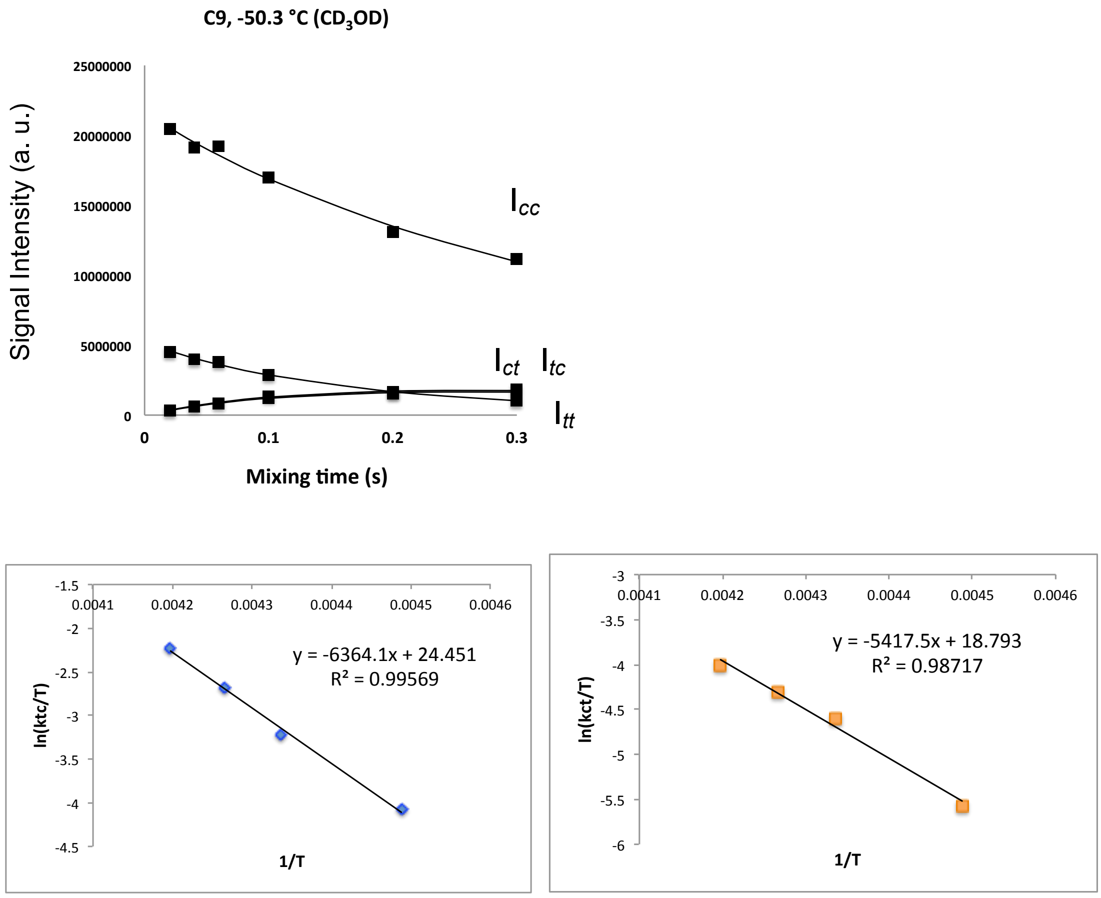

Supplementary Figure 12. Experimental amide rotation rates for **17(C9)** and Eyring plots (CD_3OD).

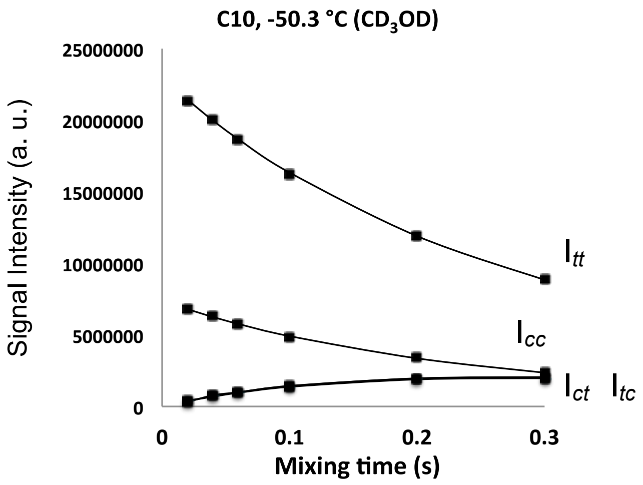

Temperature	k_{tc}	k_{ct}
-47.8	1.146	3.692
-50.3	0.876	2.832
-52.8	0.660	1.979
-55.3	0.464	1.448
-57.8	0.327	0.849

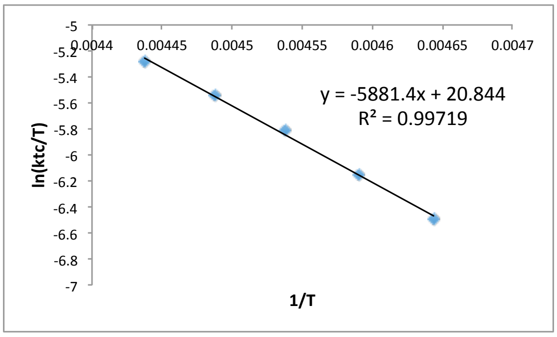

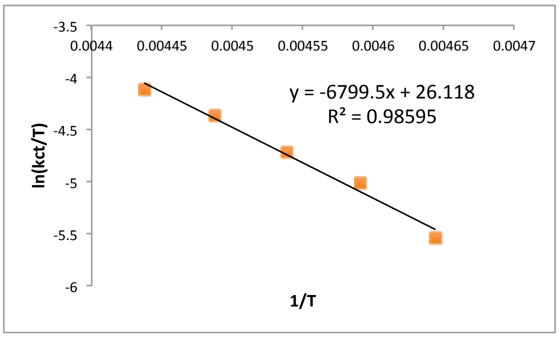

Supplementary Figure 13. Experimental amide rotation rates for **18(C10)** and Eyring plots (CD_3OD).

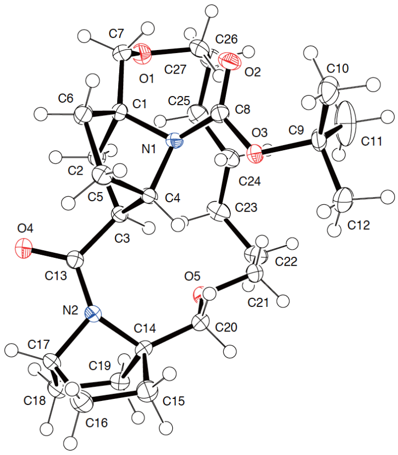

Empirical formula	C27 H44 N2 O5	
Formula weight	476.64	
Temperature	100 K	
Wavelength	0.71073 Å	
Crystal system	Triclinic	
Space group	P-1	
Unit cell dimensions	a = 8.8250(15) Å	α= 88.065(2)°.
	b = 11.703(2) Å	β= 81.153(2)°.
	c = 13.708(2) Å	γ = 71.658(2)°.
Volume	1327.7(4) $Å^3$	
Z	2	
Density (calculated)	1.192 Mg/m^3	
Absorption coefficient	0.081 mm^{-1}	
F(000)	520	
Crystal size	0.40 x 0.25 x 0.20 mm^3	
Theta range for data collection	1.50 to 25.95°.	
Index ranges	-10<=h<=10, -13<=k<=14, -16<=l<=16	
Reflections collected	11889	
Independent reflections	4657 [R(int) = 0.0374]	
Completeness to theta = 25.00°	97.0 %	
Absorption correction	None	
Refinement method	Full-matrix least-squares on F^2	
Data / restraints / parameters	4657 / 0 / 310	
Goodness-of-fit on F^2	1.050	
Final R indices [I>2sigma(I)]	R1 = 0.0397, wR2 = 0.1034	
R indices (all data)	R1 = 0.0456, wR2 = 0.1086	
Largest diff. peak and hole	0.219 and -0.247 e.$Å^{-3}$	

Supplementary Figure 14. X-ray Crystallographic Data of **Boc-C7** (CCDC 1828742)

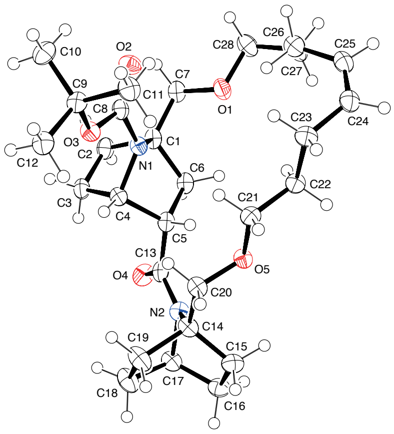

Empirical formula	C28 H44 N2 O5	
Formula weight	488.65	
Temperature	100(2) K	
Wavelength	0.71073 Å	
Crystal system	Monoclinic	
Space group	P21/c	
Unit cell dimensions	a = 11.3285(19) Å	α= 90°.
	b = 12.438(2) Å	β= 117.444(6)°.
	c = 21.523(3) Å	γ = 90°.
Volume	2691.4(7) $Å^3$	
Z	4	
Density (calculated)	1.206 Mg/m^3	
Absorption coefficient	0.082 mm^{-1}	
F(000)	1064	
Crystal size	0.50 x 0.40 x 0.20 mm^3	
Theta range for data collection	1.95 to 25.94°.	
Index ranges	-13<=h<=13, -15<=k<=14, -26<=l<=24	
Reflections collected	23479	
Independent reflections	4934 [R(int) = 0.0429]	
Completeness to theta = 25.00°	99.2 %	
Absorption correction	None	
Refinement method	Full-matrix least-squares on F^2	
Data / restraints / parameters	4934 / 0 / 319	
Goodness-of-fit on F^2	1.049	
Final R indices [I>2sigma(I)]	R1 = 0.0347, wR2 = 0.0863	
R indices (all data)	R1 = 0.0400, wR2 = 0.0903	
Largest diff. peak and hole	0.212 and -0.235 e.$Å^{-3}$	

Supplementary Figure 15. X-ray Crystallographic Data of **6(Z)(C8)** (CCDC 1583295)

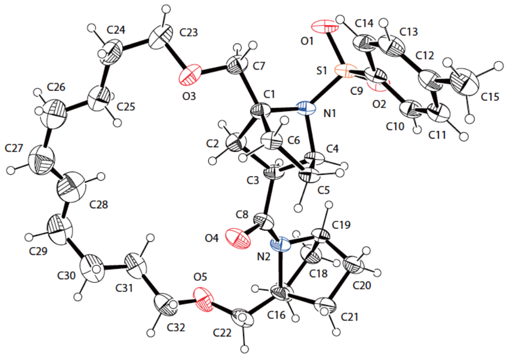

Empirical formula	C32 H46 N2 O5 S	
Formula weight	570.77	
Temperature	173 K	
Wavelength	1.54178 Å	
Crystal system	Monoclinic	
Space group	P 21/c	
Unit cell dimensions	a = 16.123(20) Å	α= 90°.
	b = 11.54(5) Å	β= 106.56(7)°.
	c = 17.320(13) Å	γ = 90°.
Volume	3090(15) Å^3	
Z	4	
Density (calculated)	1.227 Mg/m^3	
Absorption coefficient	1.261 mm^{-1}	
F(000)	1232	
Crystal size	0.20 x 0.20 x 0.02 mm^3	
Theta range for data collection	2.859 to 68.082°.	
Index ranges	-19<=h<=19, -13<=k<=13, -20<=l<=20	
Reflections collected	11369	
Independent reflections	5616 [R(int) = 0.0935]	
Completeness to theta = 67.679°	100.0 %	
Absorption correction	Psi-scan	
Max. and min. transmission	0.975 and 0.873	
Refinement method	Full-matrix least-squares on F^2	
Data / restraints / parameters	5616 / 51 / 362	
Goodness-of-fit on F^2	0.992	
Final R indices [I>2sigma(I)]	R1 = 0.0604, wR2 = 0.1593	
R indices (all data)	R1 = 0.1761, wR2 = 0.2048	
Extinction coefficient	n/a	
Largest diff. peak and hole	0.416 and -0.286 e.Å^{-3}	

Supplementary Figure 16. X-ray Crystallographic Data of **Ts-8(*E*)(C10)** (CCDC 1583296)

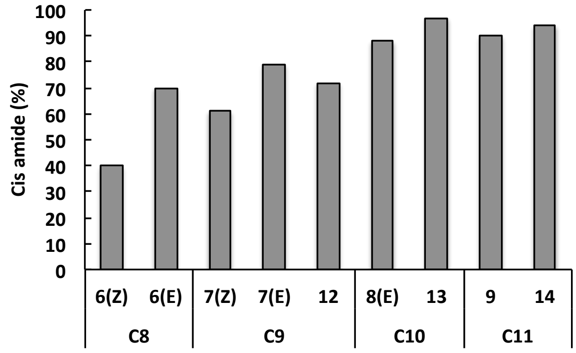

	Linker length	*cis/trans* (%)
**6**(*Z*)	**C8**	40/60
**6**(*E*)	**C8**	70/30
**7**(*Z*)	**C9**	61/39
**7**(*E*)	**C9**	79/21
**12**	**C9**	72/28
**8**(*E*)	**C10**	88/12
**13**	**C10**	97/3
**9**	**C11**	90/10
**14**	**C11**	94/6

Supplementary Figure 17. The ratio of cis-amide of the linked Boc-dimers in $CDCl_3$ at -28.4 °C.

Supplementary Methods

General Methods of Synthesis

Unless stated otherwise, commercial grade reagents were used without further purification. Open column chromatography was carried out using Kanto chemical silica gel (silica gel 60 N (100-210 μm)). One- and two-dimensional ^{1}H-NMR (400 MHz) spectra and ^{13}C-NMR (100 MHz) spectra were recorded on a Bruker Avance 400 NMR spectrometer running Topspin. The spectra were recorded at 20 °C, unless otherwise noted. ^{1}H-NMR and ^{13}C-NMR chemical shifts (δ) are given in parts per million (ppm) and coupling constants are given in hertz (Hz). s = singlet, brs = broad singlet, d = doublet, t = triplet, m = multiplet. ^{1}H-NMR spectra are reported relative to residual solvent signals ($CDCl_3$: 7.26 ppm). Data for ^{13}C-NMR spectra are reported in terms of chemical shift (ppm) relative to residual solvent peak ($CDCl_3$: 77.0 ppm). The EXSY spectra were recorded with 6 mixing times (T_m) of 20, 40, 60, 100, 200, and 300 ms at 215.4 K, 217.9 K, 220.4 K, 222.9 K, and 225.4 K in $CDCl_3$ and at 222.8 K, 226.8 K, 230.6 K, 234.4 K, and 238.2 K in CD_3OD. The 90° pulse widths were estimated by a standard method. The temperature was calibrated with methanol as a reference by using a standard method.[1] Electron spray ionization time-of-flight mass spectra (ESI-TOF MS) were recorded on a Bruker micrOTOF-05. The combustion analysis was carried out in the microanalytical laboratory of the University of Tokyo. All of the melting points were measured with a Yanaco Micro Melting Point Apparatus without correction.

Compounds **21** and **26** were synthesized according to a modified procedure of reference 2.

Computational Studies

DFT Calculations Conformation search of the ground state was performed with the Monte Carlo multiple minimum (MCMM) method with MacroModel 8.6 program with an energy window of 21 kJ/mol using the OPLS-2005/OPLS3 force fields to verify the ground state. The geometries of the *cis*-amide and *trans*-amide structures and transition states (TS) of rotation were fully optimized at the B3LYP/6-31G(d) or M06-2X/6-31G(d) level with the Gaussian 16 program.[3] Harmonic vibrational frequency computations characterized the optimized structures. The zero-point vibrational energy corrections were done without scaling. Single point energies were

calculated with M06-2X/6-311++G(d,p). Bulk solvation effects (self-consistent reaction field, SCRF) were simulated by the SMD method in dichloromethane or methanol.

Metadynamics Simulations All the metadynamics calculations were performed with Desmond (version 2017-04) using OPSL3 force field (Schrodinger Inc., U.S.A.). The simulation conditions are as follows: Temperature=300.0 K, Pressure=1.01325 bar, Ensemble=NPT, Solvent=$CHCl_3$.

a

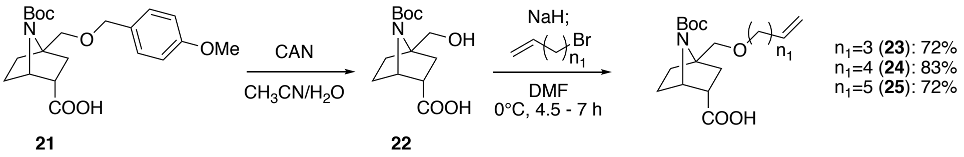

b

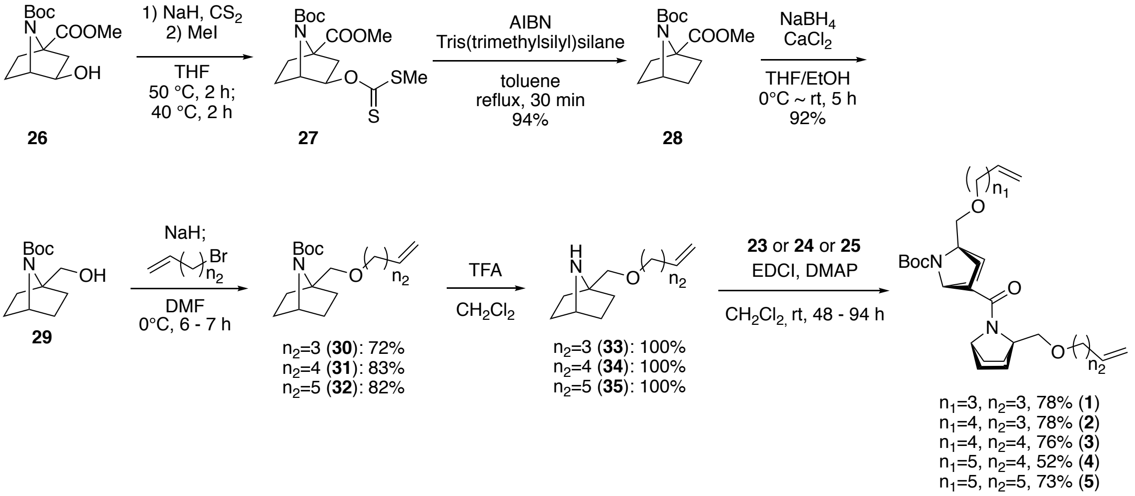

c

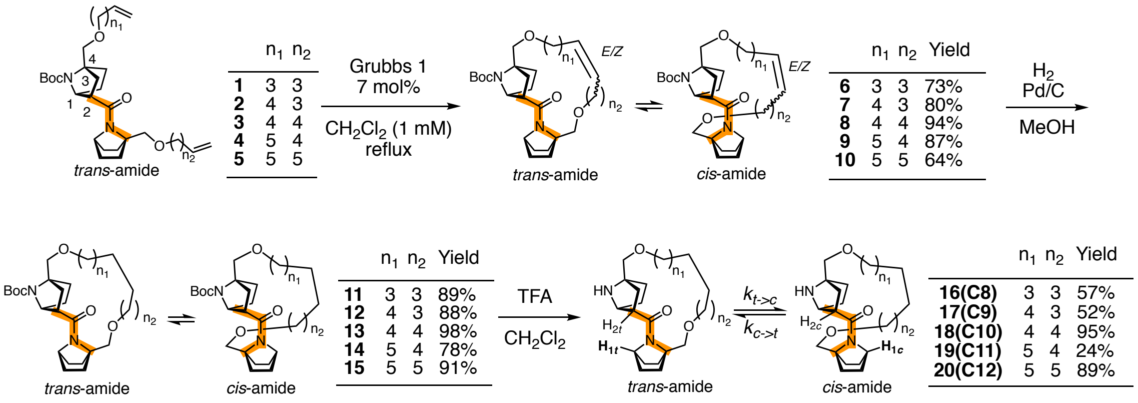

Supplementary Figure 18. Synthesis of the bridgehead-substituted uncyclized dimers and side-chain stapled lactams.

Synthesis of 22

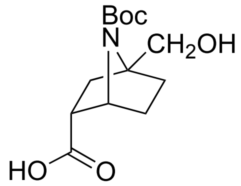

To a solution of **21** (900.0 mg, 2.3006 mmol) in CH_3CN (40 mL) and H_2O (10 mL), CAN (5.0457 g, 9.2025 mmol) was added. The reaction mixture was stirred for 40 min at rt. Then saturated aqueous solution of $NaHCO_3$ was added to the mixture and the organic solvent was evaporated. The residual solution was washed with AcOEt. The aqueous phase was acidified to pH = 2 by adding 5% aqueous solution of $KHSO_4$, and extracted with AcOEt. The combined organic layer was dried over Na_2SO_4, and the solvent was evaporated to give compound **22** (615.5 mg, 99%) as a colorless oil. ^{1}H-NMR ($CDCl_3$): 4.467-4.443 (1H, m, bridgehead-*H*), 3.948-3.878 (2H, m, -C*H*$_2$-OH), 3.073-3.015 (1H, m, *H*-C(2)), 2.11-2.00 (1H, m), 1.90-1.60 (4H, m), 1.55-1.35 (1H, m), [C*H*$_2$×3] 1.439 (9H, s, -C(C*H*$_3$)$_3$). ^{13}C-NMR ($CDCl_3$): 176.61, 155.04, 81.28, 70.05, 61.52, 60.18, 46.01, 34.25, 31.46, 28.41, 25.31. HRMS (ESI, [M-H]$^-$): Calcd. for $C_{13}H_{20}NO_5^-$, 270.1347. Found: 270.1392. Anal. Calcd. for $C_{13}H_{21}NO_5{\cdot}0.2H_2O$: C, 56.80; H, 7.85; N, 5.09. Found: C, 56.66; H, 7.49; N, 5.06.

Synthesis of 23

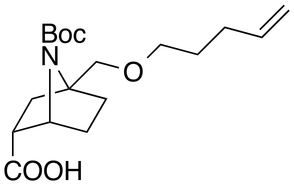

To a suspension of NaH (472.6 mg, 11.815 mmol, ca. 60%) in DMF (8 mL), a solution of **22** (400.7 mg, 1.4770 mmol) in DMF (7 mL) was added dropwise at 0 °C. The reaction mixture was stirred for 15 min at 0 °C. To the reaction mixture were added TBAI (54.5 mg, 0.1475 mmol) in one portion and 5-bromo-1-pentene (344.6 μL, 2.954 mmol) dropwise at 0 °C. The reaction mixture was stirred for 1 h at 0 °C. Then another portion of 5-bromo-1-pentene (172.3 μL, 1.477 mmol) was added to the reaction mixture. After 1 h at 0 °C, the third portion of 5-bromo-1-pentene (258.5 μL, 2.2155 mmol) was added to the reaction mixture. After 1 h at 0 °C, the forth portion of 5-bromo-1-pentene (172.3 μL, 1.477 mmol) was added to the reaction mixture. After 1

h at 0 °C, the fifth portion of 5-bromo-1-pentene (172.3 μL, 1.477 mmol) was added to the reaction mixture. The reaction mixture was stirred for 30 min at 0 °C, and was poured into cold 5% aqueous solution of $KHSO_4$. The mixture was extracted with AcOEt. The organic layer was dried over Na_2SO_4 and evaporated. Column chromatography (n-hexane / AcOEt = 3 / 2 and 1% AcOH then n-hexane / AcOEt = 2 / 3 and 1% AcOH) gave **23** (360.2 mg, 72%) as a colorless oil and **22** (84.8 mg, 21% recovery). ^{1}H-NMR ($CDCl_3$, A mixture with a small amount of the C(2)-*exo*-isomer, *endo/exo*=93/7): 5.847-5.762 (1H, m), 5.034-4.932 (2H, m) [CH=CH_2], 4.634-4.622 (0.066H, m, bridgehead-*H*, *exo*), 4.510-4.486 (0.934H, m, bridgehead-*H*, *endo*), 4.046-3.870 (2H, m, -CH_2-O-), 3.507 (3H, t, J=6.4 Hz, -OCH_2-), 3.134-3.080 (0.934H, m, *H*-C(2), *endo*), 2.633-2.597 (0.066H, m, *H*-C(2), *exo*), 2.20-1.37 (10H, m), [-CH_2-], 1.444 (8.406H, s, -C(CH_3)$_3$ of *endo* isomer), 1.403 (0.594H, s, -C(CH_3)$_3$ of *exo* isomer). ^{13}C-NMR ($CDCl_3$): 178.19, 155.09, 138.53, 114.79, 80.36, 72.48, 71.04, 68.53, 60.58, 45.52, 36.22, 33.34, 30.42, 28.91, 28.46, 24.89. HRMS (ESI, [M-H]$^-$): Calcd. for $C_{18}H_{28}NO_5^-$, 338.1973. Found: 332.2002. Anal. Calcd. for $C_{18}H_{29}NO_5 \cdot 0.2H_2O$: C, 63.02; H, 8.64; N, 4.08. Found: C, 62.91; H, 8.48; N, 4.04.

Synthesis of 24

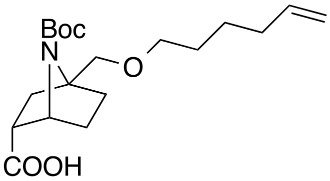

To a suspension of NaH (787.4 mg, 19.685 mmol, ca. 60%) in DMF (8 mL), a solution of **22** (534.4 mg, 1.9698 mmol) in DMF (12 mL) was added dropwise at 0 °C. The reaction mixture was stirred for 15 min at 0 °C. To the reaction mixture were added TBAI (72.7 mg, 0.1968 mmol) in one portion and 6-bromo-1-hexene (395 μL, 2.955 mmol) dropwise at 0 °C. The reaction mixture was stirred for 20 min at 0 °C. Then another portion of 6-bromo-1-hexene (263 μL, 1.9698 mmol) was added to the reaction mixture. After 2 h at 0 °C, the third portion of 6-bromo-1-hexene (263 μL, 1.9698 mmol) was added to the reaction mixture. After 1 h at 0 °C, the forth portion of 6-bromo-1-hexene (263 μL, 1.9698 mmol) was added to the reaction mixture. After 1 h at 0 °C, the fifth portion of 6-bromo-1-hexene (263 μL, 1.9698 mmol) was added to the reaction mixture. The reaction mixture was stirred for 1.5 h at 0 °C and poured into cold

5% aqueous solution of $KHSO_4$. The mixture was extracted with AcOEt. The combined organic layer was dried over Na_2SO_4 and evaporated. Column chromatography (n-hexane / AcOEt = 3 / 2 and 1% AcOH then n-hexane / AcOEt = 2 / 3 and 1% AcOH) gave **24** (579.3 mg, 83%) as a colorless oil. ^{1}H-NMR ($CDCl_3$, A mixture with a small amount of the C(2)-*exo*-isomer, *endo/exo*=92/8): 5.832-5.748 (1H, m), 5.019-4.918 (2H, m) [CH=CH$_2$], 4.632-4.620 (0.081H, m, bridgehead-*H*, *exo*), 4.507-4.484 (0.919H, m, bridgehead-*H*, *endo*), 4.044-3.869 (2H, m, -C*H*$_2$-O-), 3.499 (3H, t, J=6.4Hz, -OC*H*$_2$-), 3.130-3.077 (0.919H, m, *H*-C(2), *endo*), 2.628-2.593 (0.081H, m, *H*-C(2), *exo*), 2.18-1.38 (12H, m), [-C*H*$_2$-], 1.443 (8.271H, s, -C(C*H*$_3$)$_3$ of *endo* isomer), 1.401 (0.729H, s, -C(C*H*$_3$)$_3$ of *exo* isomer). ^{13}C-NMR ($CDCl_3$): 178.18, 155.10, 138.99, 114.60, 80.35, 72.45, 71.64, 68.55, 60.59, 45.52, 36.22, 33.68, 33.34, 29.18, 28.47, 25.59, 24.89. HRMS (ESI, [M-H]$^-$): Calcd. for $C_{19}H_{30}NO_5^-$, 352.2129. Found: 352.2167. Anal. Calcd. for $C_{19}H_{31}NO_5 \cdot 0.1H_2O$: C, 64.24; H, 8.85; N, 3.94. Found: C, 63.95; H, 8.69; N, 3.83.

Synthesis of 25

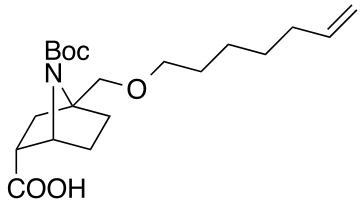

To a suspension of NaH (64.9 mg, 1.6224 mmol, ca. 60%) in DMF (0.5 mL), a solution of **22** (52.3 mg, 0.1928 mmol) in DMF (1 mL) was added dropwise at 0 °C. The reaction mixture was stirred for 10 min at 0 °C. To the reaction mixture were added TBAI (7.5 mg, 0.0203 mmol) in one portion and 7-bromo-1-heptene (58 μL, 0.3856 mmol) dropwise at 0 °C. The reaction mixture was stirred for 1 h at 0 °C. Then another portion of 7-bromo-1-heptene (29 μL, 0.1928 mmol) was added to the reaction mixture. After 1 h at 0 °C, the third portion of 7-bromo-1-heptene (29 μL, 0.1928 mmol) was added to the reaction mixture. After 1 h at 0 °C, the forth portion of 7-bromo-1-heptene (29 μL, 0.1928 mmol) was added to the reaction mixture. After 1 h at 0 °C, the fifth portion of 7-bromo-1-heptene (29 μL, 0.1928 mmol) was added to the reaction mixture. After 1 h at 0 °C, the sixth portion of 7-bromo-1-heptene (29 μL, 0.1928 mmol) was added to the reaction mixture. The reaction mixture was stirred for 1 h at 0 °C and poured into cold 5% aqueous solution of $KHSO_4$. The mixture was extracted with AcOEt. The combined organic layer was dried over Na_2SO_4 and evaporated. Column

chromatography (n-hexane / AcOEt = 2 / 1 and 1% AcOH) gave compound **25** (50.7 mg, 72%) as a colorless oil. ^{1}H-NMR ($CDCl_3$, A mixture with a small amount of the C(2)-*exo*-isomer, *endo/exo*=91/9): 5.840-5.739 (1H, m), 5.001-4.900 (2H, m) [$CH=CH_2$], 4.626-4.614 (0.086H, m, bridgehead-*H* of *exo* isomer), 4.501-4.479 (0.914H, m, bridgehead-*H* of *endo* isomer), 3.972 (1H, d, J=9.6 Hz, -CH_2O-), 3.925 (1H, d, J=9.6 Hz, -CH_2O-), 3.483 (2H, t, J=6.8 Hz, -OCH_2-), 3.122-3.069 (0.914H, m, *H*-C(2) of *endo* isomer), 2.623-2.587 (0.086H, m, *H*-C(2) of *exo* isomer), 2.10-1.31 (14H, m), [-CH_2-], 1.436 (8.496, s, -C(CH_3)$_3$ of *endo* isomer), 1.393 (0.504H, s, -C(CH_3)$_3$ of *exo* isomer). ^{13}C-NMR ($CDCl_3$): 178.25, 155.08, 139.10, 114.39, 80.33, 72.39, 71.75, 68.53, 60.56, 45.54, 36.19, 33.84, 33.31, 29.51, 28.83, 28.43, 25.72, 24.86. HRMS (ESI, [M-H]$^-$): Calcd. for $C_{20}H_{32}NO_5^-$: 366.2286; Found: 366.2321.

Synthesis of 27

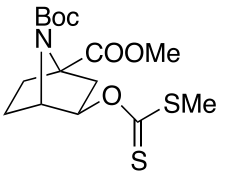

To a suspension of NaH (1.2955 g, 32.3878 mmol, ca. 60%) in THF (60 mL) was added CS_2 (14.61 mL, 242.907 mmol) and then **26** (2.1967 g, 8.0969 mmol) in THF (20 mL) at 0 °C. The reaction mixture was stirred for 2 h at 50 °C. To the reaction mixture was added MeI (10.08 mL, 161.938 mmol) at rt. The reaction mixture was stirred for 2 h at 40 °C. The reaction mixture was poured into aqueous solution of NH_4Cl (200 mL) and the whole was extracted with AcOEt (total 240 mL). The combined organic phase was dried over Na_2SO_4, and the solvent was evaporated. Column chromatography (n-hexane / AcOEt = 6 / 1) gave compound **27** (2.7683 g, 94%) as a yellow oil. ^{1}H-NMR ($CDCl_3$): 5.454-5.429 (1H, m, *H*-C(2)), 4.600 (1H, d, J = 5.6 Hz, bridgehead-*H*), 3.816 (3H, s, -OMe), 2.554 (3H, s, -SMe), 2.41-2.28 (2H, m), 2.22-2.10 (1H, m), 2.05-1.92 (1H, m), 1.72-1.62 (1H, m), 1.55-1.43 (1H, m), [-CH_2-], 1.436 (9H, s, -C(CH_3)$_3$). ^{13}C-NMR ($CDCl_3$): 215.55, 170.36, 155.72, 85.16, 81.36, 67.92, 63.20, 52.50, 41.75, 32.68, 28.26, 24.10, 19.40. HRMS (ESI, [M+Na]$^+$): Calcd. for $C_{15}H_{23}NNaO_5S_2^+$, 384.0910. Found: 384.0892. Anal. Calcd. for $C_{15}H_{23}NO_5S_2 \cdot 0.25H_2O$: C, 49.23; H, 6.47; N, 3.83. Found: C, 48.91; H, 6.15; N, 3.75.

Synthesis of 28

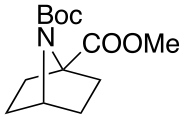

To a solution of **27** (2.6315 g, 7.2893 mmol) in toluene (120 mL) were added tris(trimethyl)silane (3.36 mL, 10.945 mmol) and AIBN (119.7 mg, 0.7289 mmol) at rt. The mixture was stirred reflux for 30 min and the solvent was evaporated to give a resiue. Column chromatography (n-hexane / AcOEt = 8 / 1) gave compound **28** (1.7575 g, 94%) as a white solid. M.p.: 34 - 39 °C. ^{1}H-NMR ($CDCl_3$): 4.306-4.282 (1H, m, bridgehead-*H*), 3.779 (3H, s, -OMe), 2.20-2.09 (2H, m), 1.98-1.85 (2H, m), 1.78-1.65 (2H, m), 1.52-1.42 (2H, m), [-C*H*$_2$-], 1.395 (9H, s, -C(C*H*$_3$)$_3$). ^{13}C-NMR ($CDCl_3$): 171.87, 156.61, 80.78, 68.83, 59.78, 52.22, 33.51, 29.39, 28.16. HRMS (ESI, $[M+Na]^+$): Calcd. for $C_{13}H_{21}NNaO_4^+$, 278.1363. Found: 278.1372. Anal. Calcd. for $C_{13}H_{21}NO_4 \cdot 0.2H_2O$: C, 60.31; H, 8.33; N, 5.41. Found: C, 60.65; H, 8.21; N, 5.30.

Synthesis of 29

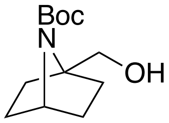

To a solution of **28** (1.7575 g, 6.884 mmol) in EtOH (15 mL) and THF (10 mL) were added $CaCl_2$ (powdered, 1.5282 g, 13.768 mmol) and $NaBH_4$ (1.0350 g, 27.536 mmol) at 0 °C. The mixture was warmed to room temperature and was stirred for 5 h at rt. The reaction mixture was poured into the cold saturated aqueous solution of NH_4Cl (100 mL), and the aqueous phase was extracted with AcOEt. The organic layer was dried over Na_2SO_4, and the solvent was evaporated. Column chromatography (n-hexane / AcOEt = 5.5 / 1) gave compound **29** (1.4430 g, 92%) as colorless needles. M.p.: 46.5 °C~47.5 °C, ^{1}H-NMR ($CDCl_3$): 4.903 (1H, brs, -OH), 4.248-4.224 (1H, m, bridgehead-*H*), 3.900 (2H, d, J=7.2 Hz, -C*H*$_2$OH), 1.92-1.70 (4H, m), 1.52-1.30 (4H, m), [-C*H*$_2$-], 1.445 (9H, s, -C(C*H*$_3$)$_3$). ^{13}C-NMR ($CDCl_3$): 80.27, 69.26, 62.15, 58.50, 31.96, 29.41, 28.52. Anal. Calcd. for $C_{12}H_{21}NO_3$: C, 63.41; H, 9.31; N, 6.16. Found: C, 63.11; H, 9.04; N, 6.05. HRMS (ESI, $[M+Na]^+$): Calcd. for $C_{12}H_{21}NNaO_3^+$, 250.1414. Found: 250.1409.

Synthesis of 30

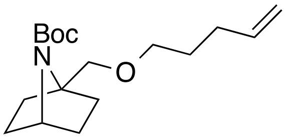

To a solution of **29** (58.5 mg, 0.2574 mmol) in DMF (1 mL) was added NaH (20.6 mg, 0.5148 mmol, ca. 60%) at 0 °C. The reaction mixture was stirred for 15 min at 0 °C. To the reaction mixture were added 5-bromo-1-pentene (120.9 μL, 1.0296 mmol) dropwise at 0 °C. The reaction mixture was stirred for 2 h at rt. Then another portion of NaH (20.6 mg, 0.5148 mmol, ca. 60%) and 5-bromo-1-pentene (120.9 μL, 1.0296 mmol) was added to the reaction mixture. After 1 h at rt, the third portion of NaH (20.6 mg, 0.5148 mmol, ca. 60%) and 5-bromo-1-pentene (120.9 μL, 1.0296 mmol) was added to the reaction mixture. The reaction mixture was stirred for 2 h at rt and was poured into cold water. The whole was extracted with AcOEt. The combined organic phase was dried over Na_2SO_4, and the solvent was evaporated. Column chromatography (n-hexane / AcOEt = 15 / 1) gave compound **30** (70.0 mg, 92%) as a colorless oil. ^{1}H-NMR ($CDCl_3$): 5.88-5.75 (1H, m), 5.05-4.91 (2H, m) [$CH=CH_2$], 4.256-4.233 (1H, m, bridgehead-*H*), 3.993 (2H, s, -CH_2-O-), 3.503 (3H, t, J=6.4 Hz, -OCH_2-), 2.137-2.077 (2H, m, -CH_2-CH=CH_2), 1.83-1.45 (8H, m), 1.50-1.30 (2H, m), [-CH_2-], 1.426 (9H, s, -C(CH_3)$_3$). ^{13}C-NMR ($CDCl_3$): 155.62, 138.61, 114.68, 79.49, 73.10, 70.99, 67.41, 58.87, 33.83, 30.45, 28.97, 28.50. HRMS (ESI, [M+Na]$^+$): Calcd. for $C_{17}H_{29}NNaO_3^+$, 318.2040. Found: 318.2044. Anal. Calcd. for $C_{17}H_{29}NO_3$: C, 69.12; H, 9.89; N, 4.74. Found: C, 69.07; H, 9.79; N, 4.62.

Synthesis of 31

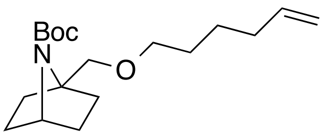

To a solution of **29** (300 mg, 1.320 mmol) in DMF (5 mL) was added NaH (105.6 mg, 0.5148 mmol, ca. 60%) at 0 °C. The reaction mixture was stirred for 15 min at 0 °C. To the reaction mixture was added 6-bromo-1-hexene (705.7 μL, 5.280 mmol) dropwise at 0 °C. The reaction mixture was stirred for 1.5 h at rt. Then another portion of NaH (79.2 mg, 1.98 mmol, ca. 60%) and 6-bromo-1-hexene (529 μL, 3.96 mmol) was added to the reaction mixture. After 1.5 h at rt, the third portion of NaH (52.8 mg, 1.32 mmol,

ca. 60%) and 6-bromo-1-hexene (352.9 μL, 2.64 mmol) was added to the reaction mixture. After 2 h at rt, the forth portion of NaH (52.8 mg, 1.32 mmol, ca. 60%) and 6-bromo-1-hexene (352.9 μL, 2.64 mmol) was added to the reaction mixture. The reaction mixture was stirred for 2 h at rt and was poured into cold water. The whole was extracted with AcOEt. The combined organic phase was dried over Na_2SO_4, and the solvent was evaporated. Column chromatography (n-hexane / AcOEt = 15 / 1) gave compound **31** (333.2 mg, 82%) as a colorless oil. ^{1}H-NMR ($CDCl_3$): 5.836-5.751 (1H, m), 5.017-4.915 (2H, m) [$CH=CH_2$], 4.261-4.238 (1H, m, bridgehead-*H*), 3.996 (2H, s, -C*H*$_2$-O-), 3.502 (3H, t, J=6.4 Hz, -OCH_2-), 2.093-2.032 (2H, m, -C*H*$_2$-$CH=CH_2$), 1.85-1.35 (12H, m), [-C*H*$_2$-], 1.432 (9H, s, -C(C*H*$_3$)$_3$). ^{13}C-NMR ($CDCl_3$): 155.64, 139.05, 114.51, 79.49, 73.12, 70.60, 67.44, 58.88, 33.86, 33.70, 29.24, 28.99, 28.52, 25.65. HRMS (ESI, [M+Na]$^+$): Calcd. for $C_{18}H_{31}NNaO_3^+$, 332.2196. Found: 332.2171.

Synthesis of 32

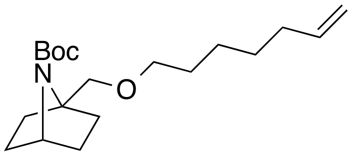

To a solution of **29** (45.0 mg, 0.1980 mmol) in DMF (2 mL) was added NaH (63.4 mg, 1.584 mmol, ca. 60%) at 0 ℃. The reaction mixture was stirred for 15 min at 0 °C. To the reaction mixture was added 6-bromo-1-hexene (211 μL, 1.386 mmol) dropwise at 0 °C. The reaction mixture was stirred for 1 h at 0 °C and 4.5 h at rt, and poured into cold water. The whole was extracted with AcOEt. The combined organic phase was washed with water and dried over Na_2SO_4, and the solvent was evaporated. Column chromatography (n-hexane / AcOEt = 15 / 1) gave compound **32** (56.8 mg, 82%) as a colorless oil. ^{1}H-NMR ($CDCl_3$): 5.834-5.732 (1H, m), 4.997-4.891 (2H, m) [$CH=CH_2$], 4.247-4.225 (1H, m, bridgehead-*H*), 3.979 (2H, s, -C*H*$_2$-O-), 3.478 (3H, t, J=6.4 Hz, -OCH_2-), 2.053-2.001 (2H, m, -C*H*$_2$-$CH=CH_2$), 1.83-1.28 (12H, m), [-C*H*$_2$-], 1.418 (9H, s, -C(C*H*$_3$)$_3$). ^{13}C-NMR ($CDCl_3$): 155.59, 139.11, 114.33, 79.43, 73.02, 71.68, 67.38, 58.82, 33.85, 33.79, 29.55, 28.93, 28.84, 28.46, 25.74. HRMS (ESI, [M+Na]$^+$): Calcd. for $C_{19}H_{33}NNaO_3^+$, 346.2353. Found: 346.2327.

Synthesis of 33

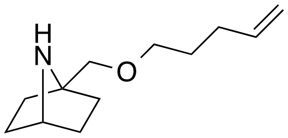

To a solution of **30** (348.6 mg) in CH_2Cl_2 (24 mL) was added TFA (4 mL) at rt. The mixture was stirred for 20 min at rt and evaporated. 10% Aqueous solution of sodium carbonate was added to the residue and the whole was extracted with CH_2Cl_2. The organic layer was dried over Na_2SO_4 and evaporated to give **33** as a pale yellow oil (240.7 mg, 100%), which was used without further purification.

Synthesis of 34

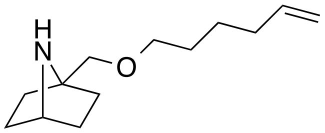

To a solution of **31** (183.5 mg) in CH_2Cl_2 (12 mL) was added TFA (2 mL) at rt. The mixture was stirred for 20 min at rt and evaporated. 10% Aqueous solution of sodium carbonate was added to the residue and the whole was extracted with CH_2Cl_2. The organic layer was dried over Na_2SO_4 and evaporated to give **34** as a pale yellow oil (128.3 mg, 100%), which was used without further purification.

Synthesis of 35

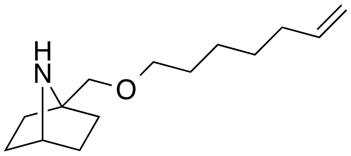

To a solution of **32** (49.8 mg) in CH_2Cl_2 (3 mL) was added TFA (0.5 mL) at rt. The mixture was stirred for 20 min at rt and evaporated. 10% Aqueous solution of sodium carbonate was added to the residue and the whole was extracted with CH_2Cl_2. The organic layer was dried over Na_2SO_4 and evaporated to give **35** as a pale yellow oil (34.4 mg, 100%), which was used without further purification.

Synthesis of 1

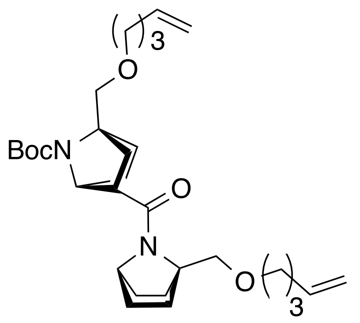

To a solution of **33** (109.2 mg, 0.559 mmol) and **23** (189.8 mg, 0.5592 mmol) in CH_2Cl_2 (15 mL) were added DMAP (20.4 mg, 0.1670 mmol) and a solution of EDCI·HCl (224.2 mg, 1.1695 mmol) in CH_2Cl_2 (5 mL) at 0 °C. The reaction mixture was stirred for 94 h at rt. The reaction mixture was washed with 5% aqueous solution of $KHSO_4$ and saturated aqueous solution of $NaHCO_3$, dried over Na_2SO_4 and evaporated. Column chromatography (n-hexane / AcOEt 5.7/1) gave compound **1** (226.6 mg, 78%) as s colorless oil. ^{1}H-NMR ($CDCl_3$): 5.851-5.743 (2H, m), 5.012-4.912 (4H, m) [CH=CH_2], 4.339 (1H, m), 4.311-4.288 (1H, m) [bridgehead-*H*], 4.206-4.143 (2H, m), 3.912-3.856 (2H, m) [-CH_2O-], 3.526-3.461 (4H, m, -OCH_2-), 3.099-3.072 (1H, m, *H*-C$_\alpha$-(C(=O)N)-), 2.129-2.063 (4H, m, -CH_2-CH= CH_2), 2.223-2.181 (1H, m), 1.83-1.36 (17H, m), [-CH_2-], 1.447 (9H, s, -C(CH_3)$_3$). ^{13}C-NMR ($CDCl_3$): 168.55, 155.61, 138.59, 138.56, 114.71, 114.66, 80.15, 73.15, 72.31, 70.93, 68.89, 68.10, 61.02, 58.15, 45.94, 36.25, 33.90, 33.40, 33.15, 30.43, 30.12, 29.94, 28.98, 28.94, 28.49, 23.81. HRMS (ESI, $[M+Na]^+$): Calcd. for $C_{30}H_{48}N_2NaO_5^+$; 539.3455, Found: 539.3462.

Synthesis of 2

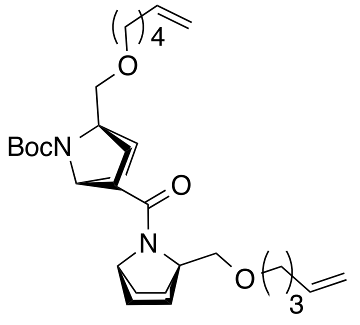

To a solution of **24** (208.5 mg, 0.5899 mmol) and **33** (115.2 mg, 0.5900 mmol) in CH_2Cl_2 (15 mL) were added DMAP (21.6 mg, 0.1768 mmol) and a solution of EDCI·HCl (226.2 mg, 1.1800 mmol) in CH_2Cl_2 (5 mL) at 0 °C. The reaction mixture

was stirred for 70 h at rt. The reaction mixture was washed with 5% aqueous solution of $KHSO_4$ and saturated aqueous solution of $NaHCO_3$, dried over Na_2SO_4 and evaporated. Column chromatography (n-hexane/AcOEt = 6/1 to 3/1) gave **2** (243.7 mg, 78%) as s colorless oil. ^{1}H-NMR ($CDCl_3$): 5.844-5.757 (2H, m), 5.022-4.916 (4H, m) [CH=CH_2 ×2], 4.346 (1H, m), 4.319-4.296 (1H, m) [bridgehead-*H*], 4.217-4.152 (2H, m), 3.916-3.861 (2H, m) [-CH_2O-], 3.535-3.463 (4H, m, -OCH_2-), 3.105-3.078 (1H, m, *H*-C_α-(C(=O)N)-), 2.126-2.024 (4H, m, -CH_2-CH=CH_2), 2.224-2.183 (1H, m), 1.83-1.36 (19H, m), [-CH_2-], 1.455 (9H, s, -C(CH_3)$_3$). ^{13}C-NMR ($CDCl_3$): 168.59, 155.65, 139.01, 138.62, 114.68, 114.54, 80.15, 73.19, 72.29, 71.55, 70.96, 68.93, 68.12, 61.04, 58.16, 45.97, 36.26, 33.93, 33.70, 33.42, 33.18, 30.45, 30.15, 29.97, 29.27, 28.97, 28.51, 25.63, 23.83. HRMS (ESI, [M+Na]$^+$): Calcd. for $C_{31}H_{50}N_2NaO_5^+$; 553.3612, Found: 553.3596.

Synthesis of 3

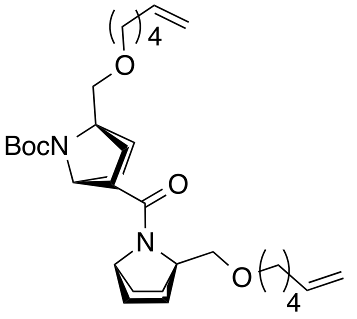

To a solution of **24** (208.5 mg, 0.5899 mmol) and **34** (124.1 mg, 0.5929 mmol) in CH_2Cl_2 (15 mL) were added DMAP (21.6 mg, 0.1768 mmol) and a solution of EDCI·HCl (226.2 mg, 1.1800 mmol) in CH_2Cl_2 (5 mL) at 0 °C. The mixture was stirred for 70 h at rt. The reaction mixture was washed with 5% aqueous solution of $KHSO_4$ and saturated aqueous solution of $NaHCO_3$, dried over Na_2SO_4 and evaporated. Column chromatography (n-hexane / AcOEt = 6/1 to 3/1) gave **3** (243.1 mg, 76%) as s colorless oil. ^{1}H-NMR ($CDCl_3$): 5.830-5.757 (2H, m), 5.010-4.911 (4H, m) [CH=CH_2], 4.345 (1H, m), 4.319-4.295 (1H, m) [bridgehead-*H*], 4.224-4.151 (2H, m), 3.917-3.861 (2H, m) [-CH_2O-×2], 3.527-3.463 (4H, m, -OCH_2-×2), 3.104-3.077 (1H, m, *H*-C_α-(C(=O)N)-), 2.081-2.024 (4H, m, -CH_2-CH=CH_2), 2.225-2.184 (1H, m), 1.85-1.36 (21H, m), [-CH_2-], 1.455 (9H, s, -C(CH_3)$_3$). ^{13}C-NMR ($CDCl_3$): 168.60, 155.65, 139.06, 139.01, 114.54, 114.50, 80.15, 73.18, 72.31, 71.56, 68.94, 68.14, 61.06, 58.17, 45.98, 36.28, 33.93, 33.70, 33.44, 33.19, 30.14, 29.97, 29.28, 29.22, 28.52, 25.63, 23.84. HRMS (ESI,

[M+Na]$^+$): Calcd. for $C_{32}H_{52}N_2NaO_5^+$; 567.3768, Found: 567.3741.

Synthesis of 4

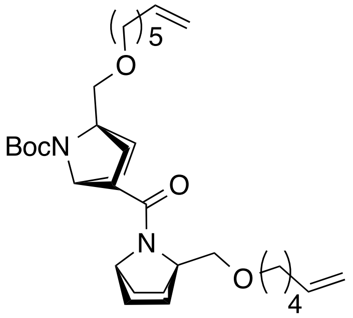

To a solution of **25** (174.9 mg, 0.4759 mmol) and **34** (109.6 mg, 0.524 mmol) in CH_2Cl_2 (10 mL) were added DMAP (17.0 mg, 0.139 mmol) and EDCI·HCl (182.5 mg, 0.952 mmol) at 0 °C, the reaction mixture was stirred for 48 h at rt. The reaction mixture was washed with 5% aqueous solution of $KHSO_4$ and saturated aqueous solution of $NaHCO_3$, dried over Na_2SO_4 and evaporated. Column chromatography (n-hexane / AcOEt = 8/1) gave compound **4** (139.4 mg, 52%) as a colorless oil.
^{1}H-NMR ($CDCl_3$): 5.817-5.716 (2H, m), 4.979-4.881 (4H, m) [CH=C*H*$_2$], 4.322 (1H, m), 4.294-4.271 (1H, m) [bridgehead-*H*], 4.189-4.125 (2H, m), 3.889-3.831 (2H, m) [-C*H*$_2$O-], 3.502-3.428 (4H, m, -OCH$_2$-), 3.083-3.057 (1H, m, *H*-C$_\alpha$-(C(=O)N)-), 2.054-1.982 (4H, m, -C*H*$_2$-CH=C*H*$_2$), 2.199-2.158 (1H, m), 1.90-1.20 (23H, m), [-C*H*$_2$-], 1.431 (9H, s, -C(C*H*$_3$)$_3$). ^{13}C-NMR ($CDCl_3$): 168.49, 155.57, 139.07, 138.95, 114.44, 114.31, 80.05, 73.08, 72.17, 71.60, 71.45, 68.86, 68.04, 60.95, 58.08, 45.89, 36.16, 33.84, 33.80, 33.62, 33.33, 33.08, 30.08, 29.89, 29.57, 29.13, 28.82, 28.44, 25.72, 25.53, 23.74. HRMS (ESI, [M+Na]$^+$): Calcd. for $C_{33}H_{54}N_2NaO_5^+$: 581.3925; Found: 581.3929. Anal. Calcd. for $C_{33}H_{54}N_2O_5 \cdot 0.2H_2O$: C, 70.48; H, 9.75; N, 4.98. Found: C, 70.17; H, 9.53; N, 5.08.

Synthesis of 5

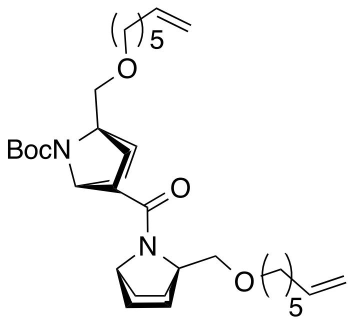

To a solution of **25** (52.2 mg, 0.1420 mmol) and **35** (34.4mg, 0.1539 mmol) in CH_2Cl_2 (5 mL) were added DMAP (5.2 mg, 0.0426 mmol) and EDCI·HCl (54.4 mg, 0.2840 mmol) at 0 °C. The mixture was stirred for 72 h at rt. The reaction mixture was diluted with CH_2Cl_2, washed with 5% aqueous solution of $KHSO_4$ and saturated aqueous solution of $NaHCO_3$. The organic layer was dried over Na_2SO_4, and evaporated. Column chromatography (n-hexane / AcOEt = 6/1) gave **5** (59.3 mg, 73%) as s colorless oil. ^{1}H-NMR ($CDCl_3$): 5.823-5.722 (2H, m), 4.986-4.878 (4H, m) [CH=CH_2 ×2], 4.327 (1H, m), 4.300-4.277 (1H, m) [bridgehead-*H*], 4.194-4.127 (2H, m), 3.894-3.837 (2H, m) [-CH_2O-], 3.498-3.433 (4H, m, -OCH_2-), 3.088-3.061 (1H, m, *H*-C_α-(C(=O)N)-), 2.040-1.987 (4H, m, -CH_2-CH=CH_2), 2.205-2.164 (1H, m), 1.85-1.24 (25H, m), [-CH_2-], 1.436 (9H, s, -C(CH_3)$_3$). ^{13}C-NMR ($CDCl_3$): 168.52, 155.59, 139.09, 114.31, 80.07, 73.08, 72.20, 71.62, 71.61, 68.88, 68.07, 60.98, 58.10, 45.91, 36.19, 33.86, 33.82, 33.35, 33.10, 30.09, 29.90, 29.59, 29.50, 28.83, 28.81, 28.45, 25.74, 25.70, 23.76. HRMS (ESI, $[M+Na]^+$): Calcd. for $C_{34}H_{56}N_2NaO_5^+$; 595.4081, Found: 595.4071.

Synthesis of 6

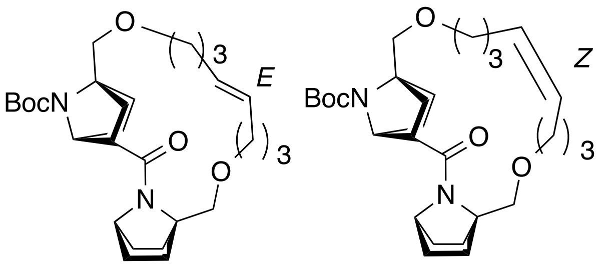

Ar was bubbled in a solution of **1** (72.1 mg, 0.1395 mmol) in 160 mL of CH_2Cl_2 for 1 h at rt. Then a solution of Grubbs 1st (8.1 mg, 9.842 μmol) in 10 mL of CH_2Cl_2 was added to the solution dropwise over 10 min at rt. The whole was heated at reflux for 8 h and the solvent was evaporated. Column chromatography (n-hexane/AcOEt=4.5/1) gave

6(*E)* (16.2 mg) as a colorless oil, **6**(*Z)* (18.4 mg) as colorless cubes and a mixture of them (15.2 mg, *E/Z*=39/62) (total yield: 73%).

6(*E)*: ^{1}H-NMR ($CDCl_3$, 60 °C): 5.651-5.470 (2H, m) [-CH=CH-], 4.502 (1H, m), 4.324-4.200 (1H, m) [bridgehead-*H*], 4.159-4.105 (2H, m), 3.940-3.828 (2H, m) [-C*H*$_2$O-], 3.585-3.543 (4H, m, -OCH_2-), 3.254-3.203 (1H, m, *H*-C_α-(C(=O)N)-), 2.29-1.37 (22H, m), [-C*H*$_2$-], 1.462 (9H, s, -C(C*H*$_3$)$_3$). ^{13}C-NMR ($CDCl_3$, 60 °C): 171.17, 155.25, 130.90, 130.70, 79.77, 72.71, 70.78, 70.15, 69.77, 69.28, 68.17, 62.00, 57.74, 44.07, 38.45, 33.84, 33.69, 32.52, 30.06, 29.94, 29.57, 28.90, 28.67, 28.56, 24.87. HRMS (ESI, $[M+Na]^+$): Calcd. for $C_{28}H_{44}N_2NaO_5^+$: 511.3142; Found: 511.3171.

6(*Z)*: (Recrystallized from CH_2Cl_2 / n-hexane) m.p. 117 °C-120 °C. ^{1}H-NMR ($CDCl_3$, 60 °C): 5.406-5.314 (2H, m) [-CH=CH-], 4.625 (1H, m), 4.317-4.226 (1H, m) [bridgehead-*H*], 4.239 (1H, d, J=10.4 Hz), 3.981-3.881 (2H, m), 3.675 (1H, d, J=10.4 Hz), [-C*H*$_2$O-], 3.575-3.487 (4H, m, -OCH_2-), 3.322-3.270 (1H, m, *H*-C_α-(C(=O)N)-), 2.35-1.37 (22H, m), [-C*H*$_2$-], 1.463 (9H, s, -C(C*H*$_3$)$_3$). ^{13}C-NMR ($CDCl_3$, 60 °C): 170.89, 154.96, 130.22, 130.08, 79.58, 72.12, 71.46, 70.32, 69.97, 69.32, 68.30, 62.18, 57.22, 43.27, 37.97, 35.19, 33.85, 32.47, 30.55, 30.51, 29.27, 28.68, 28.39, 25.38, 24.48, 24.14. HRMS (ESI, $[M+Na]^+$): Calcd. for $C_{28}H_{44}N_2NaO_5^+$: 511.3142; Found: 511.3154.

Synthesis of 7

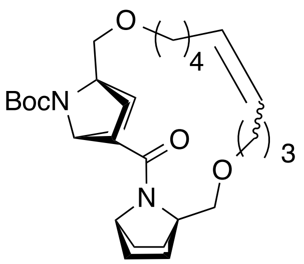

Ar was bubbled in a solution of **2** (146.7 mg, 0.2764 mmol) in 280 mL of CH_2Cl_2 for 1 h at rt. Then a solution of Grubbs 1st (11.4 mg, 13.82 μmol) in 10 mL of CH_2Cl_2 was added to the solution dropwise over 10 min at rt. The whole was heated at reflux for 4 h and the solvent was evaporated. Column chromatography (n-hexane/AcOEt=4.5/1) gave **7**(*E)* (59.6 mg, colorless oil) and **7**(*Z)* (28.7 mg, white solid) and their mixture (23.4 mg, E/Z=54/46) (total yield 80%).

7(*E)*: ^{1}H-NMR ($CDCl_3$, 60 °C): 5.619-5.437 (2H, m) [-CH=CH-], 4.441-4.418 (1H, m), 4.339-4.315 (1H, m) [bridgehead-*H*], 4.218 (1H, d, J=10.4 Hz), 4.120 (1H, d, J=10.8 Hz), 3.961 (1H, d, J=10.8 Hz), 3.885 (1H, d, J=10.8 Hz), [-C*H*$_2$O-], 3.583-3.491 (4H, m,

-OCH_2-), 3.178-3.127 (1H, m, *H*-$C_α$-(C(=O)N)-), 2.315-2.295 (1H,m), 2.14-1.34 (23H, m), [-C*H*$_2$-], 1.466 (9H, s, -C(C*H*$_3$)$_3$). ^{13}C-NMR ($CDCl_3$, 60 °C): 170.87, 155.53, 130.81, 130.35, 79.98, 73.69, 71.56, 70.04, 69.70, 69.21, 68.36, 61.80, 58.09, 44.97, 38.62, 34.14, 32.92, 32.32, 31.90, 30.28, 30.09, 29.16, 28.68, 28.66, 26.61, 24.90. HRMS (ESI, $[M+Na]^+$): Calcd. For $C_{29}H_{46}N_2NaO_5^+$: 525.3299, Found: 525.3290.

7(*Z)*: ^{1}H-NMR ($CDCl_3$, 60 °C): 5.375-5.277 (2H, m) [-CH=CH-], 4.545 (1H, m), 4.342-4.318 (1H, m) [bridgehead-*H*], 4.164 (1H, d, J=10.4 Hz), 4.021 (1H, d, J=10.4 Hz), 3.940 (1H, d, J=10.8 Hz), 3.756 (1H, d, J=10.4 Hz), [-C*H*$_2$O-], 3.580-3.530 (4H, m, -OCH_2-), 3.301-3.249 (1H, m, *H*-$C_α$-(C(=O)N)-), 2.26-1.33 (24H, m), [-C*H*$_2$-], 1.470 (9H, s, -C(C*H*$_3$)$_3$). ^{13}C-NMR ($CDCl_3$, 60 °C): 170.58, 155.22, 130.58, 130.10, 79.78, 72.65, 71.72, 71.65, 69.94, 69.15, 68.10, 62.04, 57.65, 44.15, 38.16, 34.11, 32.50, 30.59, 29.81, 29.50, 28.81, 28.66, 27.83, 27.69, 25.20, 24.63. HRMS (ESI, $[M+Na]^+$): Calcd. For $C_{29}H_{46}N_2NaO_5^+$: 525.3299, Found: 525.3282.

Synthesis of 8

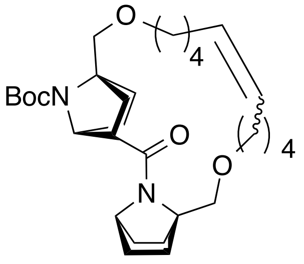

Ar was bubbled in a solution of **3** (103.7 mg, 0.1903 mmol) in CH_2Cl_2 (200 mL) for 1 h at rt. After that, Grubbs 1st (7.8 mg, 9.51 μmol) in 10 mL of CH_2Cl_2 was added to the solution dropwise over 10 min at rt. The whole was heated at reflux for 3 h, and evaporated. Column chromatography (n-hexane/AcOEt=3/1) gave **8** (92.7 mg, 94%, a mixture of *E*-alkene and *Z*-alkene) as a colorless oil.

A small amount of the mixture was seperated by high-performance liquid chromatography (HPLC) with a PEGASIL silica SP100 column (20 mmΦ x 250 mm) with 2-isopropanol/n-hexane (4:96) as an eluent to give **8**(*E)* (12.2 mg, colorless oil) and **8**(*Z)* (1.89 mg, colorless oil). **8**(*Z)* was not analyzed due to the small amont.

8(*E)*: HPLC retention time: t_R = 11.44 min. ^{1}H-NMR ($CDCl_3$, 55 °C): 5.531-5.431 (2H, m) [-CH=CH-], 4.410-4.388 (1H, m), 4.317-4.294 (1H, m) [bridgehead-*H*], 4.187 (1H, d, J=9.6 Hz), 4.035 (1H, d, J=10.0 Hz), 3.937 (1H, d, J=10.4 Hz), 3.880 (1H, d, J=10.0

Hz), [-CH_2O-], 3.592-3.455 (4H, m, -OCH$_2$-), 3.176-3.126 (1H, m, H-C$_\alpha$-(C(=O)N)-), 2.422-2.381 (1H,m), 2.14-1.34 (25H, m), [-CH_2-], 1.464 (9H, s, -C(CH_3)$_3$). ^{13}C-NMR (CDCl$_3$, 328.2 K): 169.72, 155.42, 130.74, 130.22, 80.06, 74.31, 71.32, 71.26, 70.00, 68.95, 67.94, 61.33, 58.11, 45.91, 38.38, 34.63, 32.62, 32.18, 31.59, 30.74, 29.67, 29.31, 29.03, 28.62, 27.04, 26.37, 24.39. HRMS (ESI, [M+Na]$^+$): Calcd. For $C_{30}H_{48}N_2NaO_5^+$: 539.3455, Found: 539.3462.

Synthesis of 9

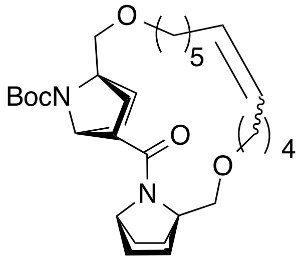

Ar was bubbled in a solution of **4** (113.9 mg, 0.2038 mmol) in CH$_2$Cl$_2$ (200 mL) for 1 h at rt. After that, Grubbs 1st (11.7 mg, μmol) in 3 mL of CH$_2$Cl$_2$ was added to the solution dropwise over 10 min at rt. The whole was heated at reflux for 2 h, and evaporated. Column chromatography (n-hexane/AcOEt=5/1 - 1/1) gave **9** (93.9 mg, 87%, a mixture of *E*-alkene and *Z*-alkene) as a colorless oil.

^{1}H-NMR (CDCl$_3$, 50 °C): 5.477-5.374 (1.60H, m) [-CH=CH-, *E*], 5.365-5.277 (0.40H, m) [-CH=CH-, *Z*], 4.371 (1H, br), 4.311-4.288 (1H, m) [bridgehead-*H*], 4.160 (0.20H, d, J=10.4 Hz), 4.10-3.95 (1.20H, br), 4.055 (0.80H, d, J=10.8 Hz), 3.994 (0.80H, d, J=9.6 Hz), 3.897 (0.20H, d, J=10.4 Hz), 3.837 (0.80H, d, J=9.6 Hz), [-CH_2O-], 3.564-3.460 (4H, m, -OCH$_2$-), 3.138-3.098 (1H, m, H-C$_\alpha$-(C(=O)N)-), 2.385-2.343 (1H, m), 2.14-1.00 (27H, m), [-CH_2-], 1.448 (9H, s, -C(CH_3)$_3$). ^{13}C-NMR (CDCl$_3$, 50 °C): 170.04, 169.58, 155.42, 130.90, 130.26, 129.98, 129.85, 79.95, 73.97, 71.83, 71.34, 70.91, 70.55, 69.69, 69.51, 68.87, 68.73, 68.29, 67.91, 61.26, 58.22, 46.09, 37.84, 37.53, 37.29, 34.54, 34.37, 32.86, 32.48, 32.23, 32.05, 31.69, 30.28, 30.10, 29.84, 29.81, 29.68, 29.61, 29.55, 29.41, 29.07, 28.55, 27.70, 27.23, 27.04, 26.83, 26.66, 26.32, 26.21, 24.43, 24.27, 22.78, 22.73, 14.12. HRMS (ESI, [M+H]$^+$): Calcd. For $C_{31}H_{51}N_2O_5^+$: 531.3792, Found: 531.3776.

Synthesis of 10

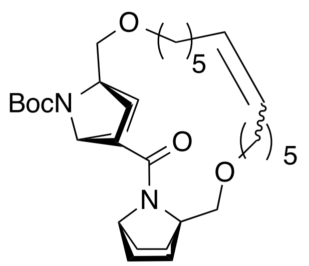

Ar was bubbled in a solution of **5** (48.0 mg, 0.08380 mmol) in CH_2Cl_2 (100 mL) for 1 h at rt. After that, Grubbs 1st (3.5 mg, 4.19 μmol) in CH_2Cl_2 (10 mL) was added to the solution dropwise at rt. The whole was heated at reflux for 16 h, and evaporated. Column chromatography (n-hexane/AcOEt = 4/1) gave **10** (a *E/Z* mixture, 29.0 mg, 64%) as white solid.

M.p. 95.0 °C - 98.0 °C.

^{1}H-NMR ($CDCl_3$, 50 °C): 5.473-5.287 (2H, m) [-CH=CH-], 4.352-4.305 (2H, m, bridgehead-*H*), 4.220 (d, J=10.4 Hz), 4.091 (d, J=9.6 Hz) (1H), 4.027-3.882 (3H, m) [-C*H*$_2$O-], 3.567-3.458 (4H, m, -OCH$_2$-), 3.146-3.078 (1H, m, *H*-C$_\alpha$-(C(=O)N)-), 2.420 (dd, J=12.0, 4.4 Hz), 2.365 (dd, J=12.0, 4.8 Hz) (1H), 2.055-1.355 (29H, m) [-C*H*$_2$-], 1.457 (9H, s, -C(C*H*$_3$)$_3$). ^{13}C-NMR ($CDCl_3$): 169.05, 155.46, 79.97, 73.31, 71.20, 70.48, 69.56, 68.59, 67.95, 60.88, 58.16, 46.29, 36.75, 33.90, 31.94, 31.67, 29.82, 29.76, 29.69, 29.47, 29.09, 28.95, 28.82, 28.63, 28.59, 28.36, 27.94, 25.88, 25.74, 23.90.

HRMS (ESI, [M+Na]$^+$): Calcd. For $C_{32}H_{52}N_2NaO_5^+$: 567.3768, Found: 567.3745.

Synthesis of 11

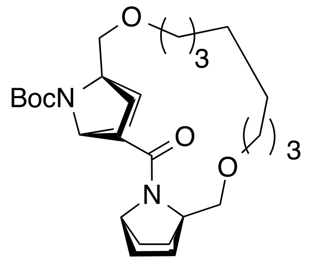

To a solution of **6** (14.4 mg, 0.0295 mmol, *E/Z* mixture) in MeOH (1 mL) was added a suspension of 10% Pd/C (4 mg) in MeOH (0.5 mL). The whole was stirred under H_2 atmosphere for 2 h at rt and filterated. The filtrate was evaporated to give **11** as colorless oil (12.9 mg, 89%), which was used without further purification.

^{1}H-NMR ($CDCl_3$, 55 °C): 4.504 (1H, m, bridgehead-*H*), 4.311-4.288 (1H, m, bridgehead-*H*), 4.103-4.076 (2H, m), 3.830 (2H, m) [-C*H*$_2$O-], 3.619-3.575 (1H, m, *H*-C$_\alpha$-(C(=O)N)-), 3.545-3.496 (3H, m), 3.268-3.229 (1H, m) [-OCH$_2$-], 2.144-1.278

(26H, m, -CH_2-), 1.465 (9H, s, -C(CH_3)$_3$). ^{13}C-NMR (CDCl$_3$, 55 °C): 170.85, 155.14, 79.76, 72.58, 71.40, 70.97, 69.89, 69.16, 67.92, 61.83, 57.73, 44.04, 38.77, 33.97, 32.27, 30.19, 29.62, 29.55, 29.14, 28.67, 26.55, 26.06, 24.69, 22.79. HRMS (ESI, [M+Na]$^+$): Calcd. For $C_{28}H_{46}N_2NaO_5^+$: 513.3299, Found: 513.3305.

Synthesis of 12

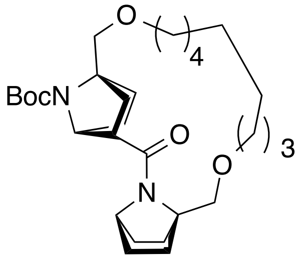

To a solution of **7** (23.4 mg, 0.04655 mmol) in MeOH (1 mL) was added a suspension of 10% Pd/C (4.0 mg) in MeOH (1 mL). The whole was stirred under H_2 atmosphere for 80 min at rt, and filtered. The filtrate was evaporated. Column chromatography (n-hexane/AcOEt =4.5/1) gave **12** (20.6 mg, 88%) as a colorless oil. ^{1}H-NMR (CDCl$_3$, 50 °C): 4.441 (1H, m), 4.331-4.308 (1H, m) [bridgehead-*H*], 4.178 (1H, br), 4.074 (1H, d, J=10.4 Hz), 3.886 (1H, d, J=10.4 Hz), 3.848-3.824 (1H, m), [-CH_2O-], 3.577-3.465 (4H, m, -OCH$_2$-), 3.211-3.184 (1H, m, *H*-C$_\alpha$-(C(=O)N)-), 2.290-2.268 (1H, m), 2.09-1.20 (27H, m), [-CH_2-], 1.457 (9H, s, -C(CH_3)$_3$). ^{13}C-NMR (CDCl$_3$, 50 °C): 170.61, 155.30, 79.92, 73.65, 71.94, 71.08, 69.59, 68.89, 68.08, 61.54, 58.06, 44.90, 38.58, 34.31, 33.12, 32.16, 30.20, 29.84, 29.66, 29.27, 29.24, 28.99, 28.60, 27.51, 26.53, 24.72. HRMS (ESI, [M+Na]$^+$): Calcd. For $C_{29}H_{48}N_2NaO_5^+$: 527.3455, Found: 527.3461.

Synthesis of 13

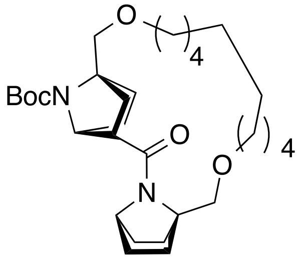

To a solution of **8** (10.0 mg, 0.01935 mmol, *E/Z*=89/11) in MeOH (1 mL) was added a suspension of 10% Pd/C (3.8 mg) in MeOH (0.5 mL). The whole was stirred under H_2 atmosphere for 2 h at rt and filtrated. The filtrate was evaporated to give **13** (9.8 mg, 98%) as a colorless oil. ^{1}H-NMR (CDCl$_3$, 50 °C): 4.375-4.351 (1H, m), 4.332-4.309

(1H, m) [bridgehead-*H*], 4.255 (1H, d, J=10.0 Hz), 4.013 (1H, d, J=10.0 Hz), 3.931 (1H, d, J=10.0 Hz), 3.889 (1H, d, J=9.6 Hz), [-CH_2O-], 3.588-3.458 (4H, m, -OCH_2-), 3.160-3.109 (1H, m, *H*-C_α-(C(=O)N)-), 2.463-2.421 (1H, m), 2.09-1.20 (29H, m), [-CH_2-], 1.463 (9H, s, -C(CH_3)$_3$). ^{13}C-NMR ($CDCl_3$, 50 °C): 169.86, 155.49, 80.13, 74.55, 71.67, 71.11, 69.80, 68.77, 68.08, 61.23, 58.28, 46.20, 38.32, 34.61, 32.24, 32.02, 30.88, 29.70, 29.67, 29.53, 29.46, 29.40, 29.37, 28.91, 28.60, 26.46, 26.32, 24.43. HRMS (ESI, [M+Na]$^+$): Calcd. For $C_{30}H_{50}N_2NaO_5^+$: 541.3612, Found: 541.3606.

Synthesis of 14

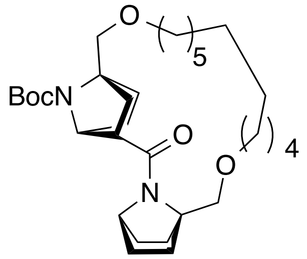

To a solution of **9** (77.9 mg, 0.147 mmol, *E/Z* mixture) in MeOH (3.0 mL) was added a suspension of 10% Pd/C (11.3 mg) in MeOH (1.0 mL). The whole was stirred under H_2 atmosphere for 3 h at rt and filtered. The filtrate was evaporated. Column chromatography (n-hexane/AcOEt = 6/1) gave compound **14** (60.7 mg, 78%) as a colorless oil.

^{1}H-NMR ($CDCl_3$, 50 °C): 4.366 (1H, t, J=4.8 Hz, bridgehead-*H*), 4.319 (1H, t, J=4.4 Hz, bridgehead-*H*), 4.128 (1H, d, J=10.4 Hz), 3.995 (2H, dd, J=1.2 Hz, 10.4 Hz), 3.892 (1H, d, J=9.6 Hz) [-CH_2O-], 3.547-3.477 (4H, m, -OCH_2-), 3.128, (1H, m, *H*-C_α-(C(=O)N)-), 2.422 (1H, dd, J=4.4 Hz, 12.0 Hz), 1.976-1.272 (40H, m) [-CH_2-]. ^{13}C-NMR ($CDCl_3$, 50 °C): 169.62, 155.50, 80.07, 74.27, 71.83, 71.06, 69.71, 68.76, 68.10, 61.24, 58.38, 46.42, 37.64, 34.59, 32.21, 30.43, 30.23, 30.05, 30.00, 29.78, 29.72, 29.40, 29.22, 28.84, 28.58, 26.90, 26.37, 24.29.

HRMS (ESI, [M+H]$^+$): Calcd. For $C_{31}H_{53}N_2O_5^+$: 533.3949, Found: 533.3975.

Compound 15

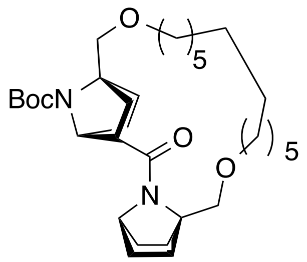

To a solution of **10** (26.8 mg, 0.04923 mmol, *E/Z* mixture) in MeOH (1.5 mL) was added a suspension of 10% Pd/C (4.0 mg) in 0.5 mL of MeOH. The whole was stirred under H_2 atmosphere for 3 h at rt and filtered. The filtrate was evaporated. Column chromatography (n-hexane/AcOEt = 4/1) gave **15** (24.4 mg, 91%) as a colorless oil. ^{1}H-NMR ($CDCl_3$): 4.340 (1H, m), 4.315-4.292 (1H, m), [bridgehead-*H*], 4.158 (1H, d, J=10.0 Hz), 4.000 (1H, d, J=10.0 Hz), 3.902 (2H, brs), [-CH_2O-], 3.535-3.478 (4H, m, -OCH_2-), 3.139-3.114 (1H, m, *H*-C_α-(C(=O)N)-), 2.405-2.364 (1H, m), 2.02-1.90 (1H, m), 1.90-1.17 (32H, m) [-CH_2-], 1.448 (9H, s, -C(CH_3)$_3$). ^{13}C-NMR ($CDCl_3$): 169.17, 155.59, 80.11, 73.43, 71.34, 70.61, 69.69, 68.72, 68.08, 61.01, 58.29, 46.41, 36.88, 34.03, 32.07, 31.80, 29.96, 29.89, 29.82, 29.60, 29.22, 29.08, 28.95, 28.76, 28.72, 28.49, 28.07, 26.01, 25.87, 24.03. HRMS (ESI, $[M+Na]^+$): Calcd. For $C_{32}H_{54}N_2NaO_5^+$: 569.3925, Found: 569.3943.

Synthesis of 6(*Z*)-NH

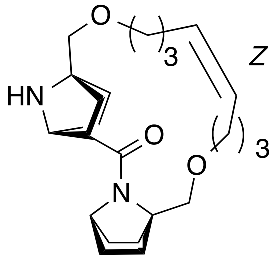

To a solution of **6**(*Z*) (26.0 mg, 0.05321 mmol) in CH_2Cl_2 (1.2 mL) was added TFA (0.2 mL) and the whole was stirred for 30 min at rt. After that, TFA (0.2 mL) was added to the reaction mixture and the whole was stirred for 20 min at rt and evaporated. 5% Aqueous solution of $KHSO_4$ was added to the residue and the whole was washed with AcOEt. The aqueous layer was basified to pH=10 by adding 10% aqueous solution of Na_2CO_3 and extracted with chloroform. The organic layer was dried over Na_2SO_4 and evaporated. Column chromatography ($CHCl_3$/MeOH=15/1) and preparative TLC ($CHCl_3$/MeOH=15/1) gave **6(*Z*)-NH** (6.9 mg, 33%) as a colorless oil. ^{1}H-NMR ($CDCl_3$): 5.449-5.364 (2H, m) [-CH=CH-], 4.805-4.780 (1H, m), 4.008 (1H, d, J=11.6

Hz), 3.84-3.78 (1H, m), 3.747-3.394 (8H, m), 2.40-1.18 (22H, m). ^{13}C-NMR ($CDCl_3$): 169.47, 130.28, 129.50, 72.27, 71.39, 70.29, 70.14, 70.08, 68.29, 61.22, 56.50, 46.78, 36.14, 34.78, 32.90, 31.72, 29.96, 29.83, 27.94, 25.22, 24.55, 23.80. HRMS (ESI, $[M+Na]^+$): Calcd. For $C_{23}H_{37}N_2O_3^+$: 389.2799, Found: 389.2810.

Synthesis of 6(*E)*-NH

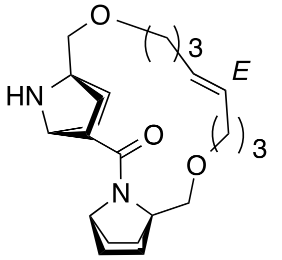

To a solution of **6**(*E)* (26.8 mg, 0.05484 mmol) in CH_2Cl_2 (1.2 mL) was treated with 0.4 mL of TFA and the whole was stirred for 30 min at 0 °C and 30 min at rt. The solvent was evaporate before adding 10% aqueous solution of Na_2CO_3 to adjust the pH to 10. The aqueous layer was extracted with CH_2Cl_2, dried over Na_2SO_4. Then the solvent was evaporated. Column chromatography ($CHCl_3$/MeOH=15/1) and preparative TLC ($CHCl_3$/MeOH=15/1) gave compound **6**(*E)*-**NH** (3.1 mg, 15%) as white amorphous solid. ^{1}H-NMR ($CDCl_3$): 5.595-5.496 (2H, m), 4.744 (1H, m), 3.997 (1H, d, J=11.6 Hz), 3.84-3.78 (1H, m), 3.78-3.48 (7H, m), 3.455-3.405 (1H, m), 2.30-1.38 (22H, m). ^{13}C-NMR ($CDCl_3$): 169.20, 131.86, 128.95, 72.50, 71.82, 70.86, 69.69, 68.21, 61.23, 56.94, 46.26, 34.92, 34.31, 33.60, 31.19, 30.73, 29.85, 29.22, 28.98, 28.45, 27.89, 25.06. HRMS (ESI, $[M+Na]^+$): Calcd. For $C_{23}H_{37}N_2O_3^+$: 389.2799, Found: 389.2797.

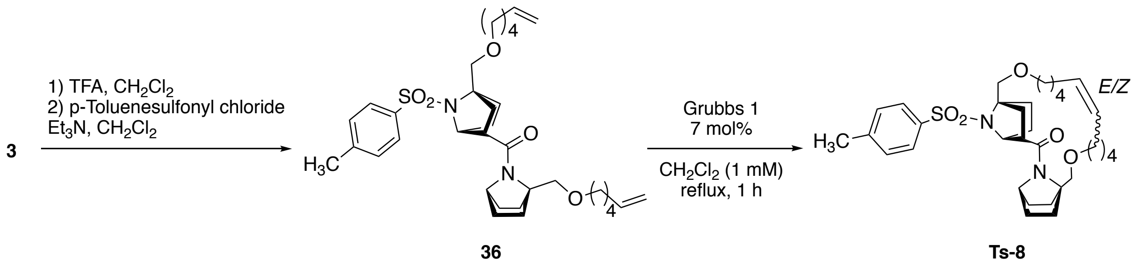

Compound 36

To a solution of **3** (20.4 mg, 0.03734 mmol) in CH_2Cl_2 (0.9 mL) was added TFA (0.15 mL) and the whole was stirred for 5 h at 0 °C and evaporated. 10% Aqueous solution of Na_2CO_3 was added to the residue to adjust the pH to 10. The whole was extracted with CH_2Cl_2 and the combined organic layer was dried over Na_2SO_4 and evaporated to give the crude amine (70.6 mg, 100%) as colorless oil. The crude amine (61.6 mg) was

dissolved in CH_2Cl_2 and Et_3N (38.6 mL, 0.2770 mmol) and *p*-toluenesulfonyl chloride (39.6 mg, 0.1385 mmol) were added to the solution at 0 °C. The mixture was stirred for 15 h at rt. The mixture was washed with 5% aqueous solution of $KHSO_4$, dried over Na_2SO_4 and evaporated. Column chromatography (n-hexane/AcOEt = 4/1) gave compound **36** (77.4 mg, 93%) as colorless oil.

^{1}H-NMR ($CDCl_3$): 7.835 (2H, d, J=8.2 Hz), 7.256 (2H, d, J=8.2 Hz), 5.842-5.710 (2H, m), 5.008-4.994 (1H, m), 4.965-4.921 (2H, m), 4.915-4.908 (1H, m), 4.406-4.386 (1H, m), 4.337 (1H, m), 4.203-4.130 (2H, m), 3.755 (1H, d, J=10.6 Hz), 3.691 (1H, d, J=10.6 Hz), 3.511 (1H, d, J=6.4 Hz), 3.495 (1H, d, J=6.4 Hz), 3.235-3.139 (3H, m), 2.410 (3H, s), 2.141 (1H, dd, J=12.0, 4.8 Hz), 2.077-1.313 (25H, m).

^{13}C-NMR ($CDCl_3$): 167.88, 143.39, 139.03, 138.99, 138.88, 129.35, 127.75, 114.60, 114.50, 72.98, 71.54, 71.31, 69.96, 68.28, 63.66, 58.28, 47.41, 35.84, 33.88, 33.70, 33.68, 33.17, 33.06, 30.01, 29.20, 29.06, 25.60, 25.50, 24.61, 21.65.

HRMS (ESI, $[M+Na]^+$): Calcd. For $C_{34}H_{50}N_2NaO_5S^+$: 621.3333, Found: 621.3324.

Ts-8 (a mixture of *E*-alkene and *Z*-alkene)

Ar was bubbled in a solution of **36** (77.0 mg, 0.1286 mmol) in CH_2Cl_2 (150 mL) for 1 h at rt. After that, Grubbs 1st (3.2 mg, 3.888 μmol) in 10 mL of CH_2Cl_2 was added to the solution dropwise over 15 min at rt. The whole was heated at reflux for 1 h, and evaporated. Column chromatography (n-hexane/AcOEt=5/2 - 3/2) gave **Ts-8** (66.9 mg, 91%, a mixture of *E*-alkene and *Z*-alkene) as white solid.

M.p.: 121.5 °C - 124.5 °C (colorless cubes, recrystallized from methanol).

^{1}H-NMR ($CDCl_3$, 60 °C): 7.801 (2H, d, J=8.2 Hz), 7.258 (2H, d, J=8.2 Hz), 5.497-5.337 (2H, m), 4.486 (m), 4.402 (m) (1H), 4.340-4.329 (1H, m), 4.067-3.869 (3H, m), 3.681 (d, J=10.4 Hz), 3.570 (d, J=10.4 Hz) (1H), 3.528-3.347 (4H, m), 3.267-3.240 (1H, m), 2.418 (3H, s), 2.311 (1H, m), 2.100-1.380 (25H, m).

^{13}C-NMR ($CDCl_3$, 60 °C): 168.94, 143.38, 139.69, 130.68, 130.21, 130.08, 129.52, 129.46, 127.72, 72.96, 72.21, 71.41, 71.29, 69.92, 69.85, 68.43, 68.09, 64.86, 64.40, 57.96, 57.69, 46.30, 38.04, 34.49, 34.25, 33.05, 32.56, 32.33, 31.70, 31.45, 30.30, 30.05, 29.67, 29.20, 28.84, 27.64, 27.56, 27.36, 26.99, 26.73, 26.37, 25.62, 25.37, 22.74, 21.55.

HRMS (ESI, $[M+Na]^+$): Calcd. For $C_{32}H_{46}N_2NaO_5S^+$: 593.3020, Found: 593.3039. Anal. Calcd. for $C_{32}H_{46}N_2O_5S$: C, 67.34; H, 8.12; N, 4.91. Found: C, 67.02; H, 8.21; N, 4.88.

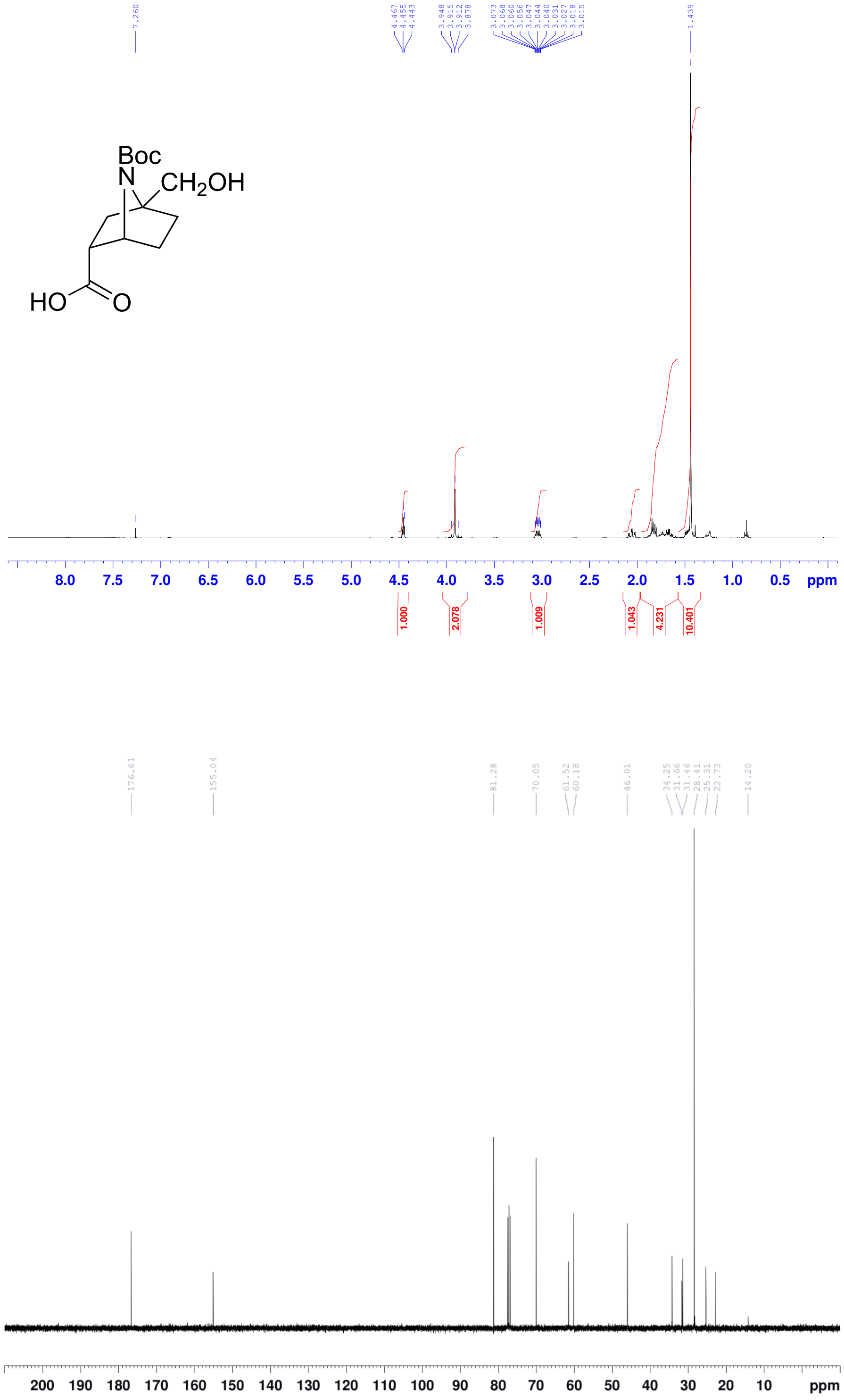

Supplementary Figure 19. ^{1}H and ^{13}C-NMR Charts of Compound 22

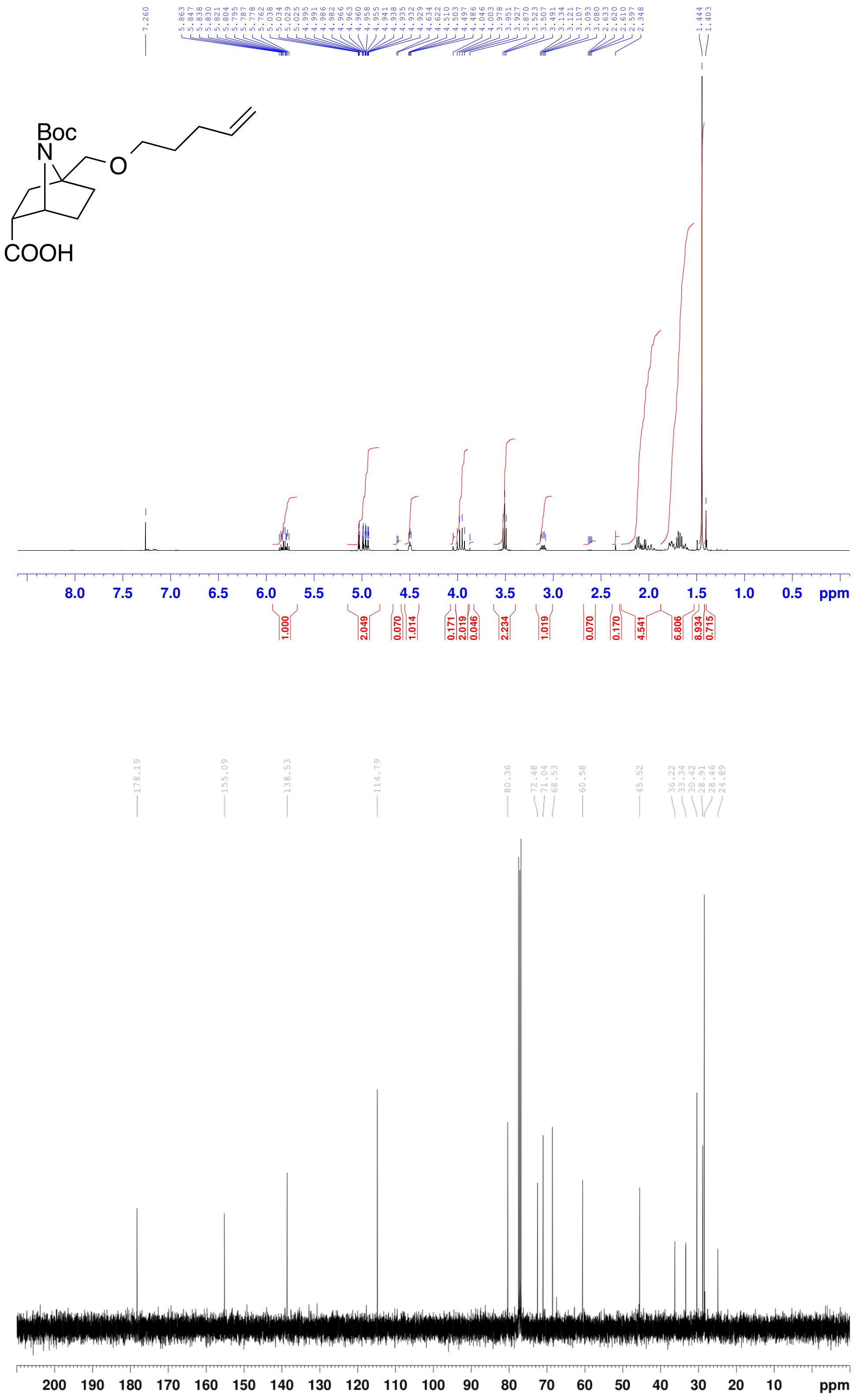

Supplementary Figure 20. ^{1}H and ^{13}C-NMR Charts of Compound 23

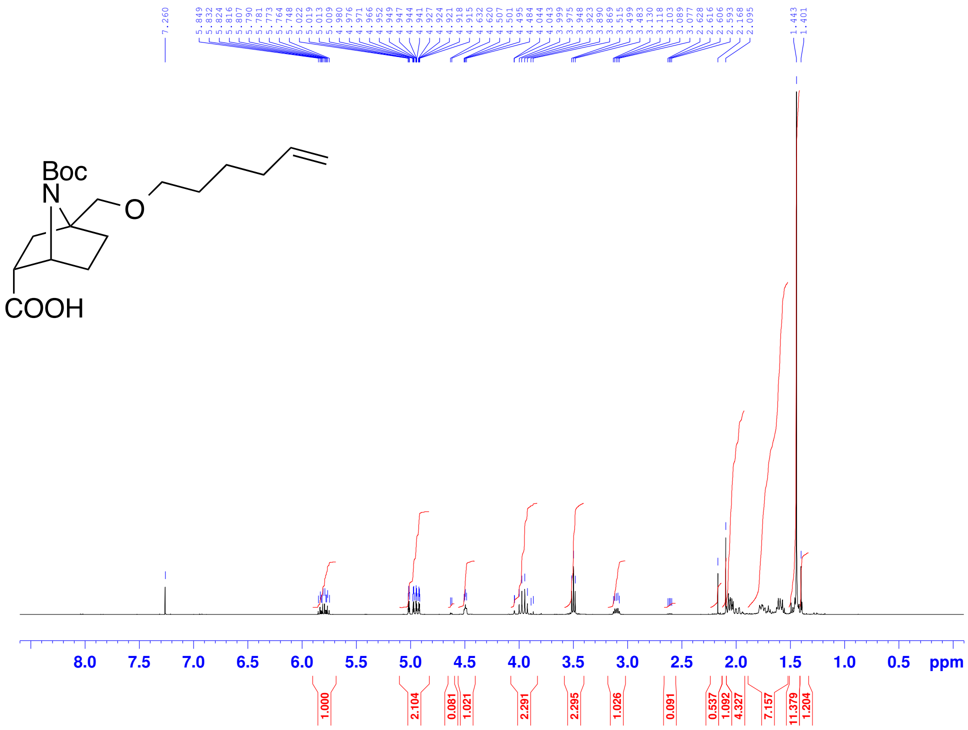

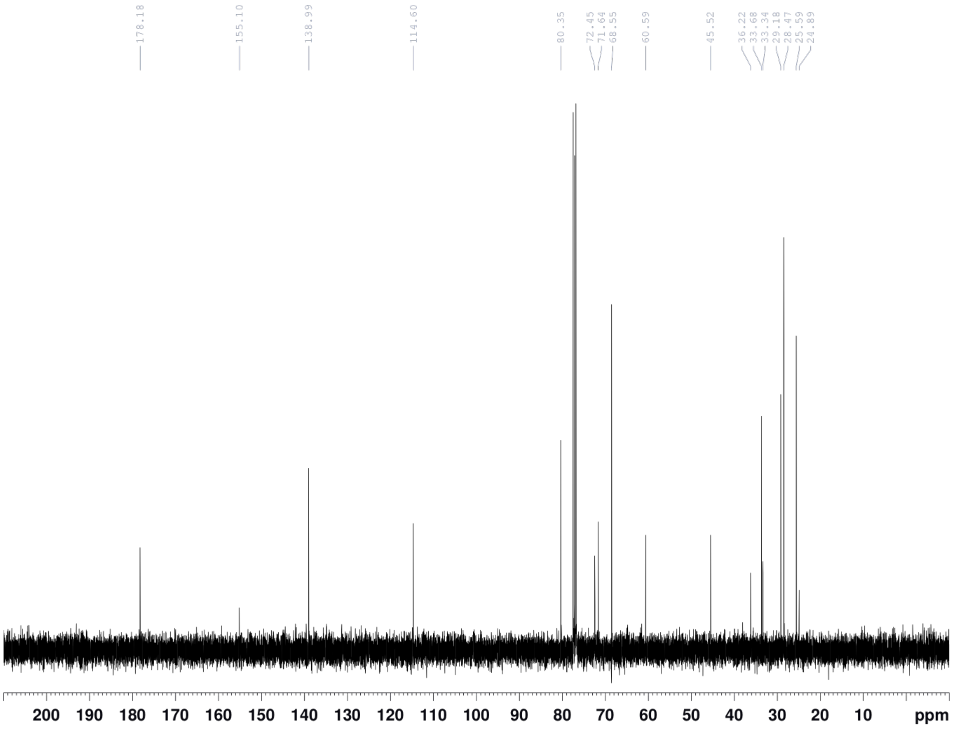

Supplementary Figure 21. ^{1}H and ^{13}C-NMR Charts of Compound 24

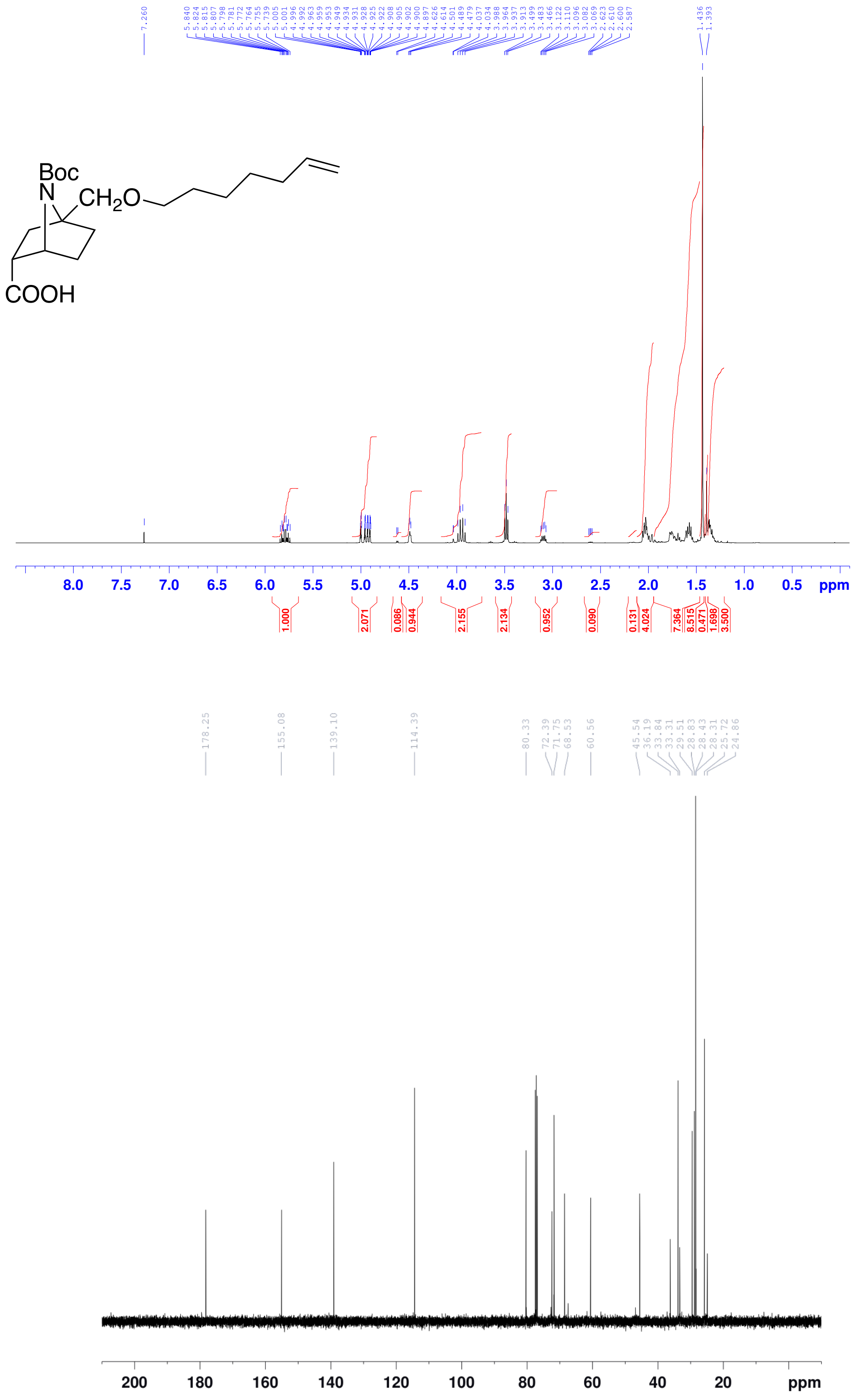

Supplementary Figure 22. ^{1}H and ^{13}C-NMR Charts of Compound 25

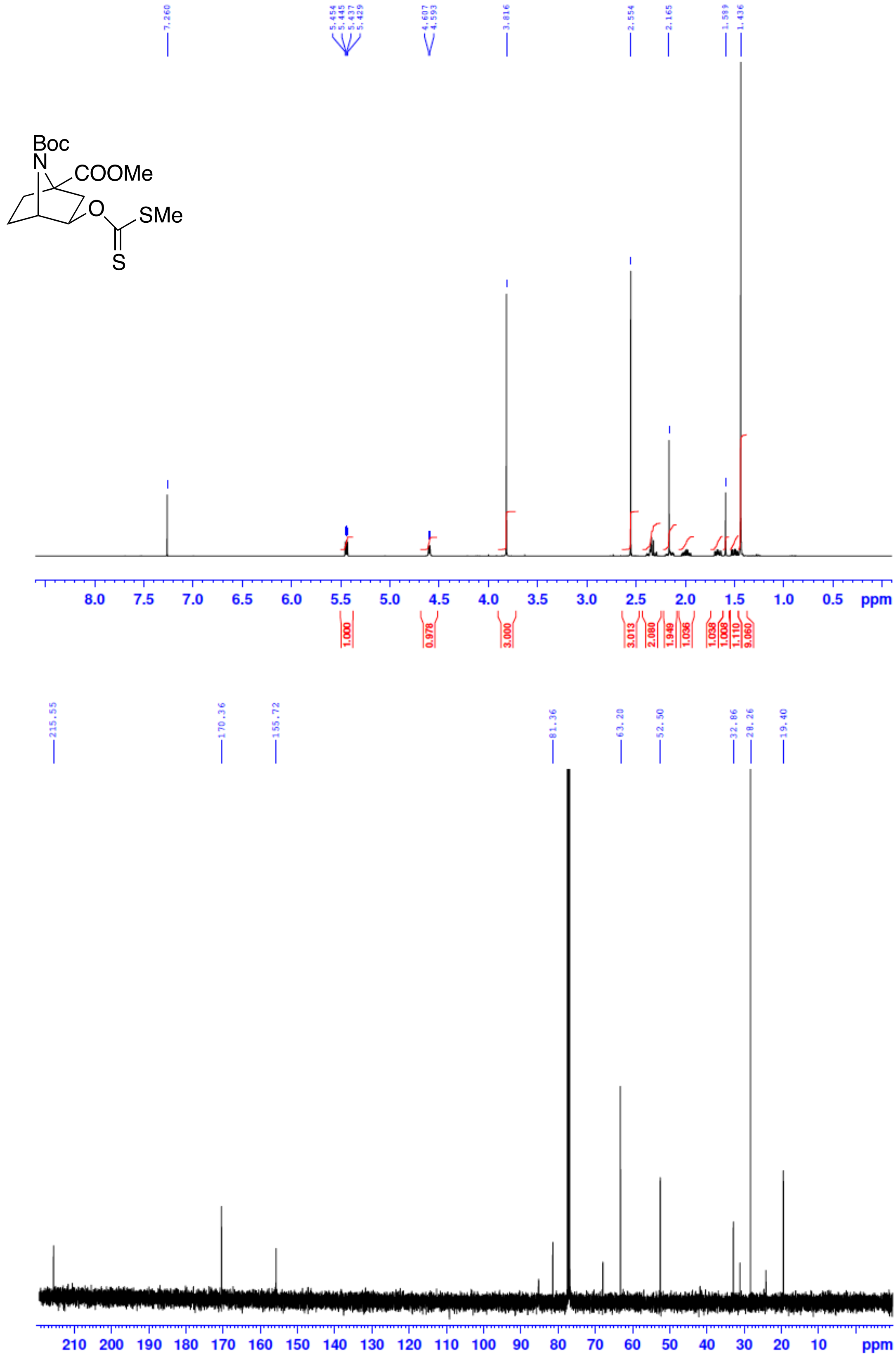

Supplementary Figure 23. ^{1}H and ^{13}C-NMR Charts of Compound 27

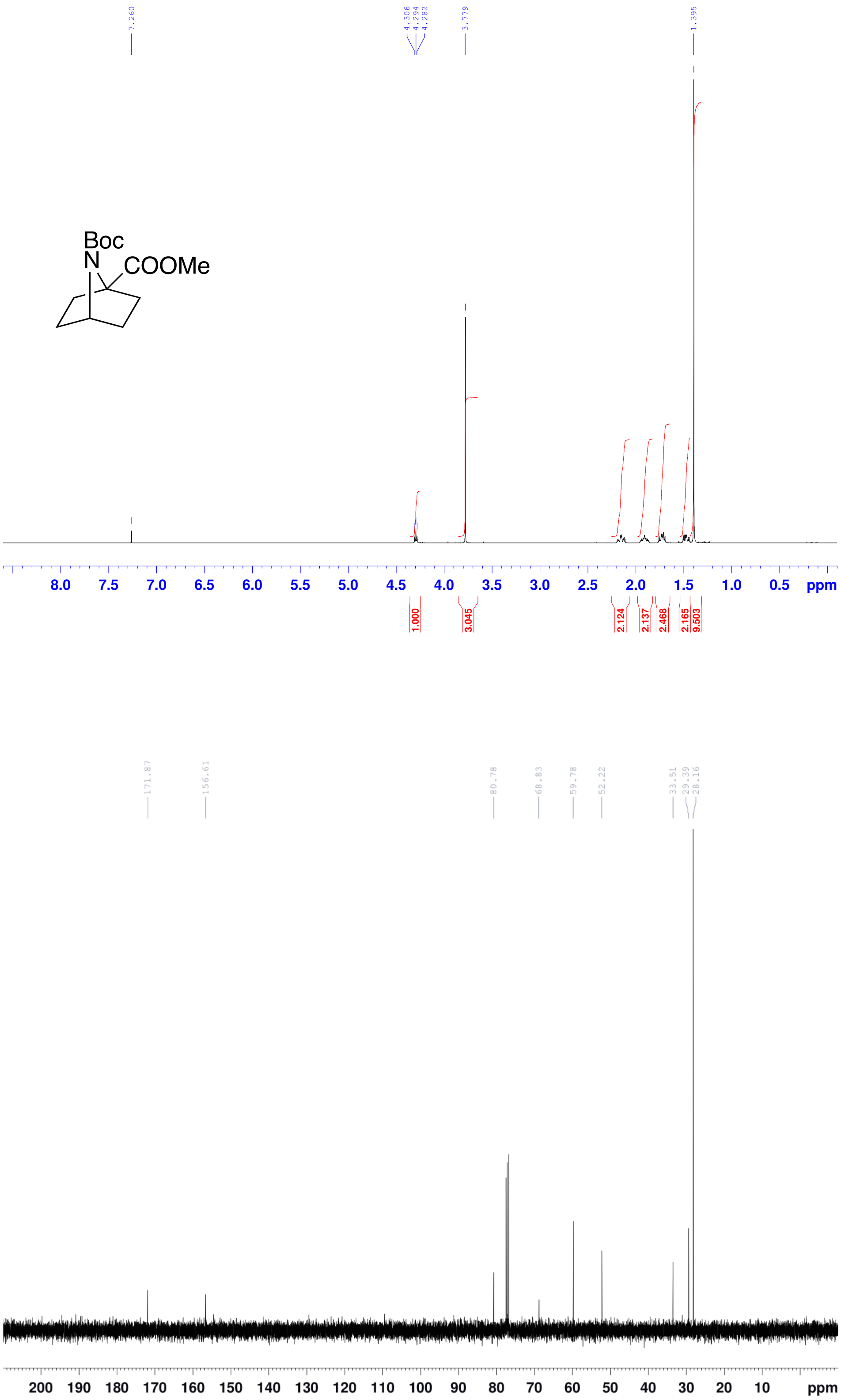

Supplementary Figure 24. ^{1}H and ^{13}C-NMR Charts of Compound 28

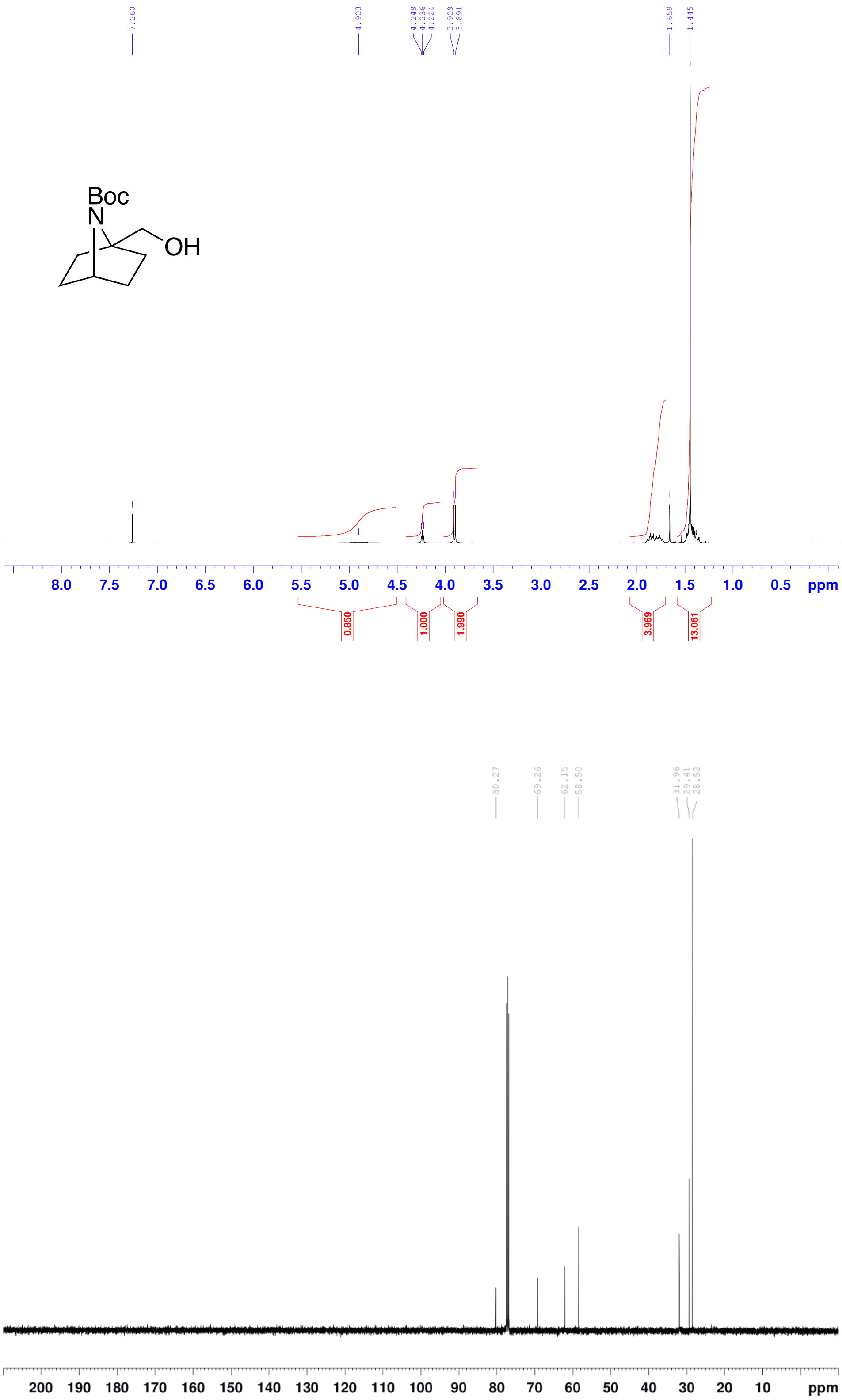

Supplementary Figure 25. ^{1}H and ^{13}C-NMR Charts of Compound 29

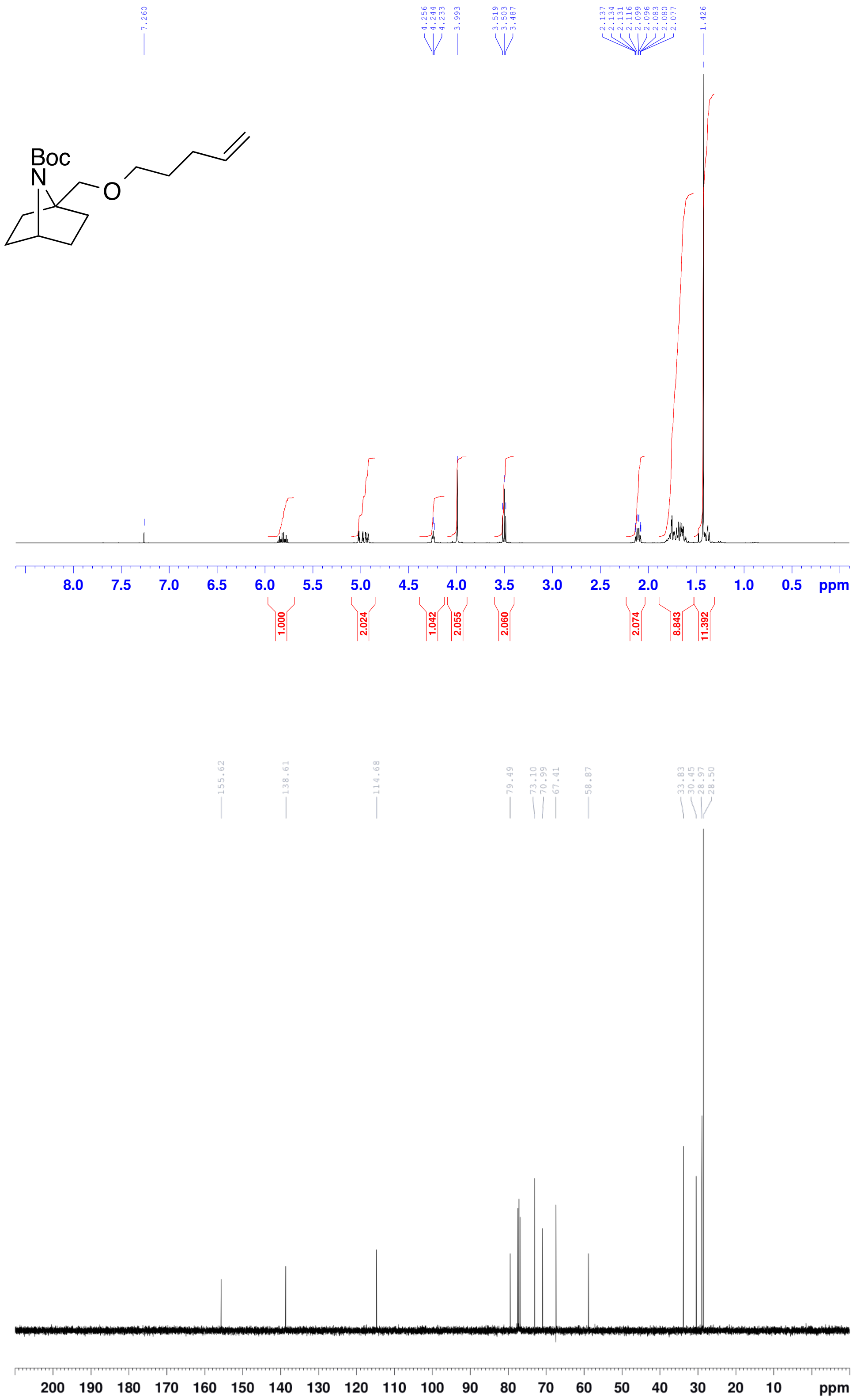

Supplementary Figure 26. ^{1}H and ^{13}C-NMR Charts of Compound 30

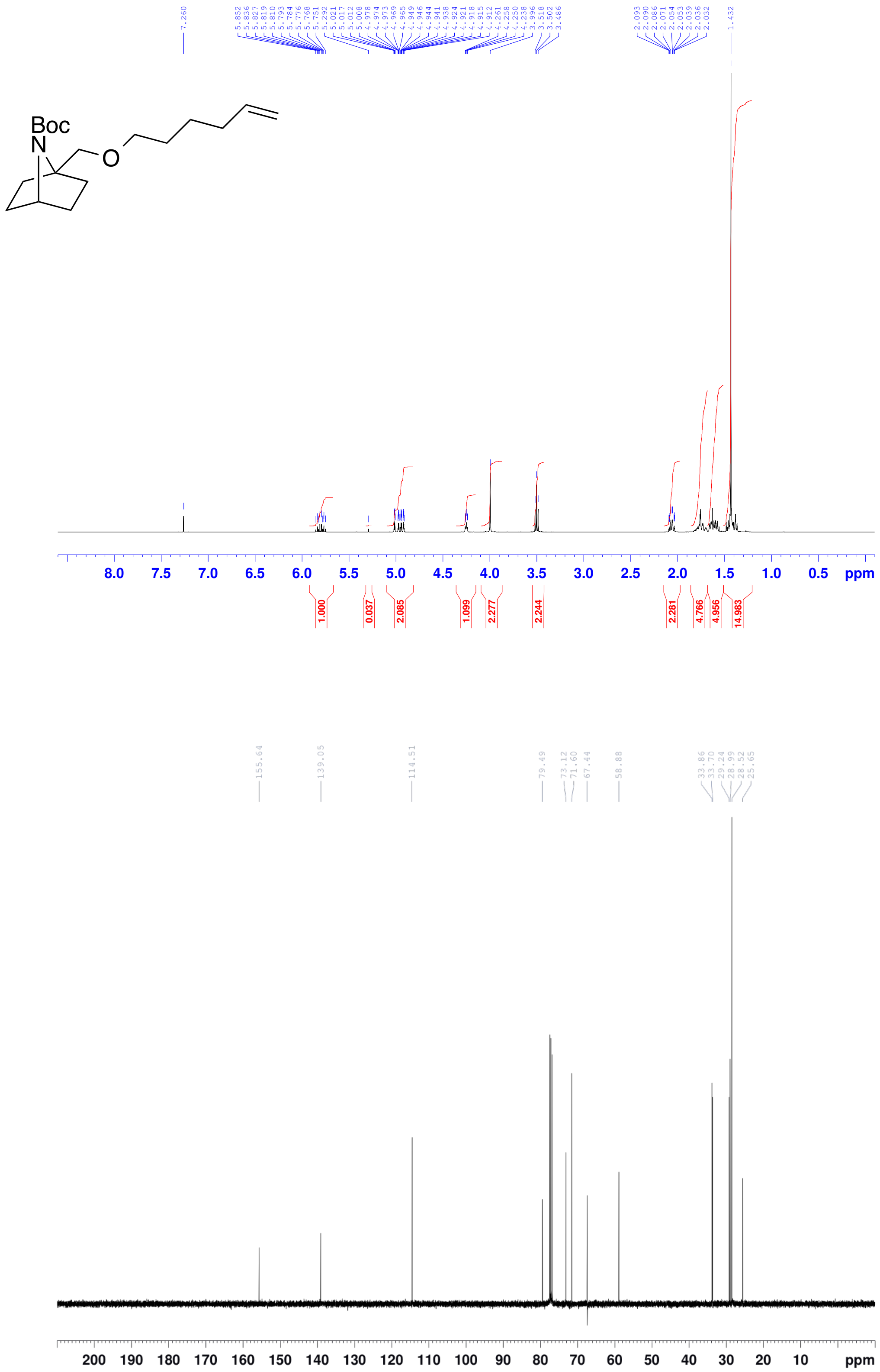

Supplementary Figure 27. ^{1}H and ^{13}C-NMR Charts of Compound 31

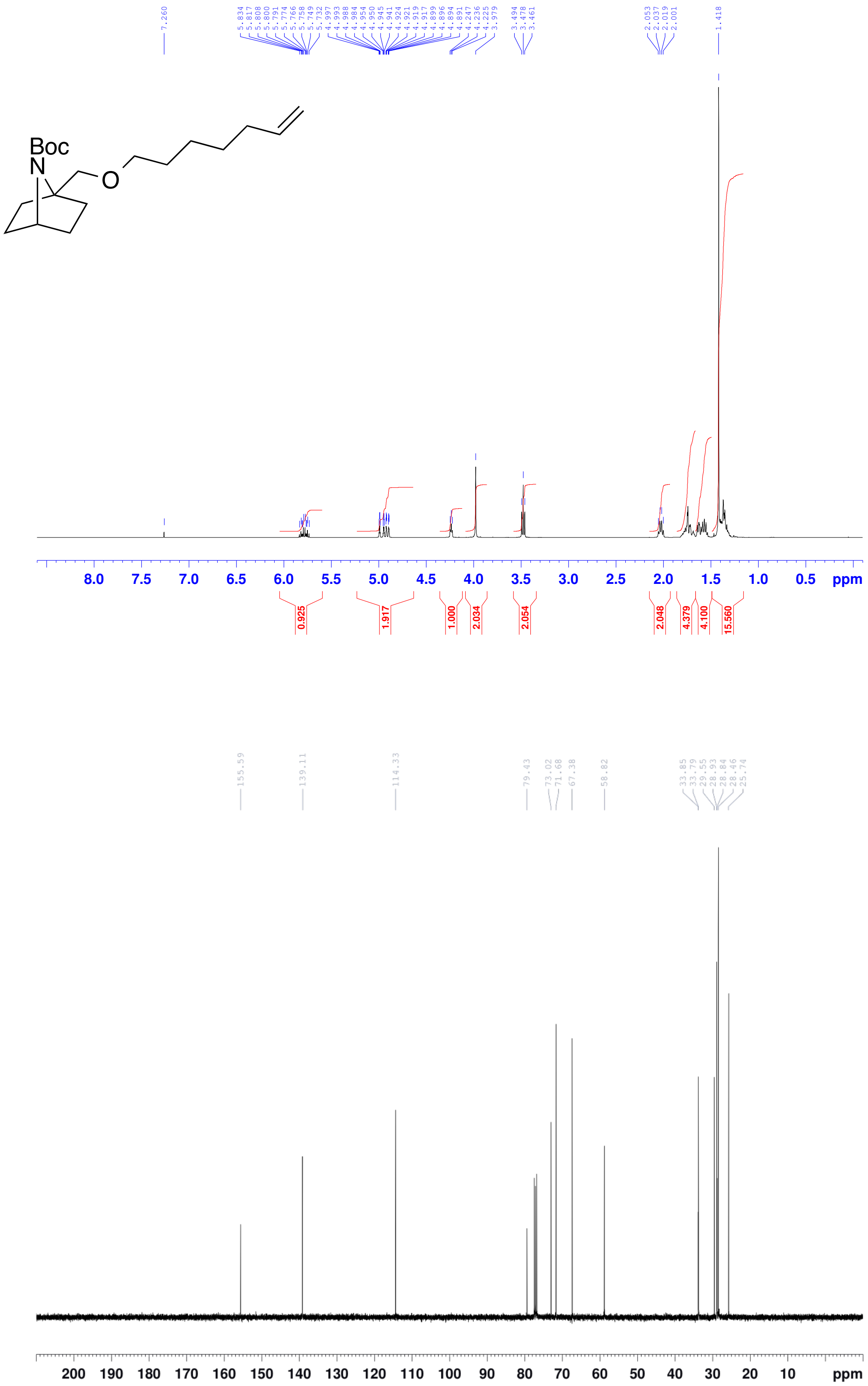

Supplementary Figure 28. ^{1}H and ^{13}C-NMR Charts of Compound 32

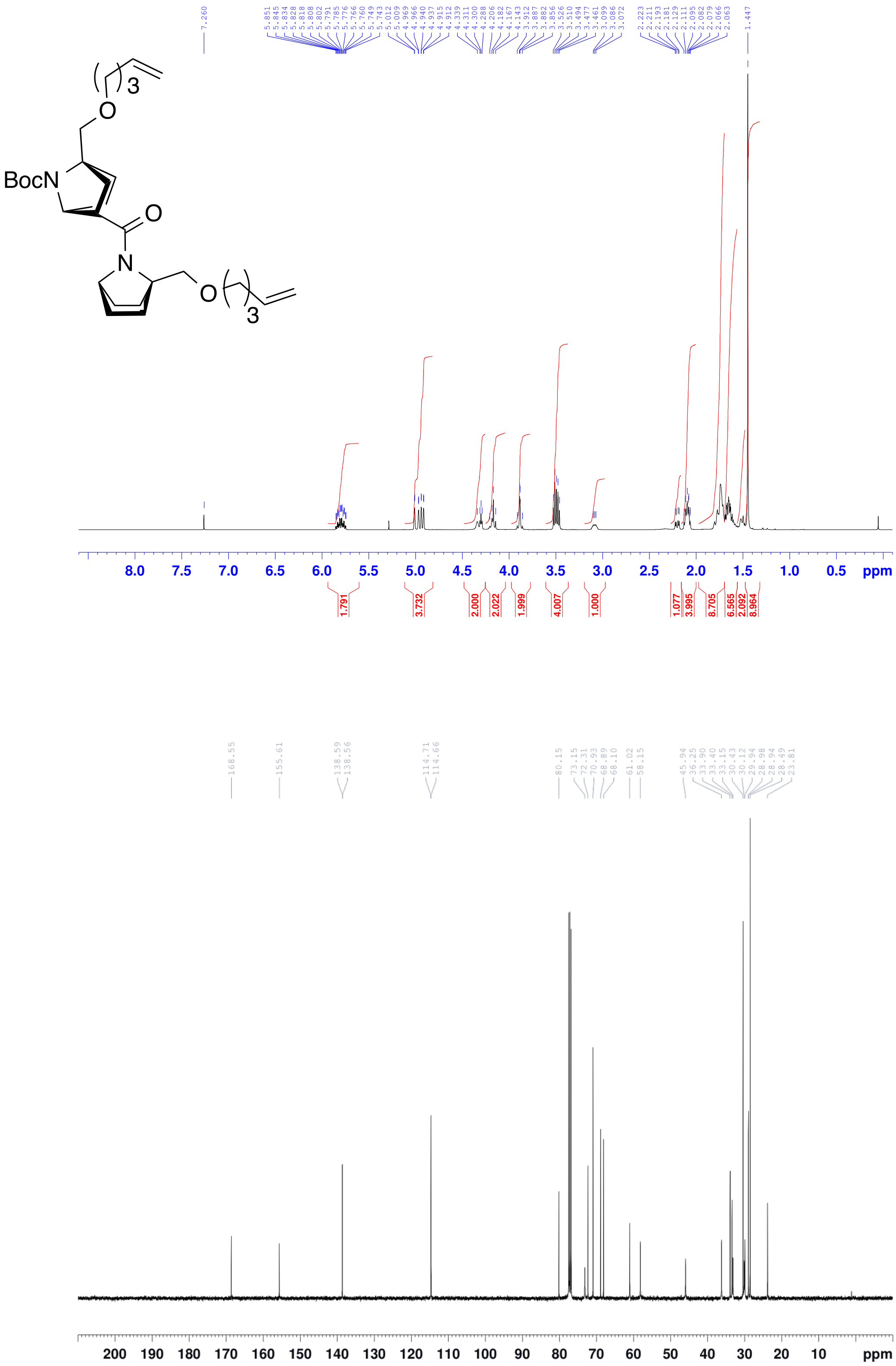

Supplementary Figure 29. ^{1}**H and** ^{13}**C-NMR Charts of Compound 1**

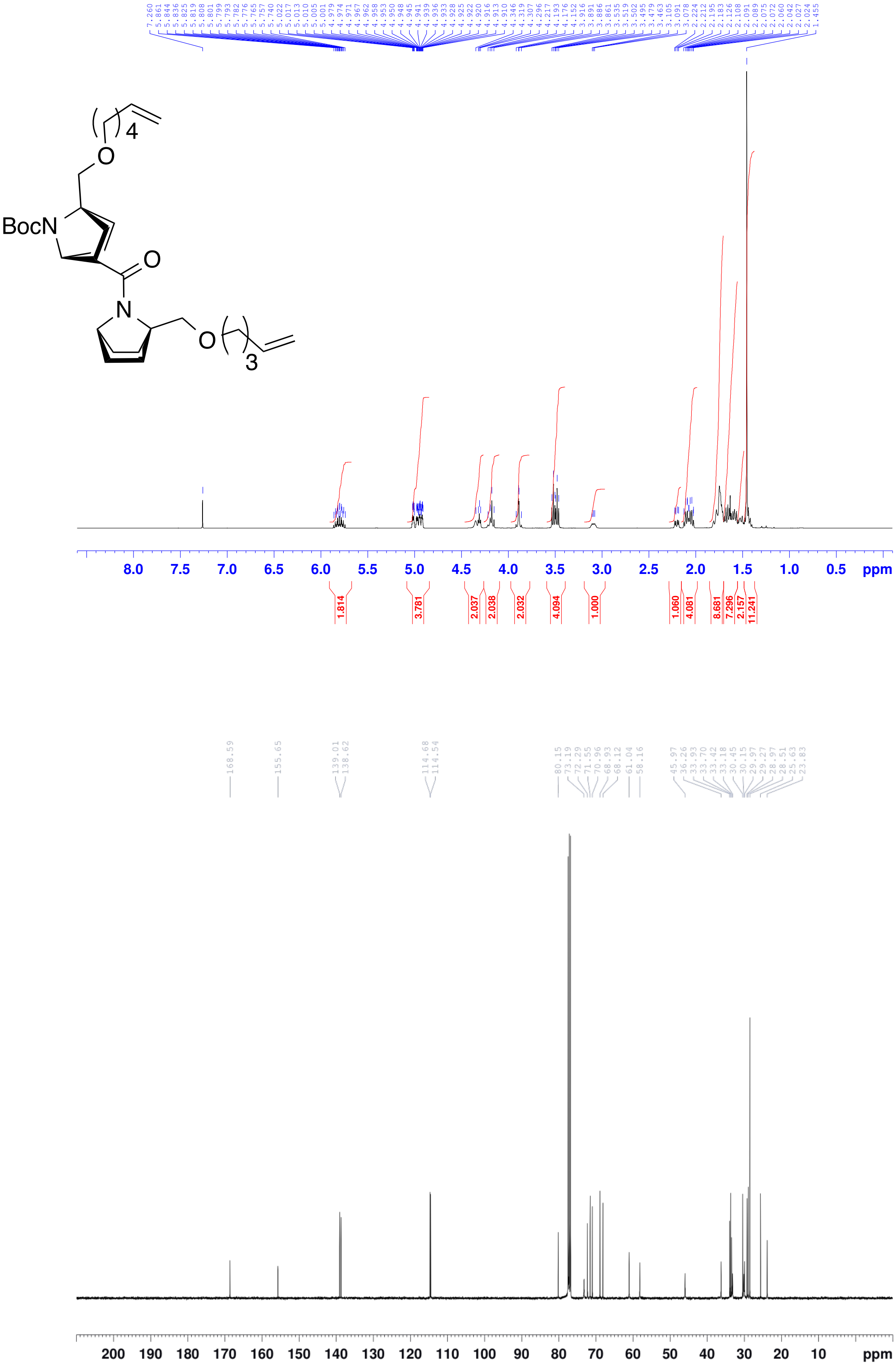

Supplementary Figure 30. ^{1}H and ^{13}C-NMR Charts of Compound 2

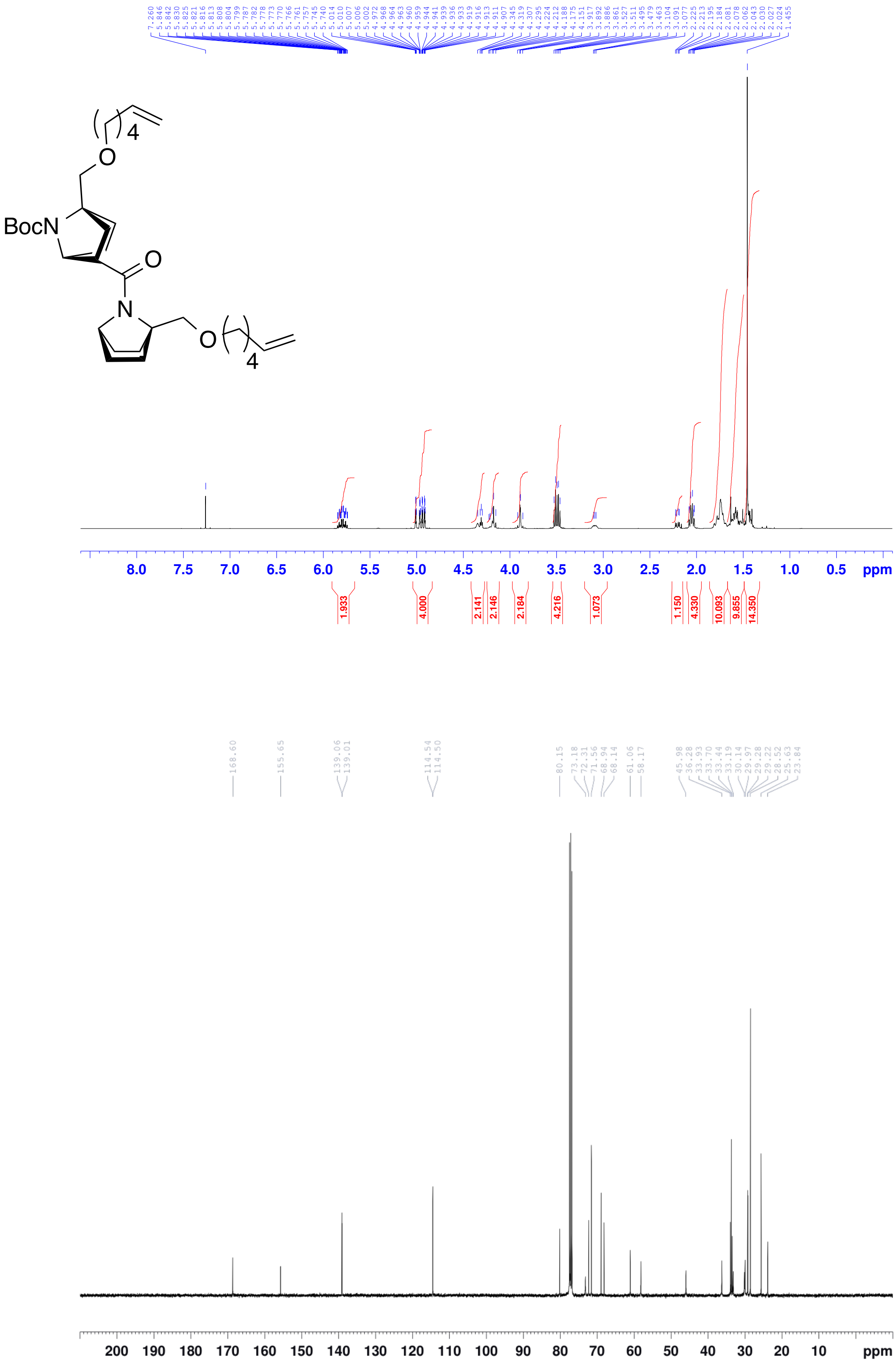

Supplementary Figure 31. ^{1}H and ^{13}C-NMR Charts of Compound 3

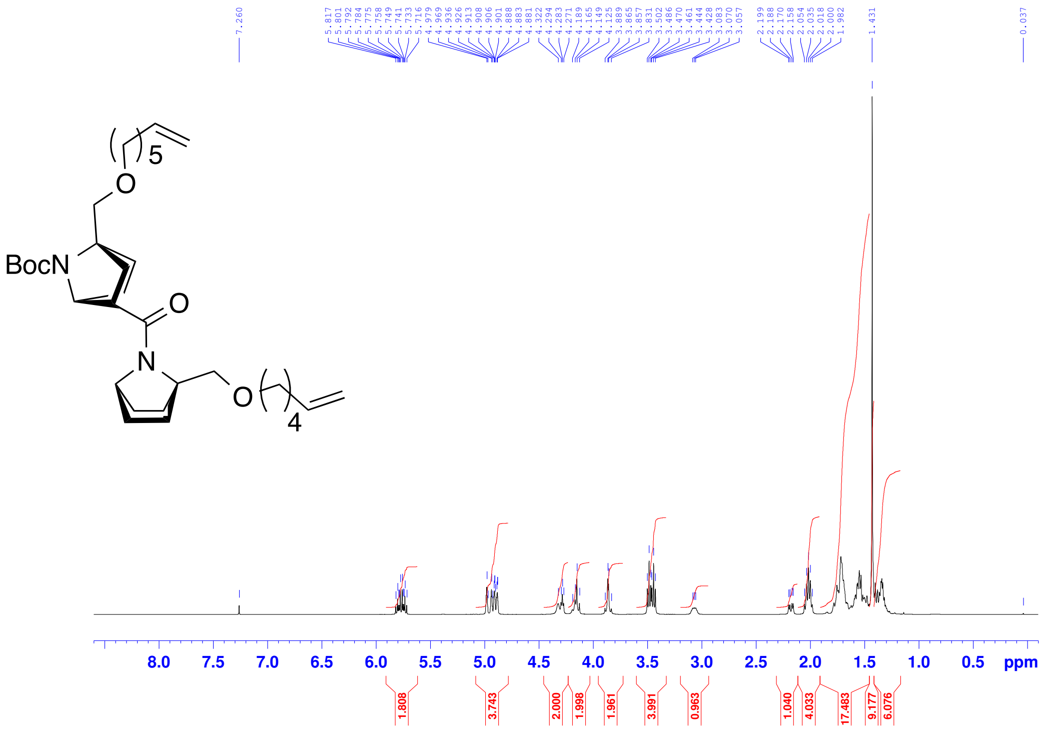

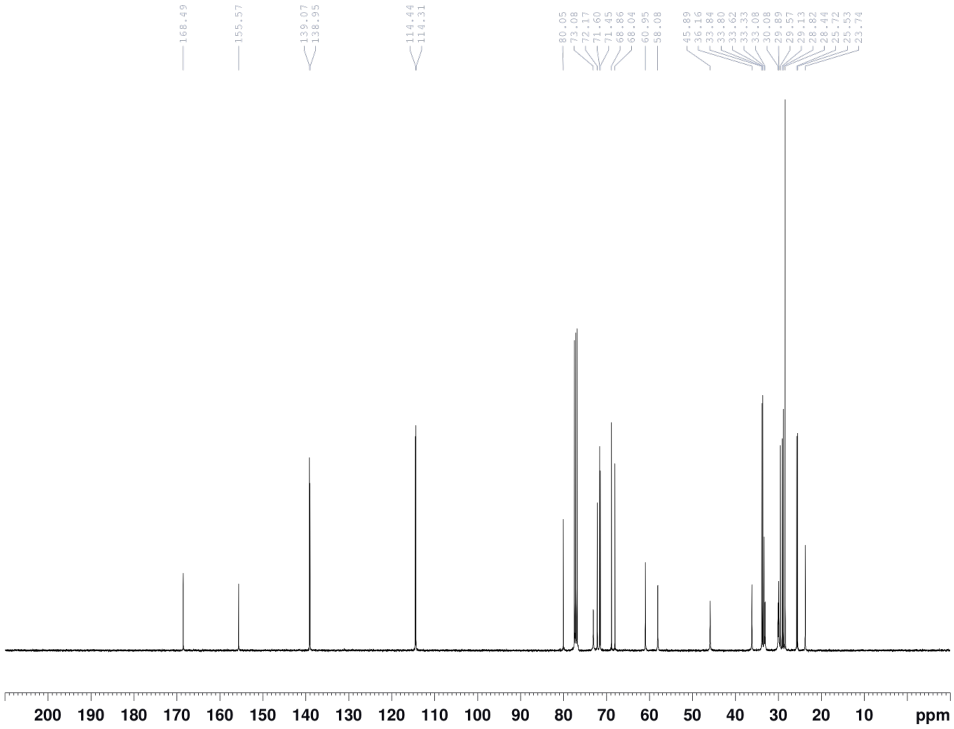

Supplementary Figure 32. ^{1}H and ^{13}C-NMR Charts of Compound 4

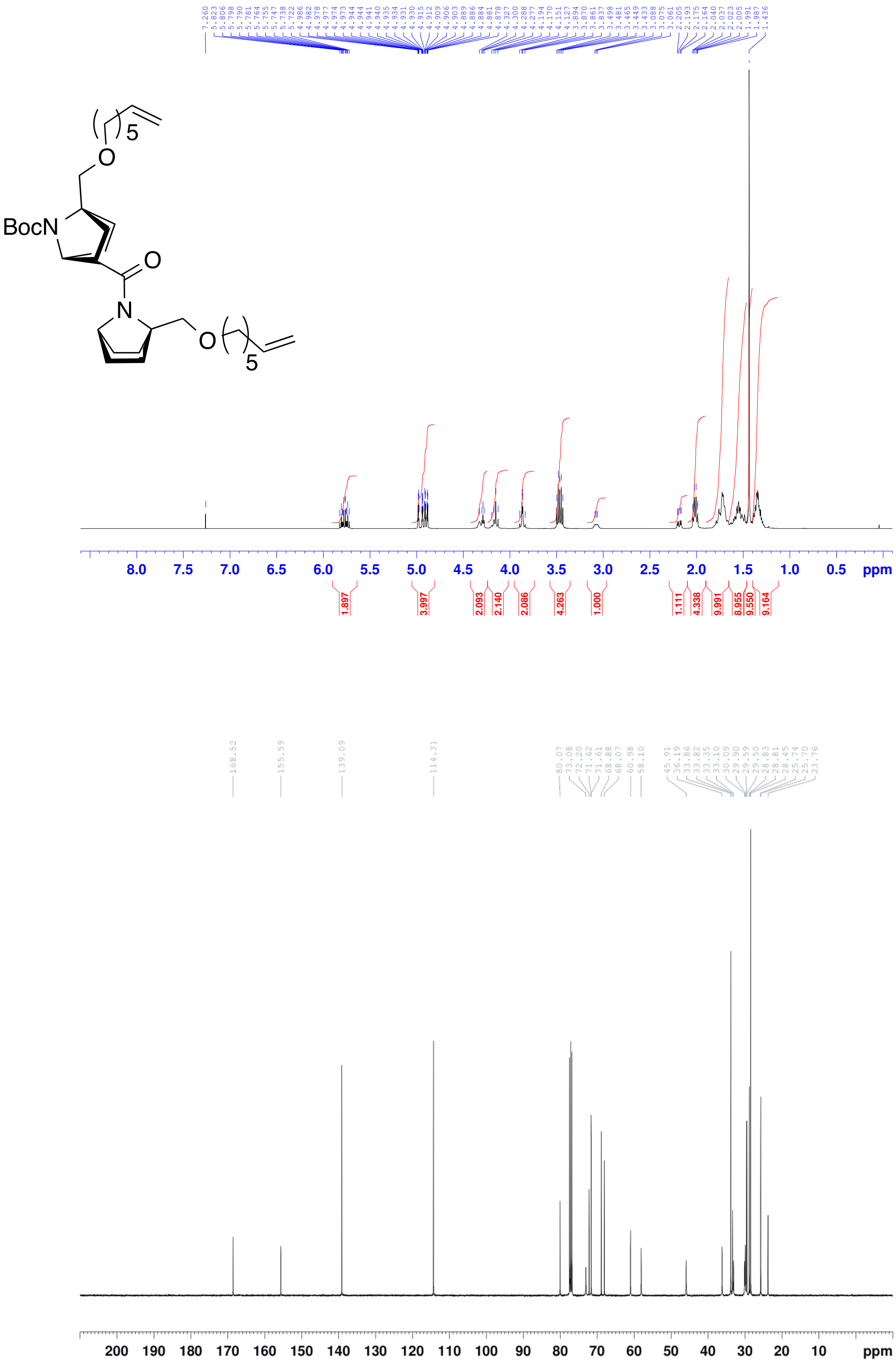

Supplementary Figure 33. ^{1}H and ^{13}C-NMR Charts of Compound 5

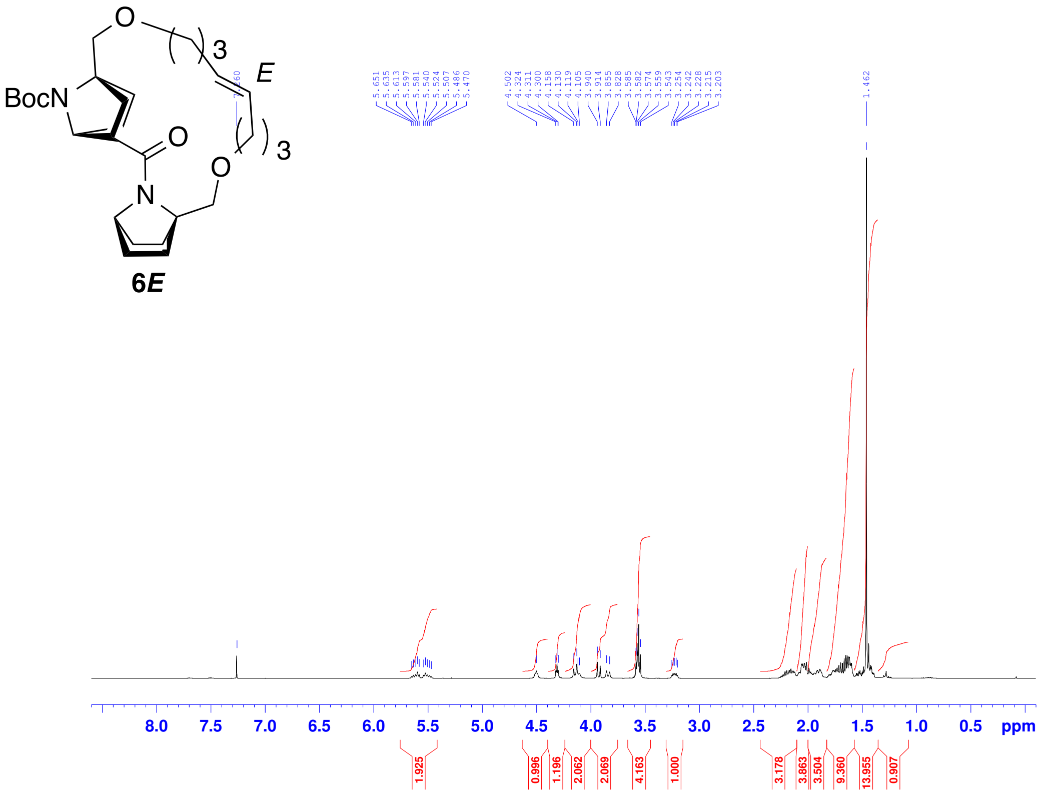

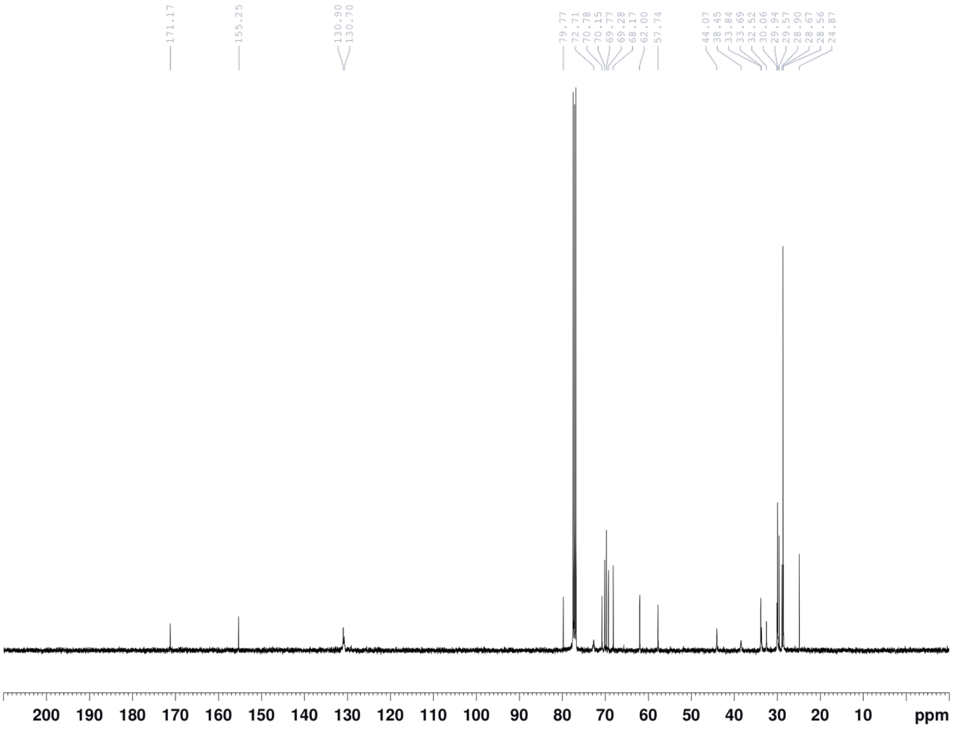

Supplementary Figure 34. ^{1}H and ^{13}C-NMR Charts of Compound 6(*E*) (60 °C)

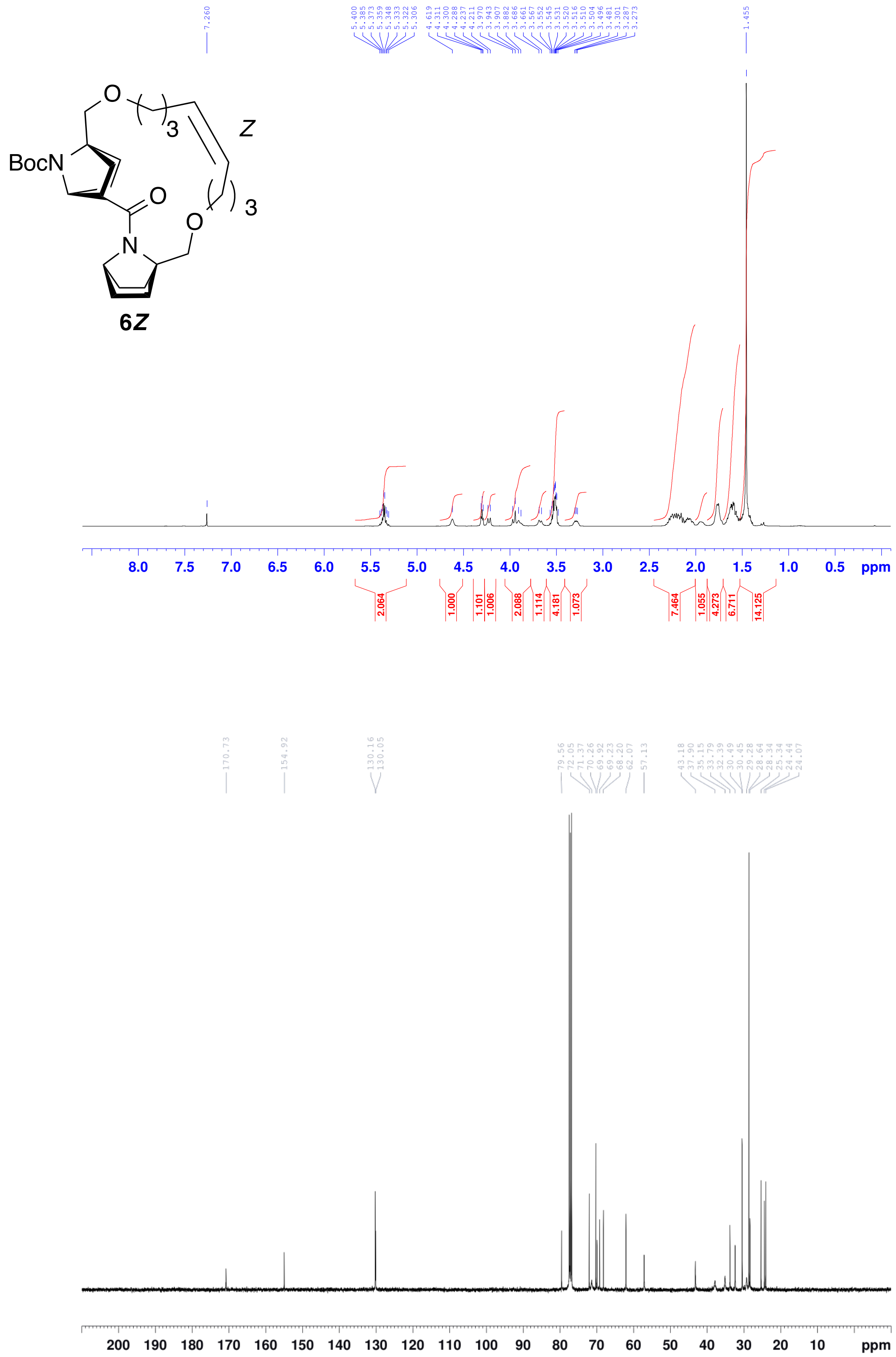

Supplementary Figure 35. ^{1}**H and** ^{13}**C-NMR Charts of Compound 6(*Z*) (60 °C)**

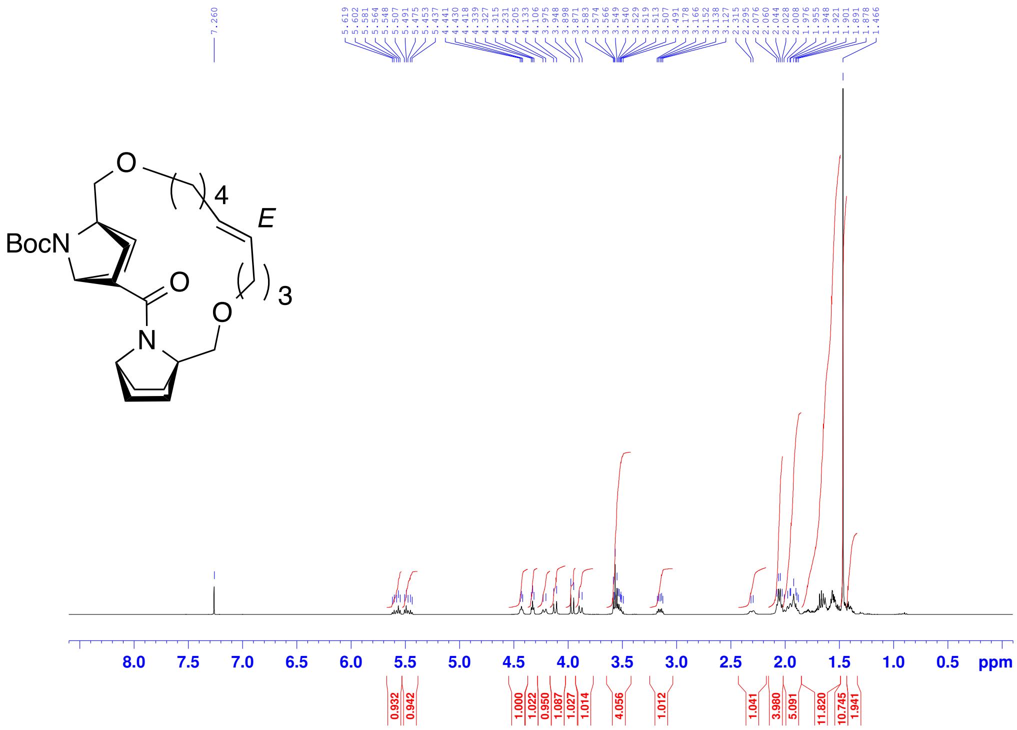

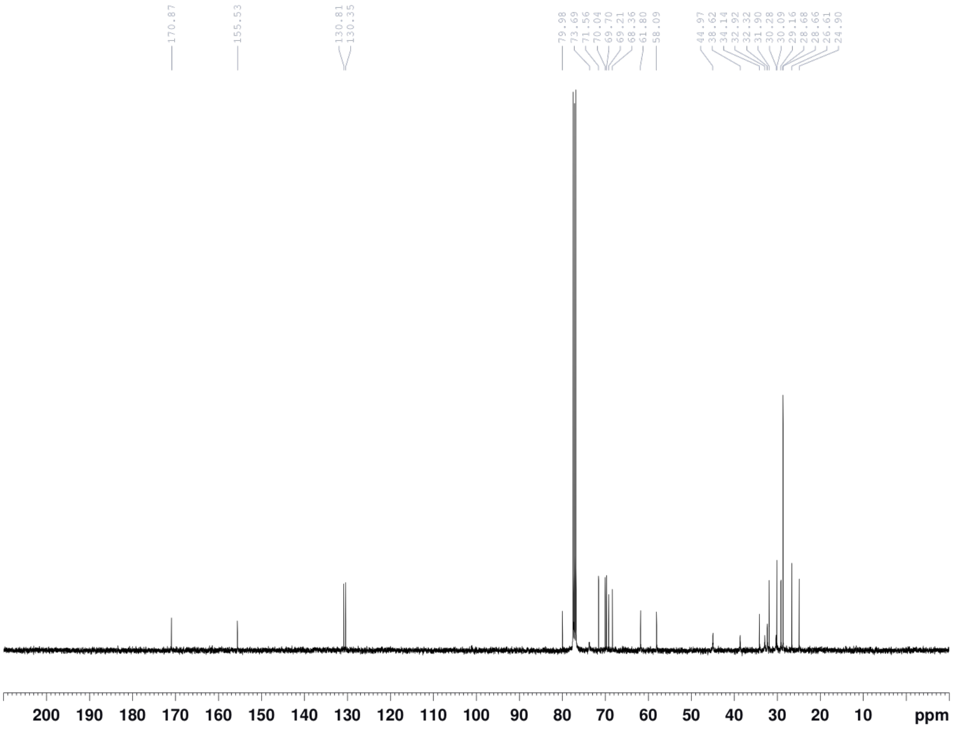

Supplementary Figure 36. ^{1}**H and** ^{13}**C-NMR Charts of Compound 7(*E*) (60 °C)**

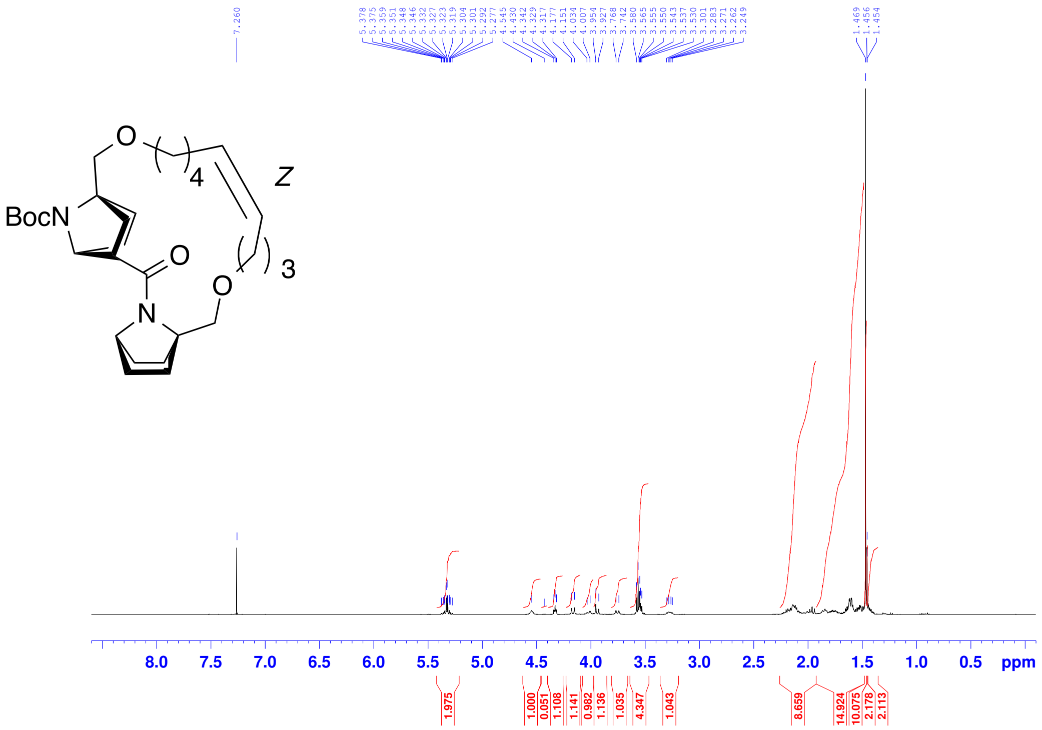

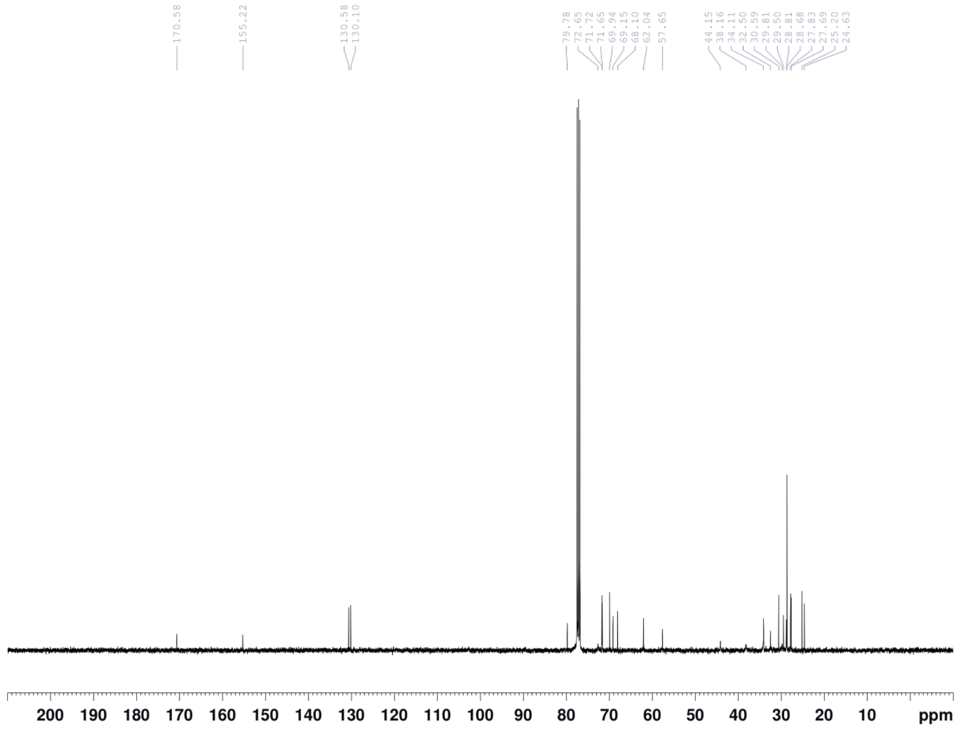

Supplementary Figure 37. ^{1}H and ^{13}C-NMR Charts of Compound 7(*Z*) (60 °C)

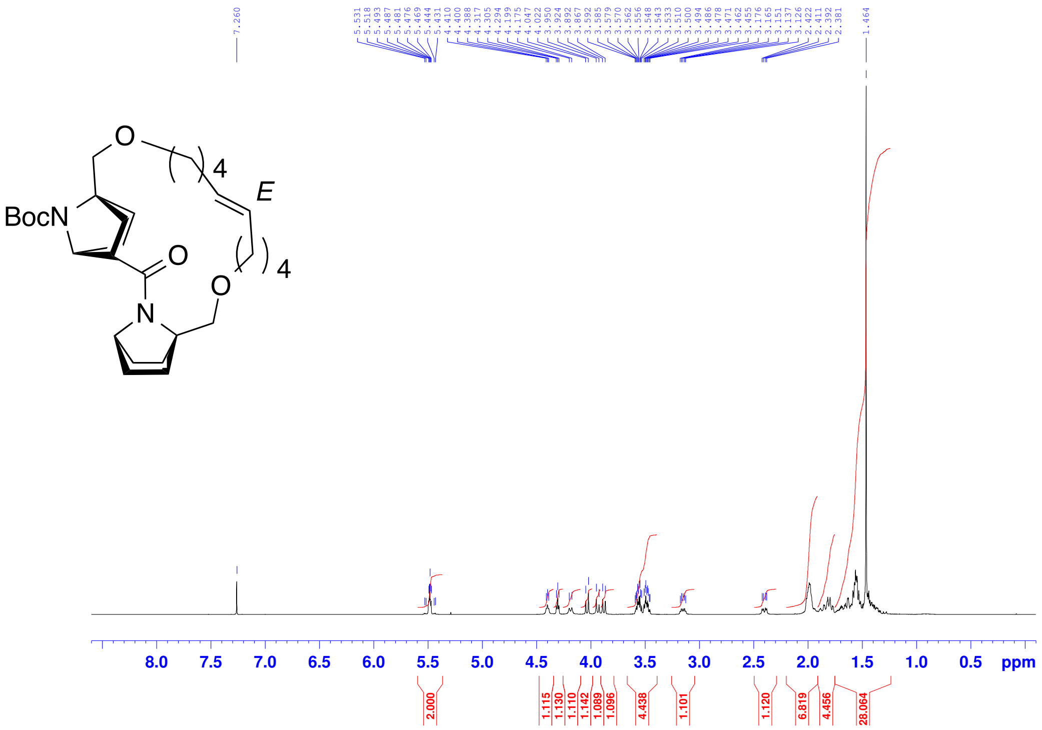

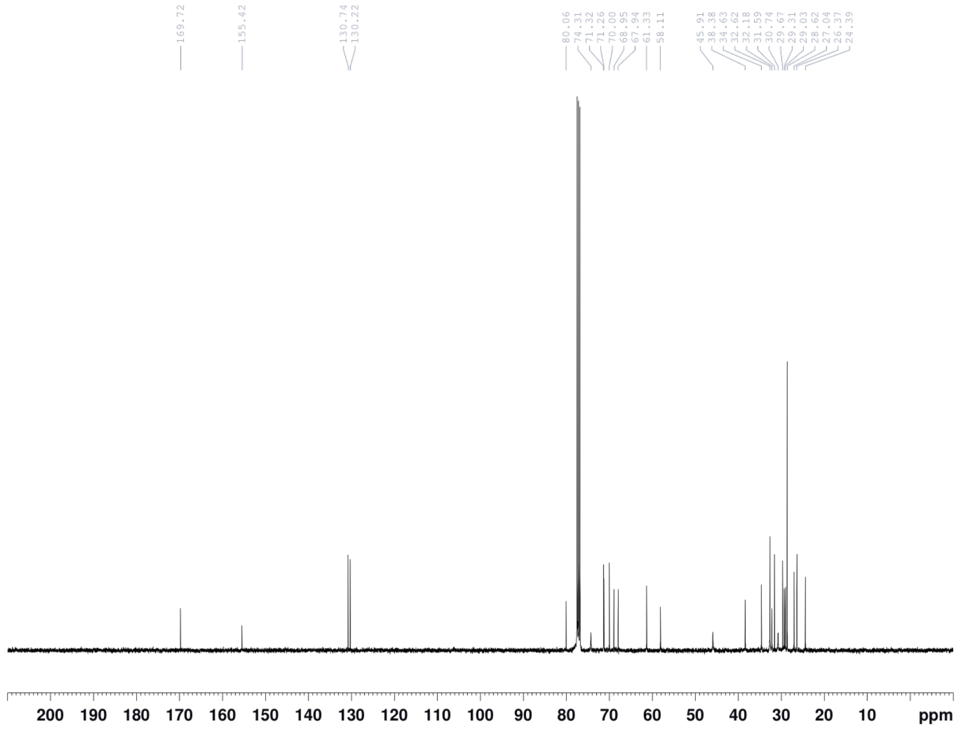

Supplementary Figure 38. ^{1}H and ^{13}C-NMR Charts of Compound 8(*E*) (55 °C)

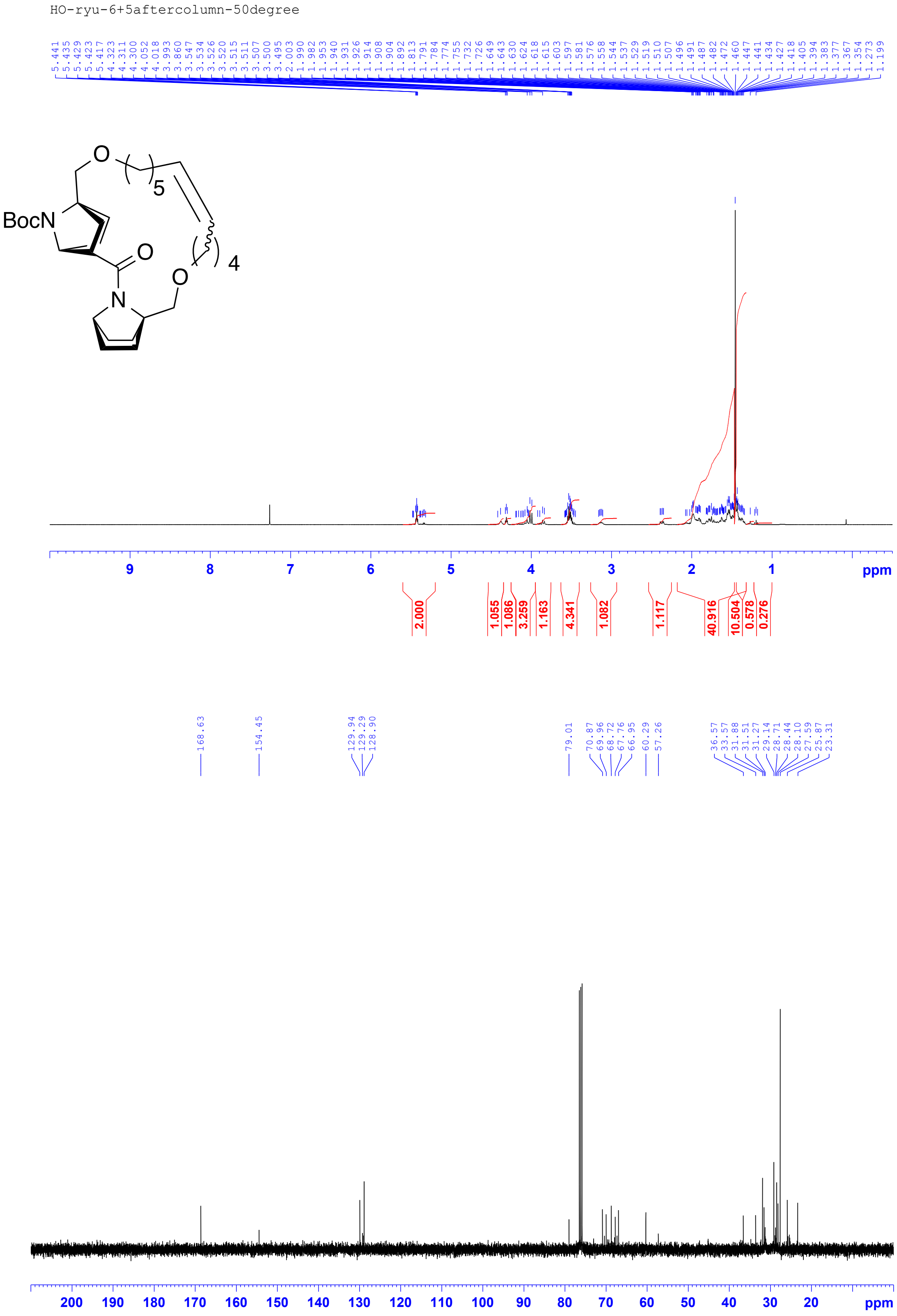

Supplementary Figure 39. ^{1}H and ^{13}C-NMR Charts of Compound 9 (50 °C)

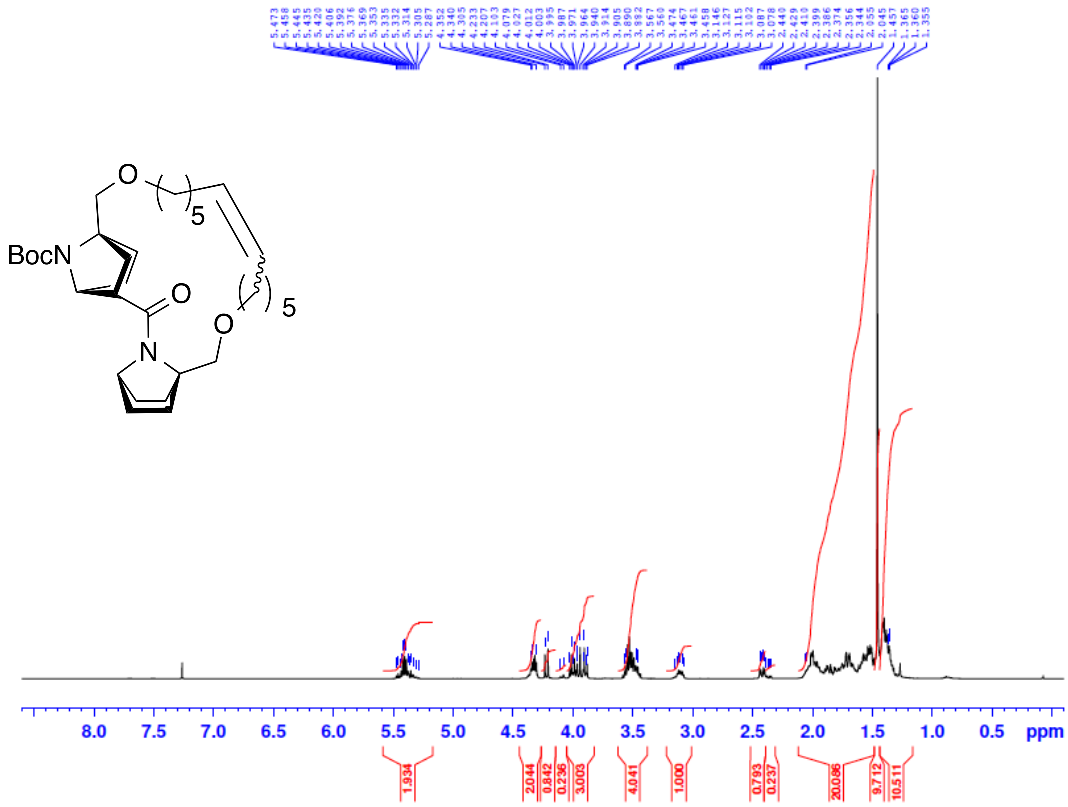

(20 °C)

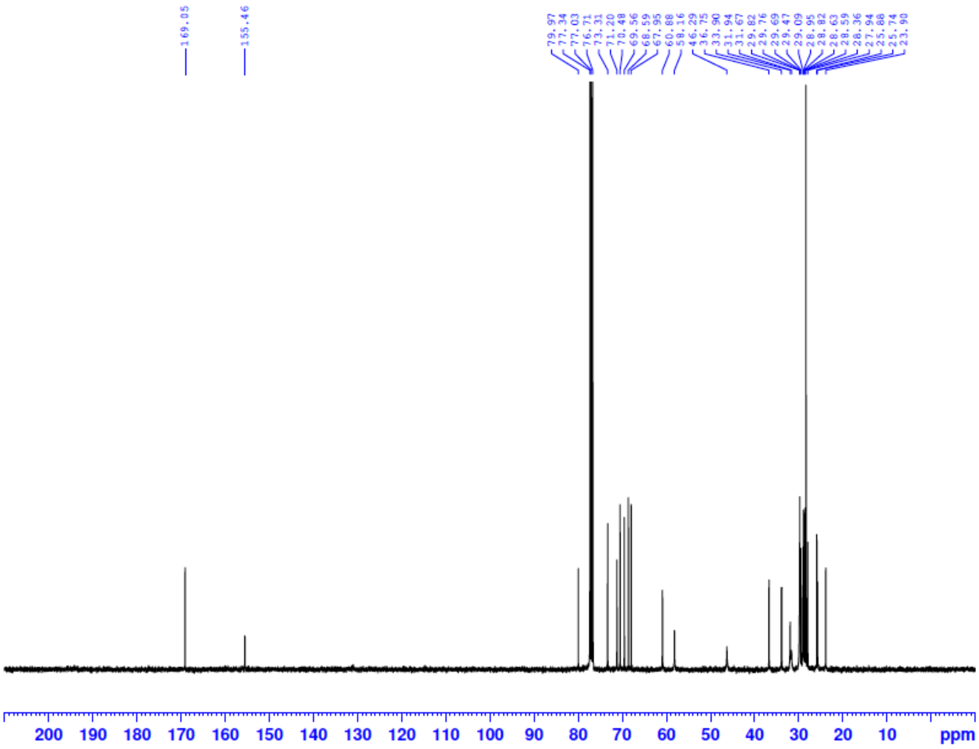

Supplementary Figure 40. ^{1}H and ^{13}C-NMR Charts of Compound 10 (55 °C)

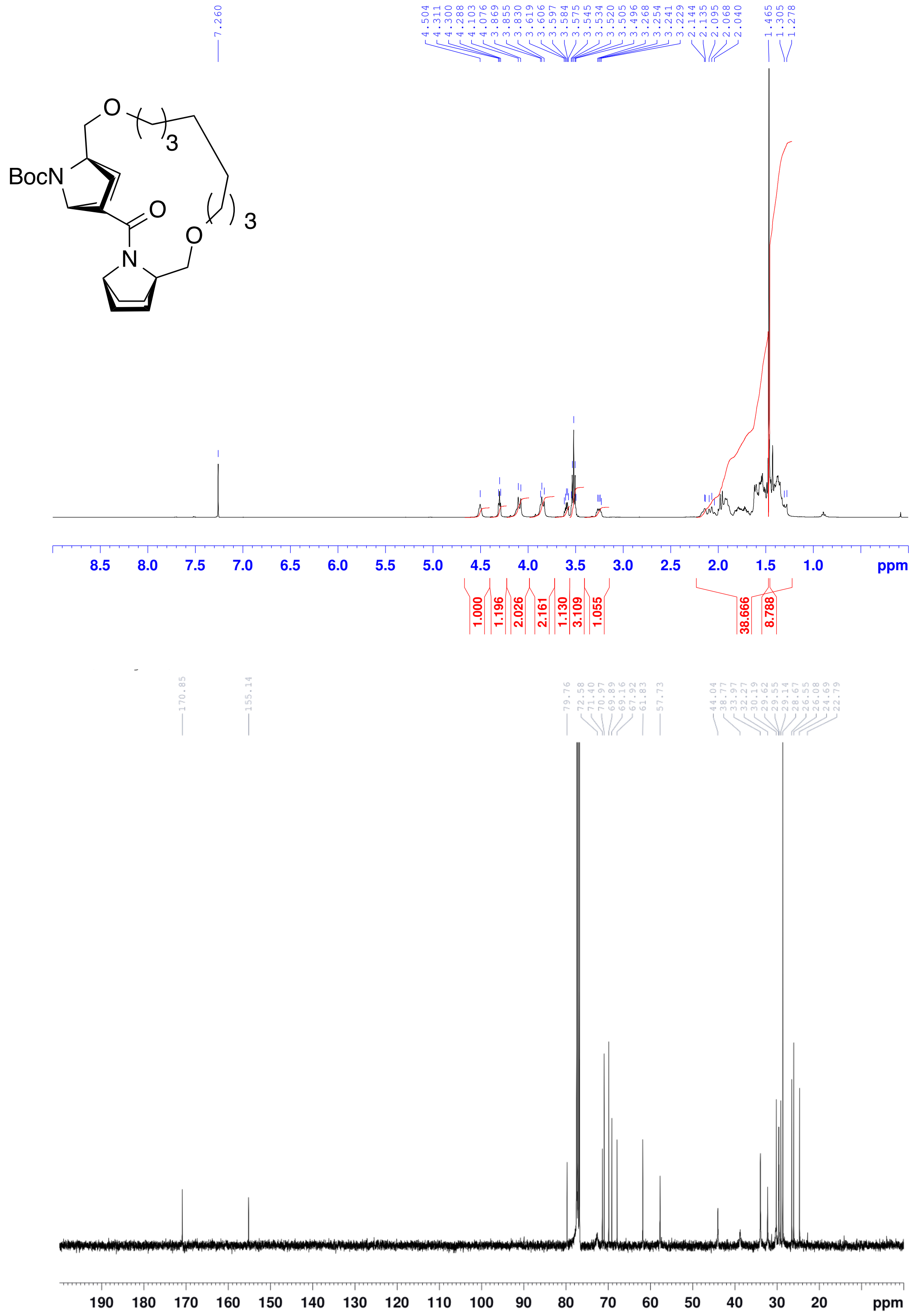

Supplementary Figure 41. **^{1}H and ^{13}C-NMR Charts of Compound 11 (55 °C)**

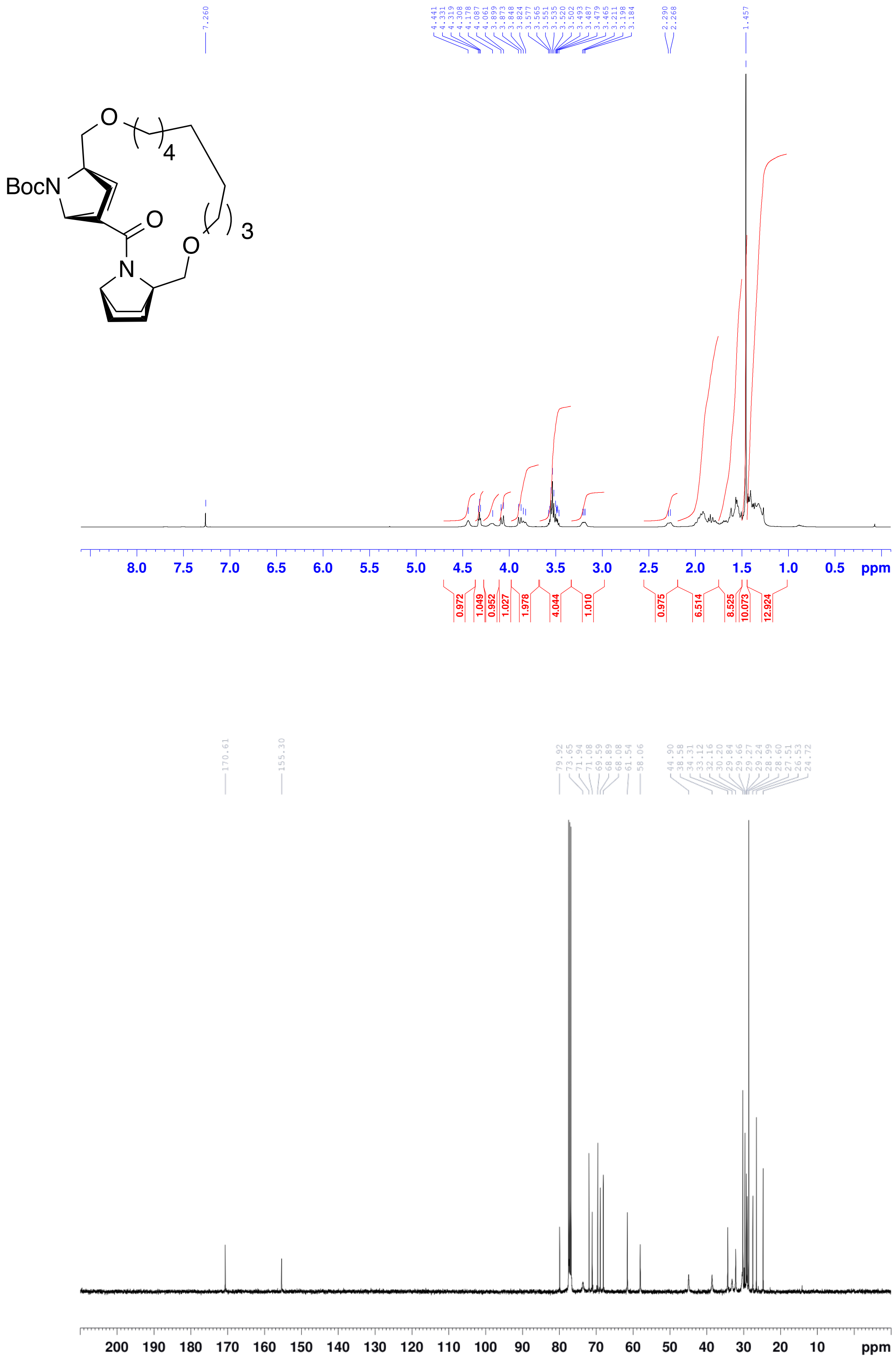

Supplementary Figure 42. ^{1}**H and** ^{13}**C-NMR Charts of Compound 12 (50 °C)**

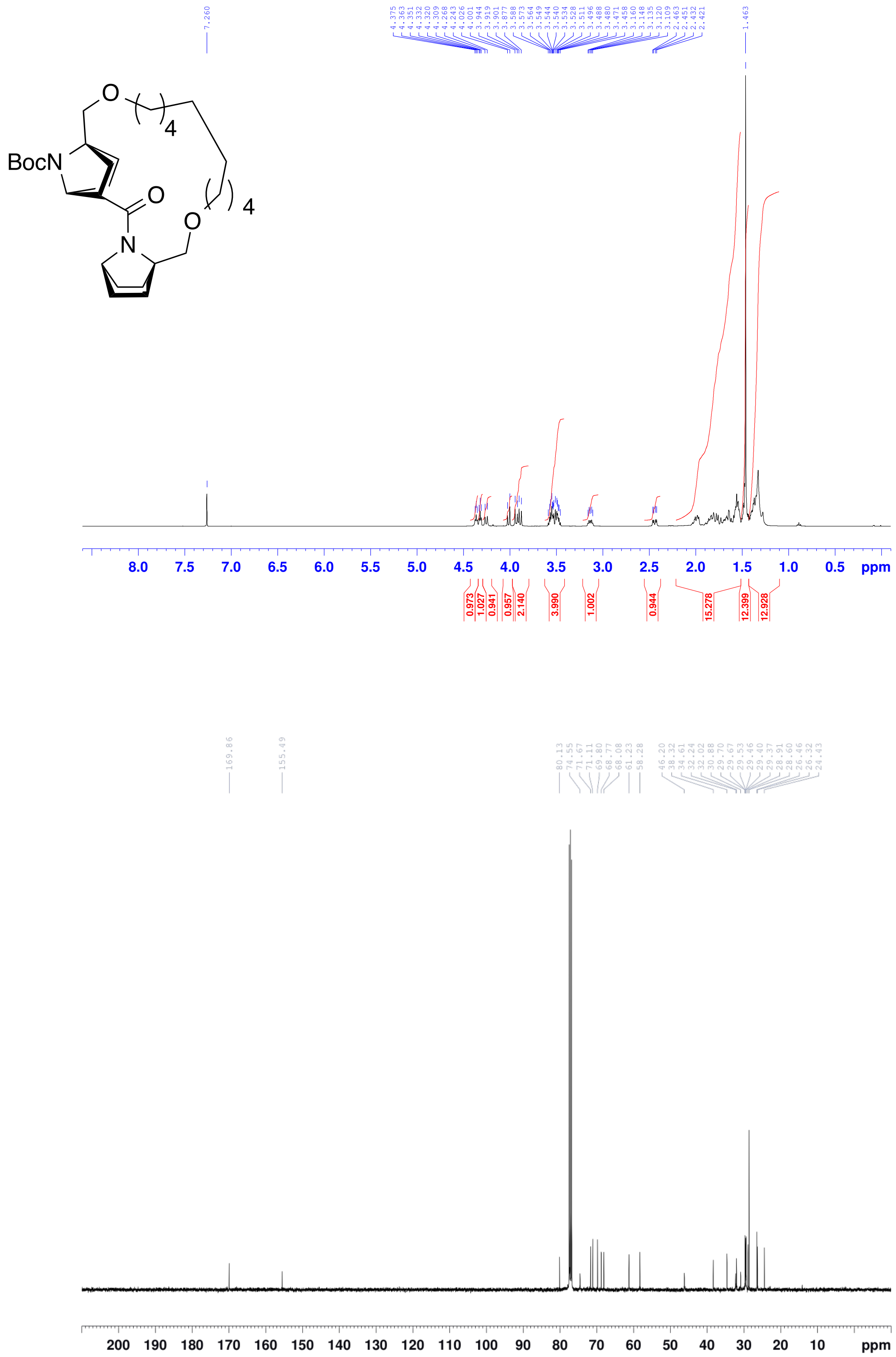

Supplementary Figure 43. ^{1}H and ^{13}C-NMR Charts of Compound 13 (50 °C)

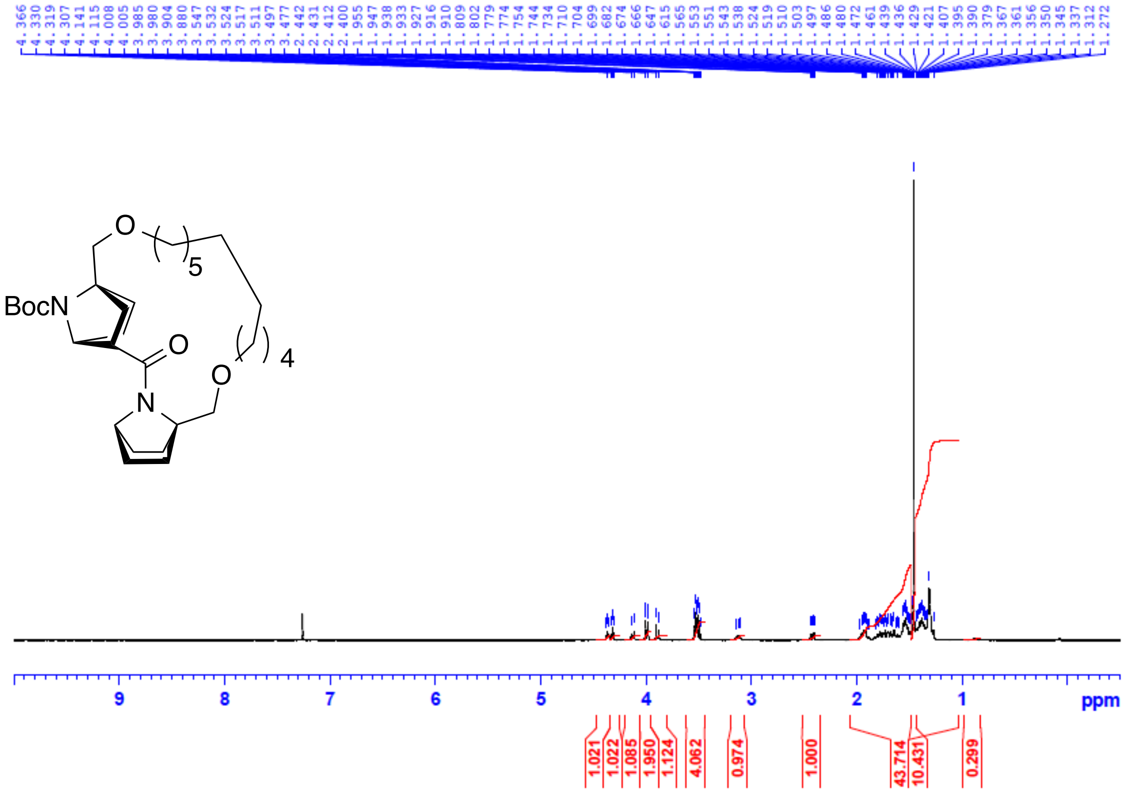

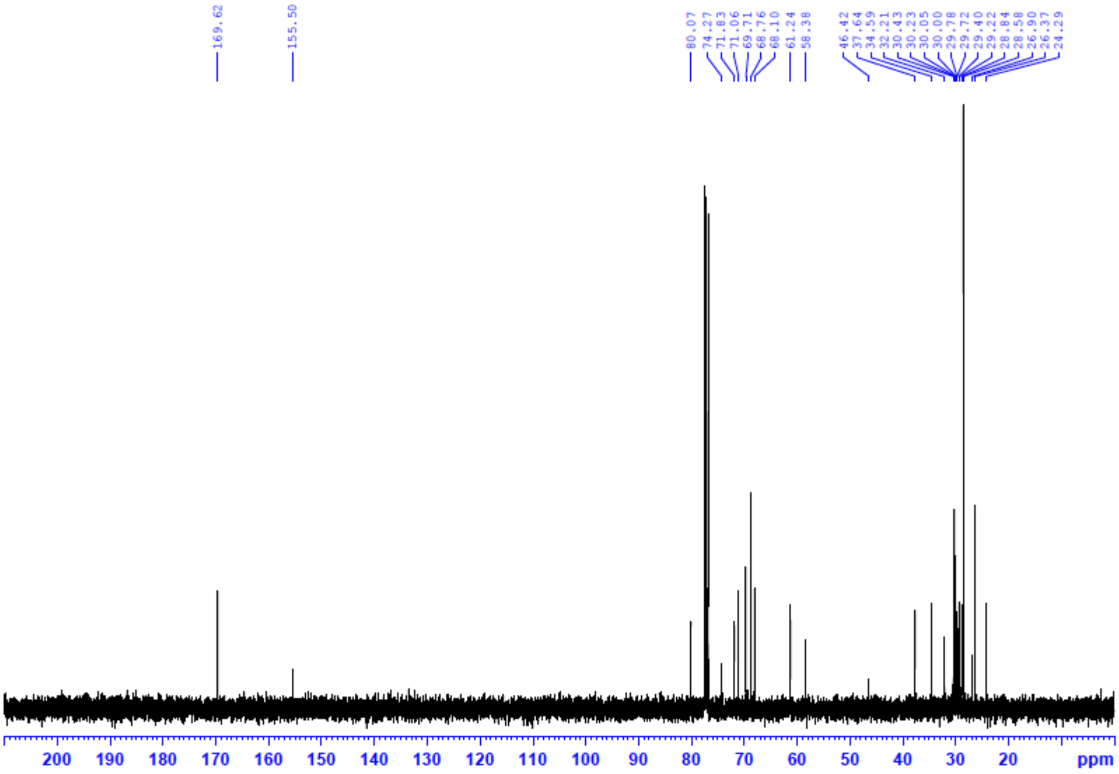

Supplementary Figure 44. ^{1}**H and** ^{13}**C-NMR Charts of Compound 14 (50 °C)**

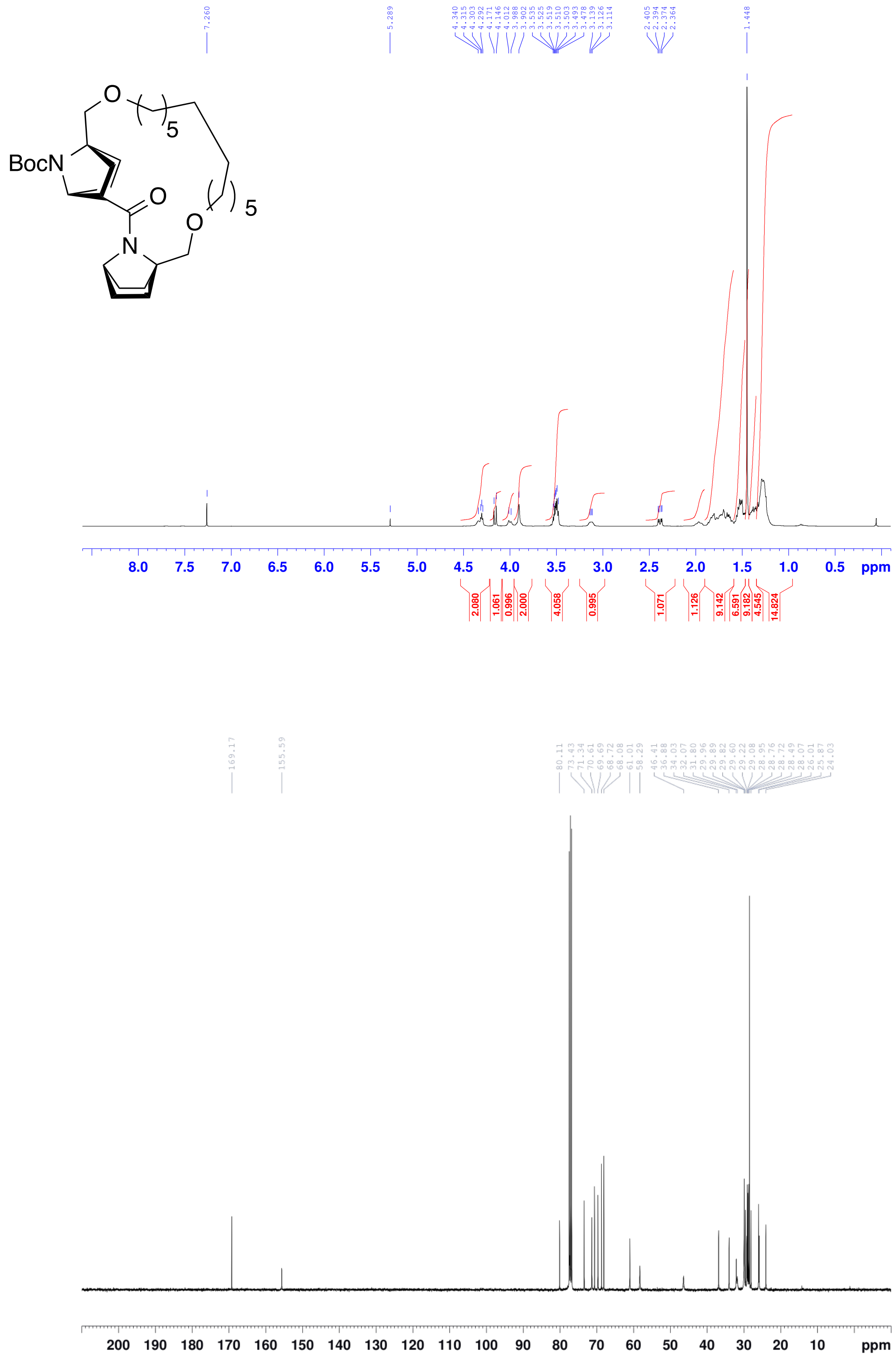

Supplementary Figure 45. ^{1}H and ^{13}C-NMR Charts of Compound 15

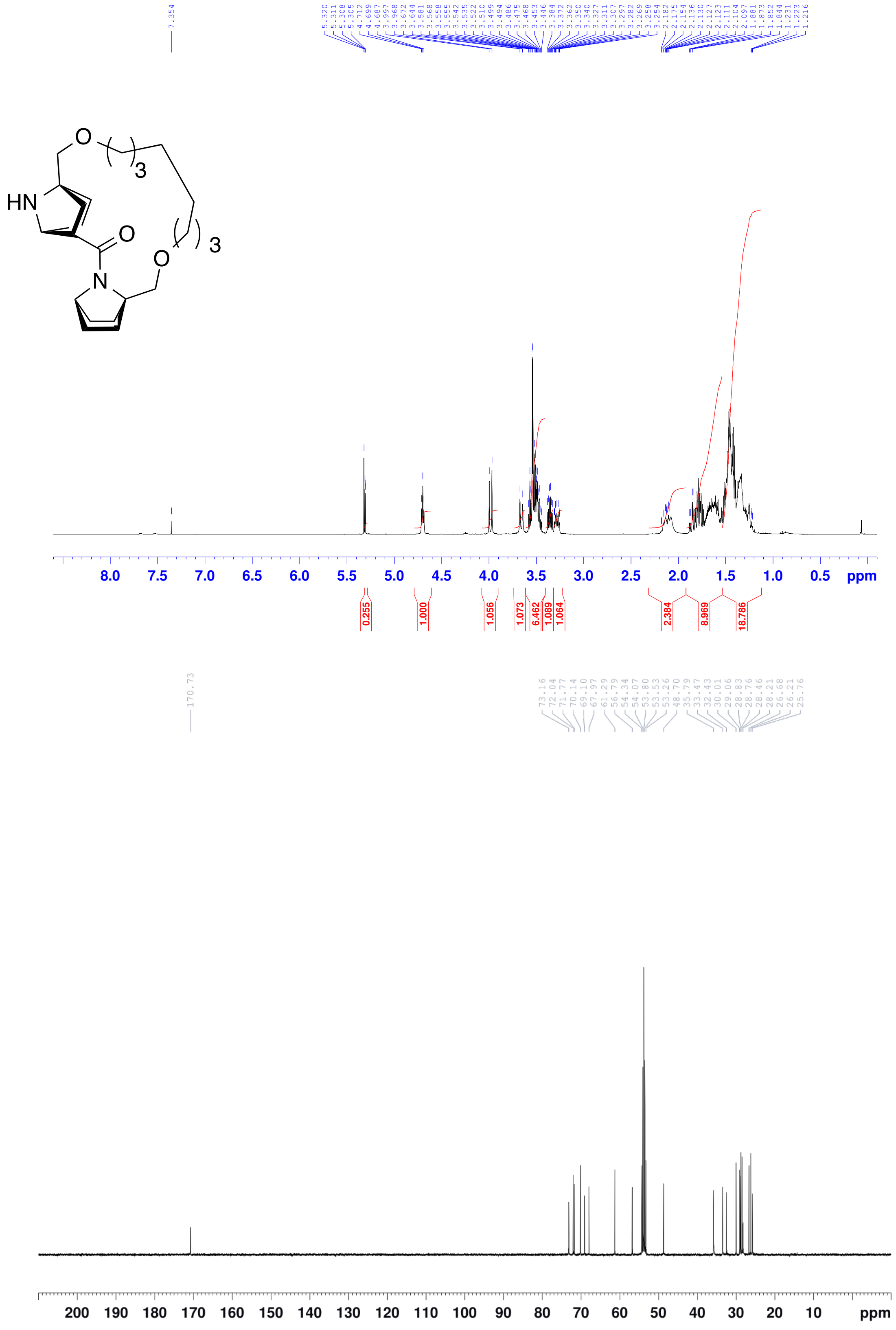

Supplementary Figure 46. ^{1}H and ^{13}C-NMR Charts of Compound 16(C8)

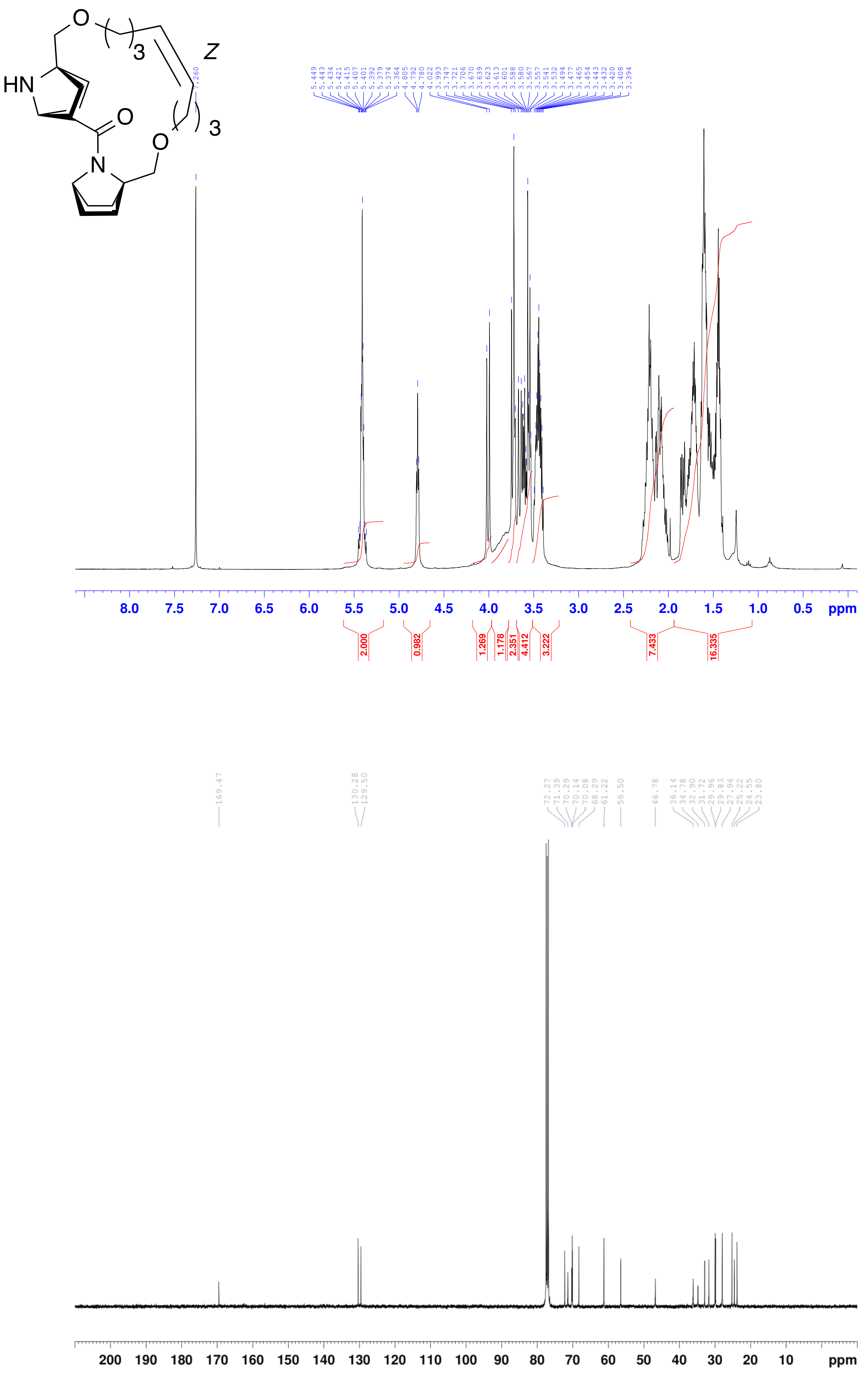

Supplementary Figure 47. ^{1}H and ^{13}C-NMR Charts of Compound 6(*Z*)-NH

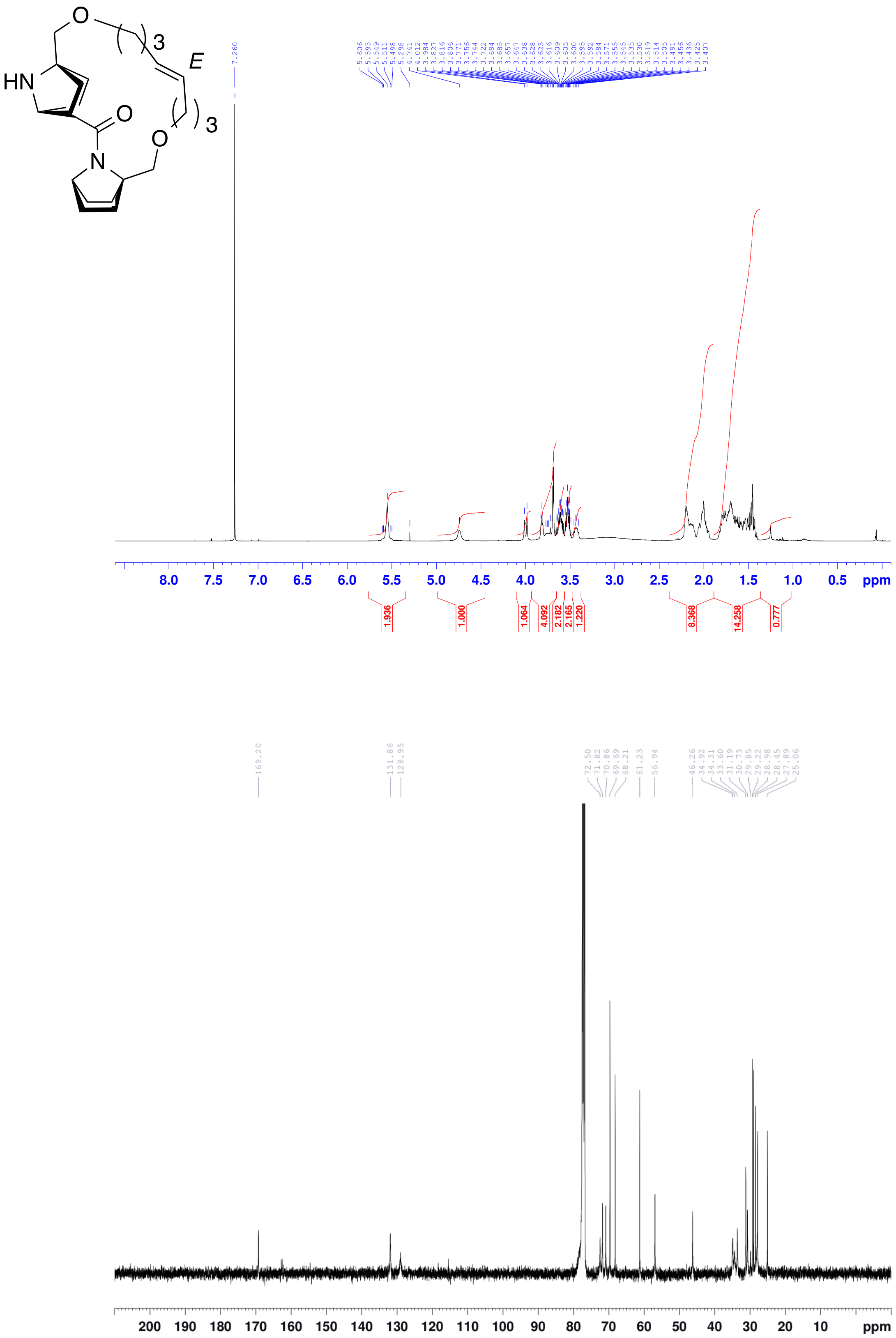

Supplementary Figure 48. ^{1}H and ^{13}C-NMR Charts of Compound 6(*E*)-NH

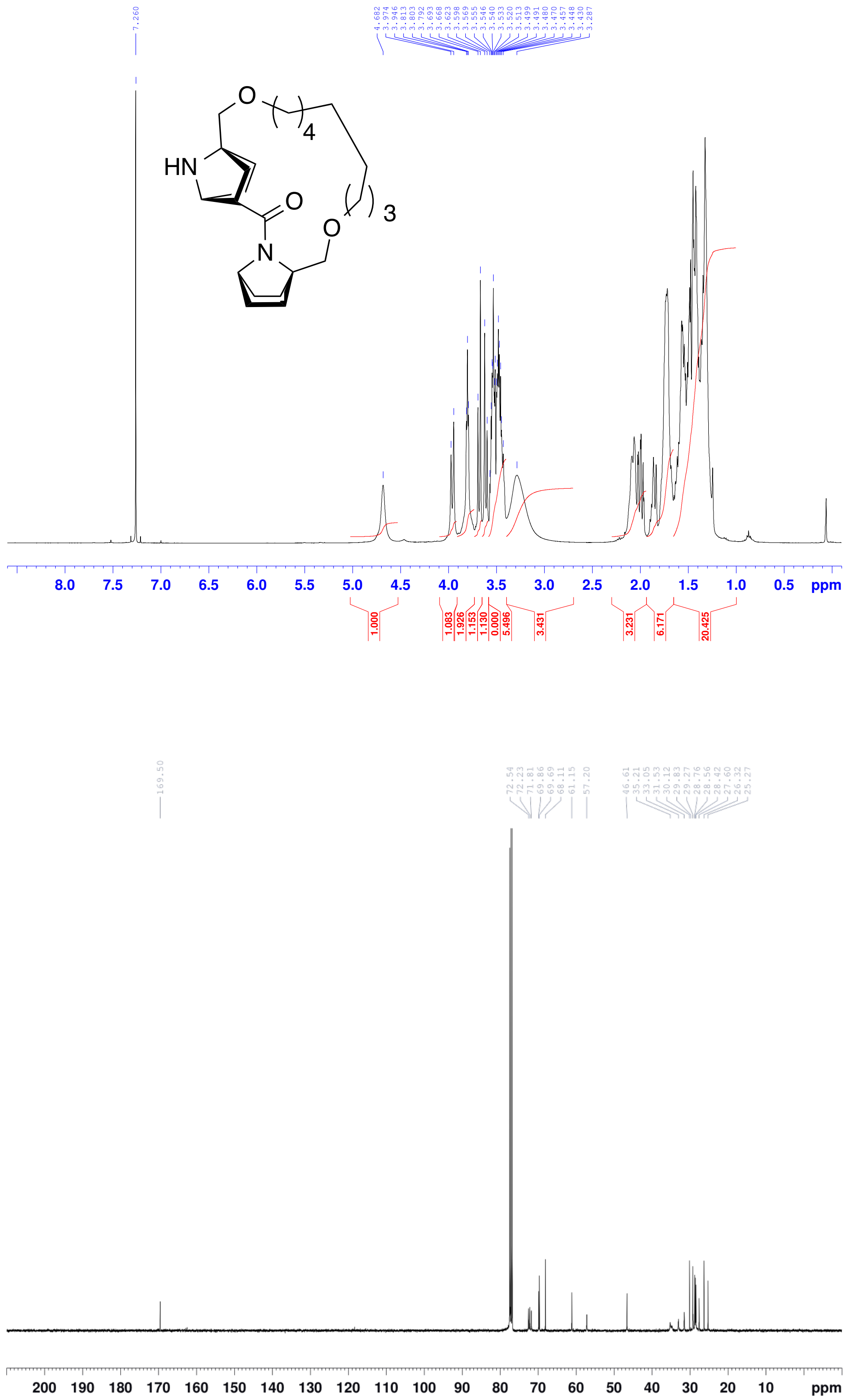

Supplementary Figure 49. ^{1}H and ^{13}C-NMR Charts of Compound 17(C9)

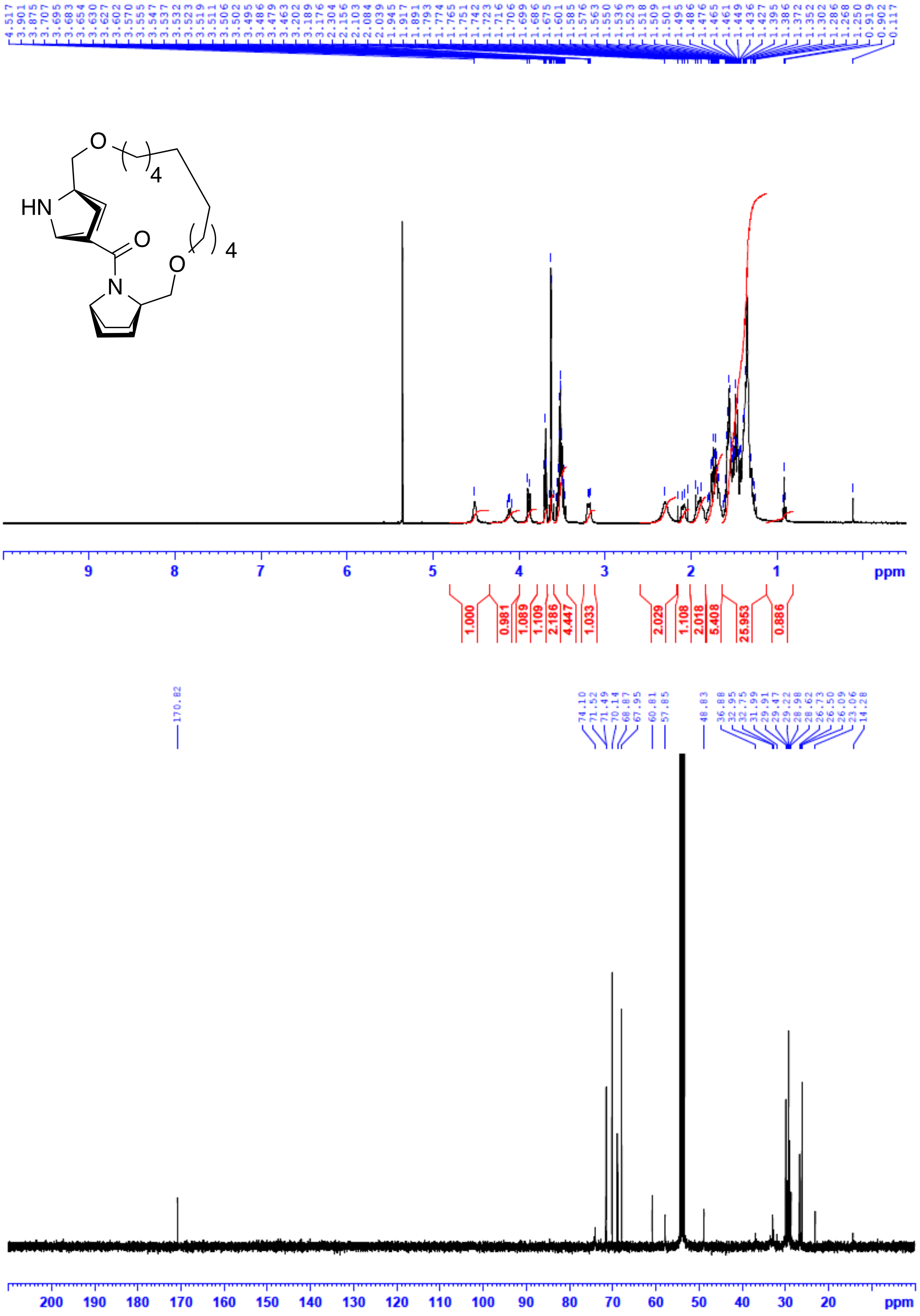

Supplementary Figure 50. ^{1}H and ^{13}C-NMR Charts of Compound 18(C10) (50 °C)

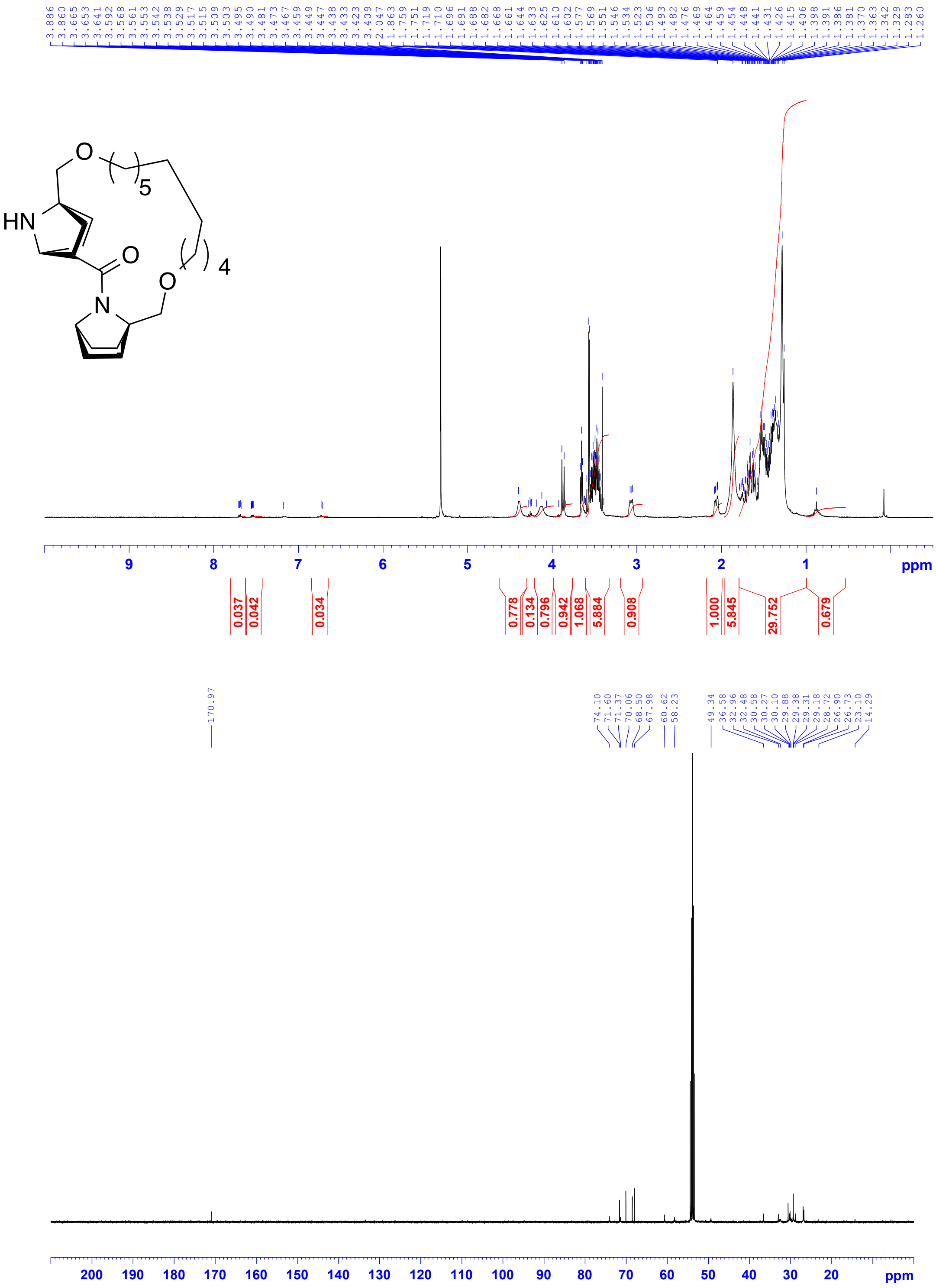

Supplementary Figure 51 ^{1}H and ^{13}C-NMR Charts of Compound 19(C11) (50 °C)

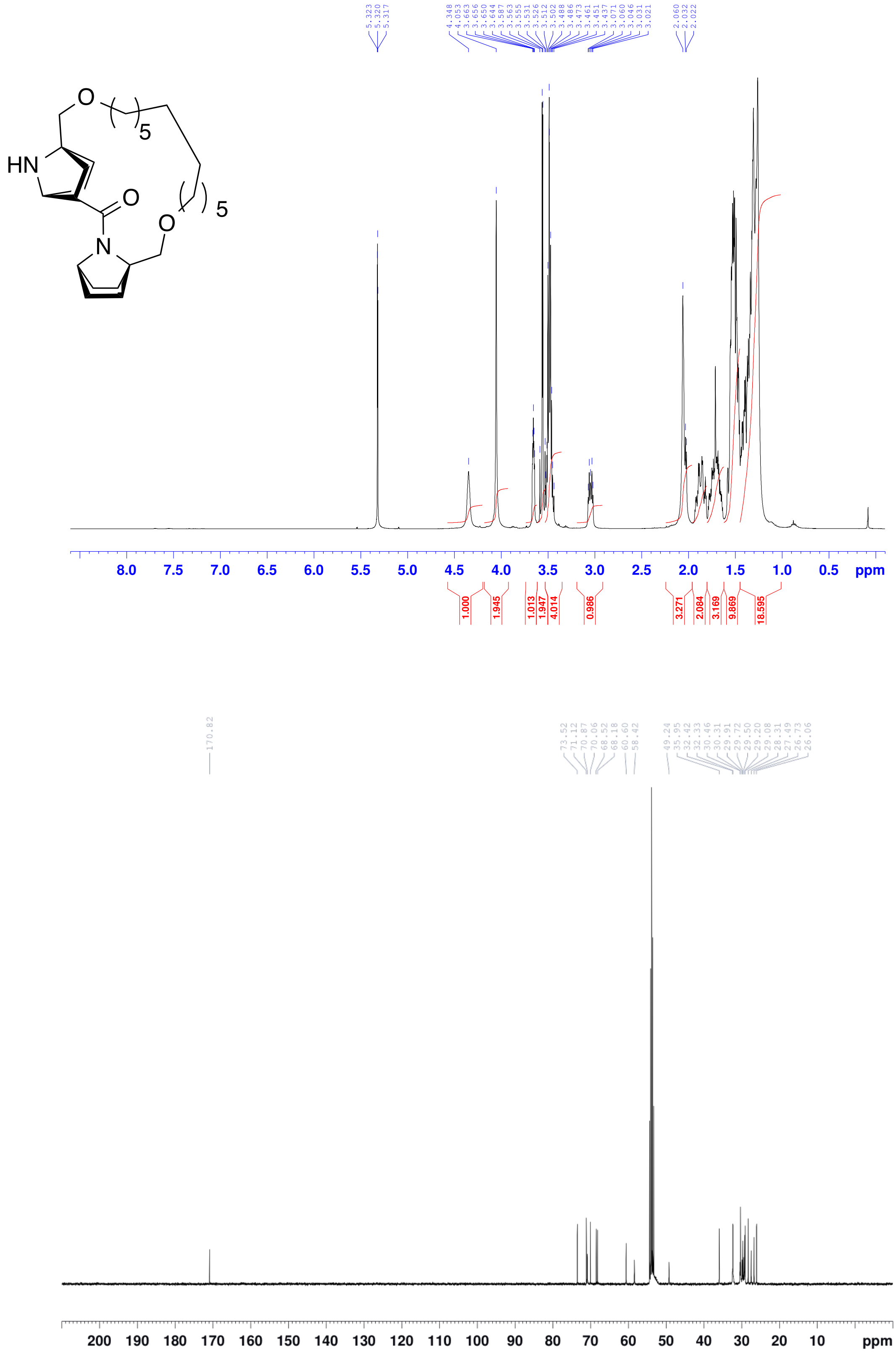

Supplementary Figure 52. ^{1}H and ^{13}C-NMR Charts of Compound 20(C12)

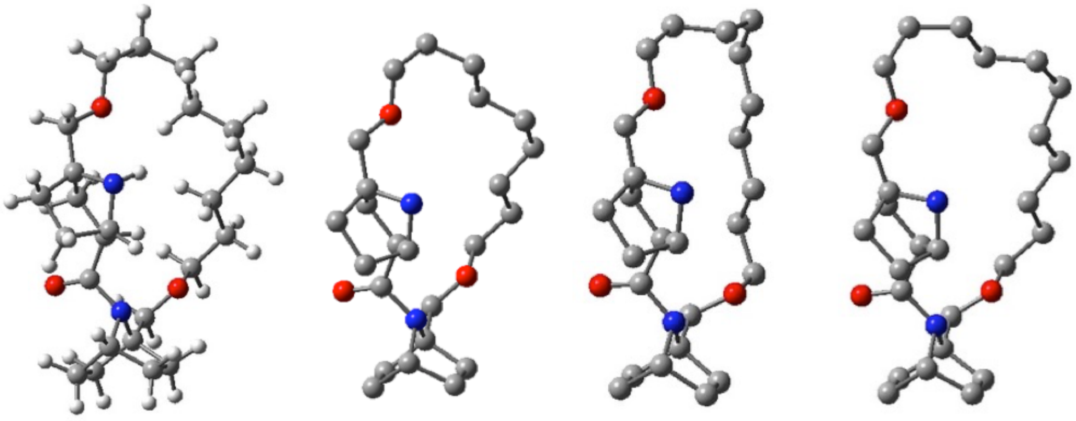

TS- 17 (C9) **TS- 18 (C10)** **TS- 19 (C11)**

Supplementary Figure 53. DFT-calculated structures of the transition structure of amide bond rotation of **17(C9), 18 (C10)** and **19 (C11)** in CH_2Cl_2.

Supplementary Table 1. The relative free energy ($\Delta\Delta G_{cis\text{-}trans}$) (kcal/mol) and the free energy of rotation from trans to cis ($\Delta G_{t\to c}^{\ddagger}$) of **17(C9)**, **18(C10)**, and **19(C11)** in CH_2Cl_2 at 25 °C, calculated at the level of M06-2X/6-311++G(d,p)(scrf = SMD, dichloromethane) // B3LYP/6-31G(d).

	$\Delta\Delta G_{cis\text{-}trans}$	$\Delta G_{t\to c}^{\ddagger}$
**17(C9)**	-0.4	9.6
**18(C10)**	1.4	12.7
**19(C11)**	1.2	13.6

In kcal/mol.

Supplementary Table 2. The relative free energy ($\Delta\Delta G_{cis\text{-}trans}$) and the free energy of rotation from cis to trans ($\Delta G_{c\rightarrow t}^{\ddagger}$) (kcal/mol) of **16(C8)**, **17(C9)** and **18(C10)** in methanol at 25 °C, calculated at the level of M06-2X/6-311++G(d)(scrf = SMD, methanol) // M06-2X/6-31G(d).

	$\Delta\Delta G_{cis\text{-}trans}$	$\Delta G_{c\rightarrow t}^{\ddagger}$
**16(C8)**	-1.7	14.5
**17(C9)**	-1.4	11.9
**18(C10)**	2.2	11.8

In kcal/mol.

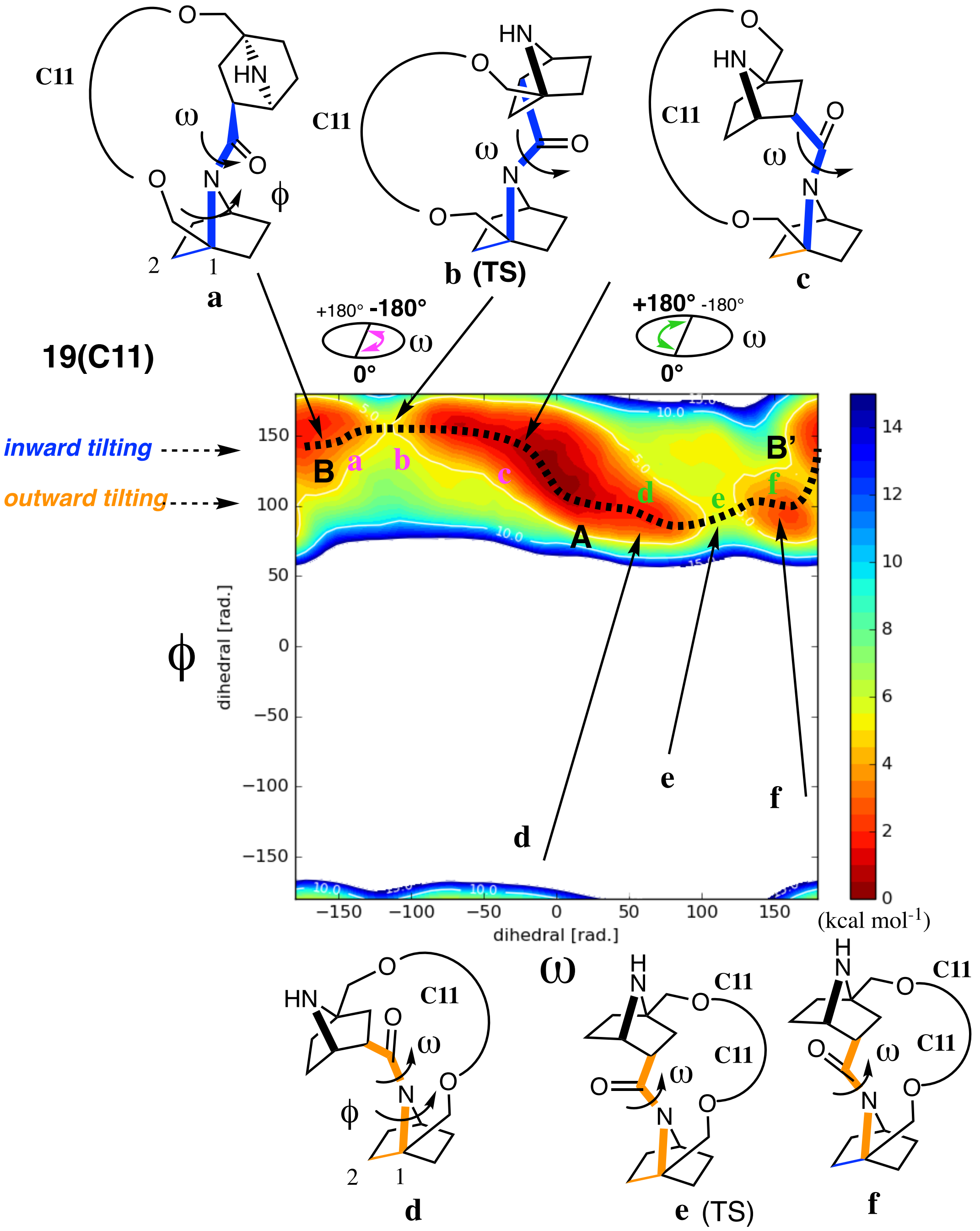

Supplementary Figure 54. The landscape of lactam amide rotation of bicycle lactam **19(C11)**. Metadymamic simulations in chloroform at 300K. Dashed lines indicate the rotational pathways. The tilting direction of the pyramidal nitrogen atom of the bicyclic systems is synchronized with the direction of the semicircle-rotation of the amide.

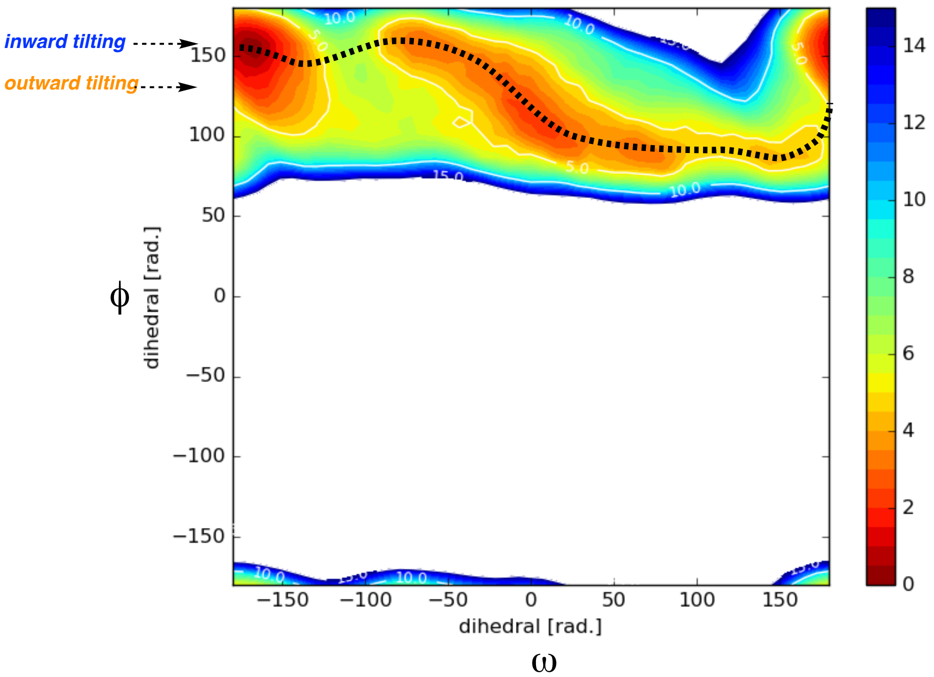

Supplementary Figure 55. Metadymamic simulations of the landscape of bicyclic lactam amide rotation. Bicycle lactam **16(C8)**.

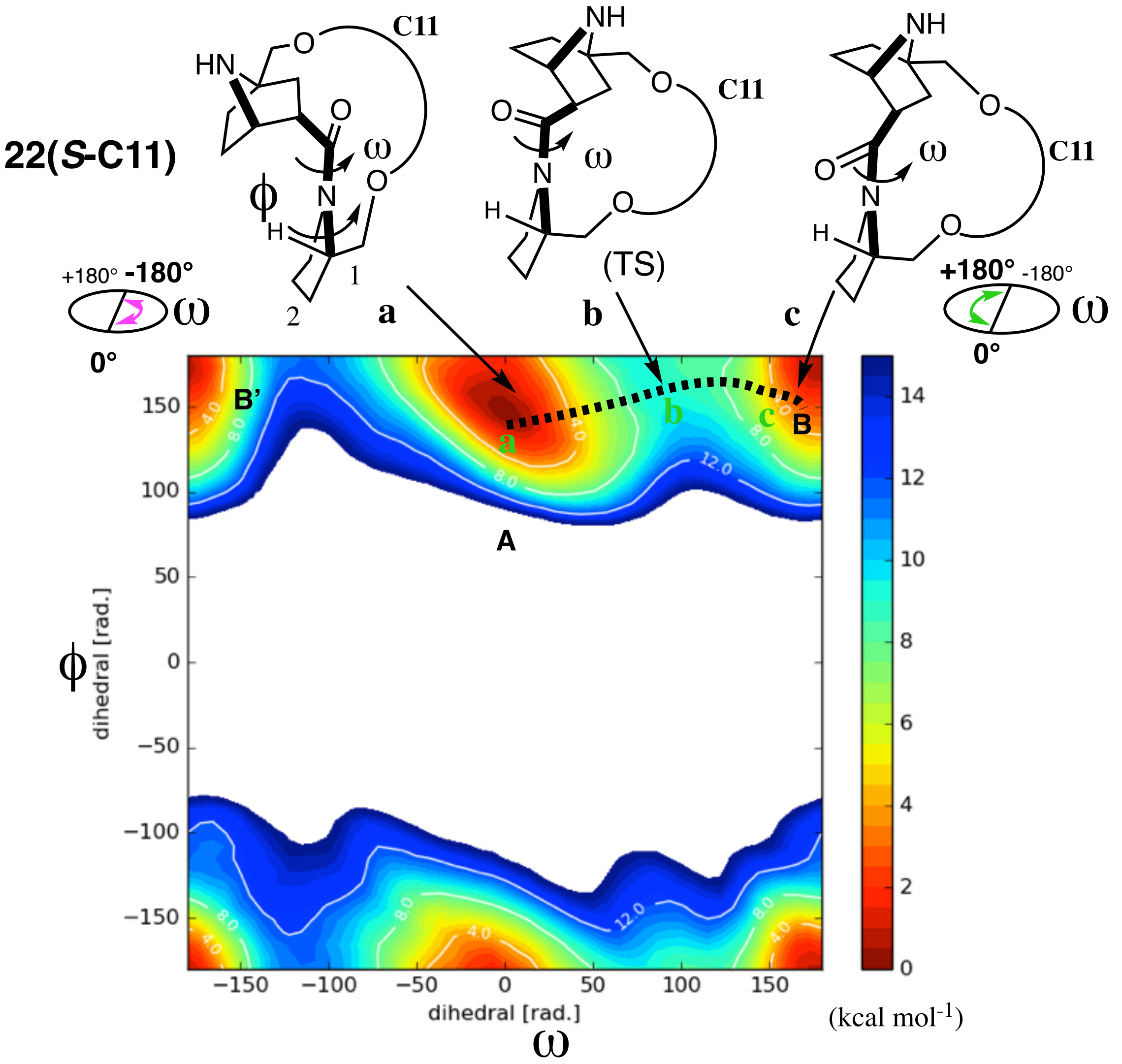

Supplementary Figure 56. The landscape of lactam amide rotation of monocycle lactam **22(*S*-C11)**. Metadymamic simulations in chloroform at 300K. Dashed lines indicate the rotational pathways. Rotation of the lactam amide bond is restricted to a south semicircle-rotation angle (e.g., 0° to +180°).

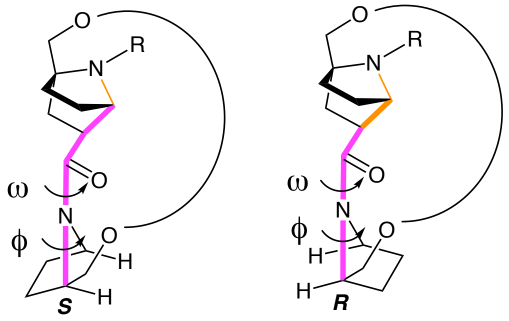

21(S-C9)
22(S-C11)

23(R-C9)
24(R-C11)

Supplementary Figure 57. Model reference planar amide lactams **21**-**24** for metadymamic simulations.

23 (*R*-C9)

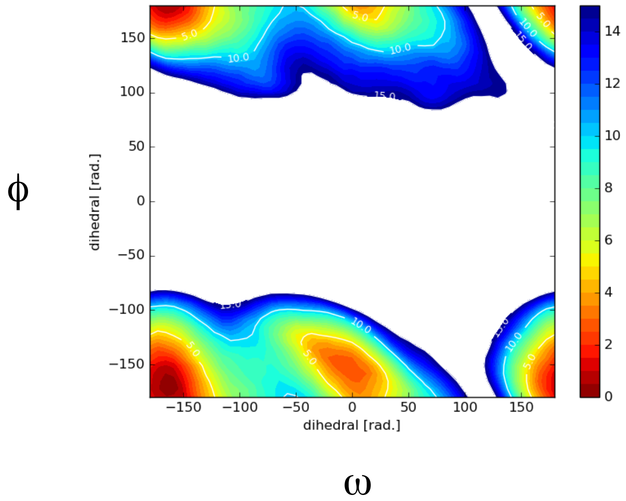

24 (*R*-C11)

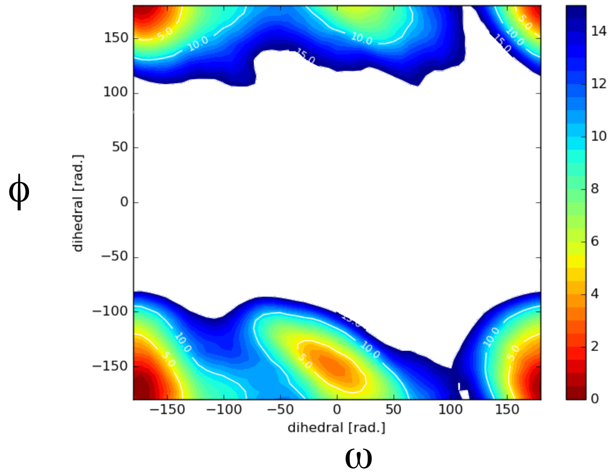

Supplementary Figure 58. Metadymamic simulations of *R*-isomers 23 and 24.

Supplementary References

1. Ammann, C., Meier, P. & Merbach, A. A simple multinuclear NMR thermometer. *J. Magn. Reson.* **46,** 319–321 (1982).

2. Hosoya, M., Otani, Y., Kawahata, M., Yamaguchi, K. & Ohwada, T. Water-stable helical structure of tertiary amides of bicyclic β-amino acid bearing 7-azabicyclo[2.2.1]heptane. Full control of amide cis-trans equilibrium by bridgehead substitution. *J. Am. Chem. Soc.* **132,** 14780–14789 (2010).

3. Frisch, M. J.; Trucks, G. W.; Schlegel, H. B.; Scuseria, G. E.; Robb, M. A.; Cheeseman, J. R.; Scalmani, G.; Barone, V.; Petersson, G. A.; Nakatsuji, H.; Li, X.; Caricato, M.; Marenich, A. V.; Bloino, J.; Janesko, B. G.; Gomperts, R.; Mennucci, B.; Hratchian, H. P.; Ortiz, J. V.; Izmaylov, A. F.; Sonnenberg, J. L.; Williams-Young, D.; Ding, F.; Lipparini, F.; Egidi, F.; Goings, J.; Peng, B.; Petrone, A.; Henderson, T.; Ranasinghe, D.; Zakrzewski, V. G.; Gao, J.; Rega, N.; Zheng, G.; Liang, W.; Hada, M.; Ehara, M.; Toyota, K.; Fukuda, R.; Hasegawa, J.; Ishida, M.; Nakajima, T.; Honda, Y.; Kitao, O.; Nakai, H.; Vreven, T.; Throssell, K.; Montgomery, J. A., Jr.; Peralta, J. E.; Ogliaro, F.; Bearpark, M. J.; Heyd, J. J.; Brothers, E. N.; Kudin, K. N.; Staroverov, V. N.; Keith, T. A.; Kobayashi, R.; Normand, J.; Raghavachari, K.; Rendell, A. P.; Burant, J. C.; Iyengar, S. S.; Tomasi, J.; Cossi, M.; Millam, J. M.; Klene, M.; Adamo, C.; Cammi, R.; Ochterski, J. W.; Martin, R. L.; Morokuma, K.; Farkas, O.; Foresman, J. B.; Fox, D. J. Gaussian, Inc., Wallingford CT, Gaussian 16, Revision B.01, (2016).
