# Supplementary material for: Amide nitrogen pyramidalization changes lactam amide spinning
Source: Nat Commun. 2019 Jan 28;10:461. doi: 10.1038/s41467-018-08249-9 (PMC6349922; doi:10.1038/s41467-018-08249-9)
Supplement: Supplementary file 2 — Supplementary Data 1 [file 41467_2018_8249_MOESM2_ESM.pdf]

**Supplementary Data**

**Amide nitrogen pyramidalization changes lactam amide spinning**

Yuko Otani *et al*

[1] *Graduate School of Pharmaceutical Sciences, University of Tokyo, 7-3-1 Hongo, Bunkyo-ku, Tokyo, 113-0033, Japan*

[2] *Department of Pharmaceutical Sciences at Kagawa Campus, Tokushima Bunri University, 1314-1 Shido, Sanuki, Kagawa 769-2193, Japan.*

*E-mail: otani@ mol.f.u-tokyo.ac.jp, ohwada@mol.f.u-tokyo.ac.jp*

## Coordinates of DFT-optimized structures

### 16(C8)-*cis*

Energy at the level of M06-2X/6-311++G(d,p) (SCRF = SMD, methanol) // M06-2X/6-31G(d): -1234.341525

Number of imaginary frequency: 0

Correction for Gibbs free energy at 25 °C (M06-2X/6-311++G(d,p), SCRF = SMD, methanol): 0.544504

Standard orientation (M06-2X/6-31G(d))

| Center Number | Atomic Number | Atomic Type | Coordinates (Angstroms) X | Y | Z |
|---|---|---|---|---|---|
| 1 | 6 | 0 | 0.316084 | -0.787723 | -0.151100 |
| 2 | 6 | 0 | -0.866204 | -1.314203 | -1.013134 |
| 3 | 6 | 0 | -1.671868 | -2.155488 | 0.003800 |
| 4 | 6 | 0 | -0.884514 | -3.447588 | 0.296844 |
| 5 | 6 | 0 | 0.240130 | -2.936257 | 1.237329 |
| 6 | 6 | 0 | -0.026261 | -1.423460 | 1.243635 |
| 7 | 6 | 0 | -3.133037 | -2.353856 | -0.325355 |
| 8 | 6 | 0 | 1.655602 | -1.215010 | -0.724253 |
| 9 | 6 | 0 | 4.555699 | -1.372307 | 0.353224 |
| 10 | 6 | 0 | 3.934319 | -0.345530 | 1.339794 |
| 11 | 6 | 0 | 3.150114 | 0.601467 | 0.395346 |
| 12 | 6 | 0 | 4.169230 | 1.453707 | -0.391511 |

| | | | | | |
|---|---|---|---|---|---|
| 13 | 6 | 0 | 4.794118 | 0.435545 | -1.377377 |
| 14 | 6 | 0 | 4.055281 | -0.858620 | -1.013043 |
| 15 | 6 | 0 | 2.135391 | 1.450806 | 1.126702 |
| 16 | 6 | 0 | 0.697326 | 3.282503 | 0.901529 |
| 17 | 7 | 0 | 2.717298 | -0.341209 | -0.668238 |
| 18 | 8 | 0 | -3.712806 | -1.081504 | -0.525012 |
| 19 | 8 | 0 | 1.776189 | -2.299093 | -1.282600 |
| 20 | 8 | 0 | 1.430371 | 2.274184 | 0.233976 |
| 21 | 1 | 0 | 0.276921 | 0.302416 | -0.074767 |
| 22 | 1 | 0 | -0.517756 | -1.902363 | -1.864274 |
| 23 | 1 | 0 | -1.476563 | -0.483185 | -1.378469 |
| 24 | 1 | 0 | -0.484757 | -3.888270 | -0.619180 |
| 25 | 1 | 0 | -1.520522 | -4.183117 | 0.799520 |
| 26 | 1 | 0 | 0.104479 | -3.320459 | 2.250960 |
| 27 | 1 | 0 | 1.237327 | -3.201850 | 0.885575 |
| 28 | 1 | 0 | 0.412838 | -0.901510 | 2.097553 |
| 29 | 1 | 0 | -3.256554 | -2.978376 | -1.223152 |
| 30 | 1 | 0 | -3.613400 | -2.864570 | 0.522147 |
| 31 | 1 | 0 | 5.646946 | -1.412181 | 0.408318 |
| 32 | 1 | 0 | 3.243199 | -0.833139 | 2.037807 |
| 33 | 1 | 0 | 4.679776 | 0.196996 | 1.929077 |
| 34 | 1 | 0 | 4.898170 | 1.907577 | 0.286872 |
| 35 | 1 | 0 | 3.638969 | 2.251384 | -0.915564 |
| 36 | 1 | 0 | 4.589320 | 0.715131 | -2.413220 |
| 37 | 1 | 0 | 5.875714 | 0.329415 | -1.258920 |
| 38 | 1 | 0 | 4.001701 | -1.613361 | -1.793344 |
| 39 | 1 | 0 | 1.441193 | 0.836606 | 1.717402 |
| 40 | 1 | 0 | 2.706730 | 2.066770 | 1.842434 |
| 41 | 1 | 0 | 0.061800 | 2.831331 | 1.680499 |
| 42 | 1 | 0 | 4.165906 | -2.377647 | 0.533606 |
| 43 | 1 | 0 | 0.532416 | 4.441748 | -0.876832 |
| 44 | 1 | 0 | 1.394257 | 3.969884 | 1.406672 |
| 45 | 6 | 0 | -0.142996 | 4.028007 | -0.119523 |
| 46 | 1 | 0 | -0.620461 | 4.877348 | 0.385690 |
| 47 | 6 | 0 | -1.210479 | 3.161040 | -0.790547 |
| 48 | 1 | 0 | -0.726302 | 2.318801 | -1.301267 |
| 49 | 6 | 0 | -2.274899 | 2.632973 | 0.172367 |
| 50 | 1 | 0 | -1.703518 | 3.755650 | -1.569877 |
| 51 | 1 | 0 | -1.820159 | 1.923604 | 0.880130 |
| 52 | 1 | 0 | -2.666651 | 3.462137 | 0.780541 |
| 53 | 6 | 0 | -5.501948 | 0.471213 | -0.291677 |
| 54 | 1 | 0 | -6.507583 | 0.559853 | 0.134289 |
| 55 | 1 | 0 | -5.584206 | 0.725537 | -1.355549 |
| 56 | 7 | 0 | -1.496891 | -1.425876 | 1.279296 |
| 57 | 1 | 0 | -1.864421 | -0.478303 | 1.157544 |
| 58 | 6 | 0 | -3.423888 | 1.933618 | -0.548775 |
| 59 | 1 | 0 | -3.032420 | 1.084732 | -1.118575 |
| 60 | 1 | 0 | -3.867422 | 2.623324 | -1.280662 |
| 61 | 6 | 0 | -4.523553 | 1.437740 | 0.394393 |
| 62 | 1 | 0 | -4.067241 | 0.930918 | 1.256793 |
| 63 | 1 | 0 | -5.064100 | 2.302747 | 0.797237 |
| 64 | 6 | 0 | -5.066532 | -0.980689 | -0.147774 |
| 65 | 1 | 0 | -5.175037 | -1.297923 | 0.902582 |
| 66 | 1 | 0 | -5.694167 | -1.644077 | -0.763265 |

---------------------------------------------------------------------

## 16(C8)-TS

Energy at the level of M06-2X/6-311++G(d,p) (SCRF = SMD, methanol) // M06-2X/6-31G(d): -1234.319596

Number of imaginary frequency: 1

Correction for Gibbs free energy at 25 °C (M06-2X/6-311++G(d,p), SCRF = SMD, methanol): 0.545721

Standard orientation (M06-2X/6-31G(d))

| Center Number | Atomic Number | Atomic Type | Coordinates (Angstroms) X | Y | Z |
|---|---|---|---|---|---|
| 1 | 6 | 0 | -0.060687 | -0.843033 | -0.552772 |
| 2 | 6 | 0 | 0.628929 | -1.306761 | 0.763633 |
| 3 | 6 | 0 | 1.874639 | -2.054130 | 0.215090 |
| 4 | 6 | 0 | 1.409563 | -3.420987 | -0.316461 |
| 5 | 6 | 0 | 0.664711 | -3.023024 | -1.621321 |
| 6 | 6 | 0 | 0.831024 | -1.492872 | -1.633407 |
| 7 | 6 | 0 | 3.062230 | -2.095657 | 1.159489 |
| 8 | 6 | 0 | -1.542606 | -1.096044 | -0.641807 |
| 9 | 6 | 0 | -4.312916 | -1.782494 | 0.292202 |
| 10 | 6 | 0 | -4.485308 | -0.471515 | -0.515320 |
| 11 | 6 | 0 | -3.358423 | 0.423505 | 0.060870 |
| 12 | 6 | 0 | -3.786671 | 0.883373 | 1.466853 |
| 13 | 6 | 0 | -3.649733 | -0.425365 | 2.294684 |
| 14 | 6 | 0 | -3.144481 | -1.425143 | 1.237454 |
| 15 | 6 | 0 | -2.895471 | 1.512154 | -0.880517 |
| 16 | 6 | 0 | -1.276641 | 3.202086 | -1.129162 |
| 17 | 7 | 0 | -2.269871 | -0.518030 | 0.453517 |
| 18 | 8 | 0 | 3.635363 | -0.810498 | 1.311274 |
| 19 | 8 | 0 | -2.064035 | -1.651244 | -1.585136 |
| 20 | 8 | 0 | -1.875746 | 2.259172 | -0.267715 |
| 21 | 1 | 0 | 0.017500 | 0.255416 | -0.613858 |
| 22 | 1 | 0 | -0.007960 | -1.958592 | 1.371520 |
| 23 | 1 | 0 | 0.918477 | -0.455763 | 1.386596 |
| 24 | 1 | 0 | 0.773117 | -3.944917 | 0.402438 |
| 25 | 1 | 0 | 2.273859 | -4.055472 | -0.534271 |
| 26 | 1 | 0 | 1.162215 | -3.450004 | -2.494782 |
| 27 | 1 | 0 | -0.381777 | -3.325678 | -1.642151 |
| 28 | 1 | 0 | 0.738555 | -1.038548 | -2.621540 |
| 29 | 1 | 0 | 2.739429 | -2.440050 | 2.149582 |
| 30 | 1 | 0 | 3.811177 | -2.805398 | 0.781292 |
| 31 | 1 | 0 | -5.208845 | -2.064041 | 0.853181 |
| 32 | 1 | 0 | -4.336654 | -0.649912 | -1.582831 |
| 33 | 1 | 0 | -5.464872 | -0.005005 | -0.373466 |
| 34 | 1 | 0 | -4.803400 | 1.286905 | 1.462883 |
| 35 | 1 | 0 | -3.101601 | 1.654920 | 1.822014 |
| 36 | 1 | 0 | -2.912880 | -0.307746 | 3.091968 |
| 37 | 1 | 0 | -4.592753 | -0.753774 | 2.740235 |
| 38 | 1 | 0 | -2.599709 | -2.281190 | 1.643169 |
| 39 | 1 | 0 | -2.534978 | 1.059132 | -1.820378 |
| 40 | 1 | 0 | -3.750755 | 2.159270 | -1.138001 |
| 41 | 1 | 0 | -0.705069 | 2.679640 | -1.914881 |
| 42 | 1 | 0 | -4.043430 | -2.608082 | -0.368687 |
| 43 | 1 | 0 | -1.025807 | 4.633290 | 0.419978 |
| 44 | 1 | 0 | -2.051068 | 3.802683 | -1.632288 |
| 45 | 6 | 0 | -0.382397 | 4.098563 | -0.287603 |
| 46 | 1 | 0 | 0.077947 | 4.852095 | -0.939403 |
| 47 | 6 | 0 | 0.696695 | 3.332970 | 0.480951 |
| 48 | 1 | 0 | 0.227086 | 2.490223 | 1.004318 |
| 49 | 6 | 0 | 1.850357 | 2.825547 | -0.383842 |
| 50 | 1 | 0 | 1.112387 | 3.988977 | 1.255921 |
| 51 | 1 | 0 | 1.473704 | 2.165494 | -1.180411 |
| 52 | 1 | 0 | 2.325945 | 3.676045 | -0.893603 |
| 53 | 6 | 0 | 5.151450 | 0.854759 | 0.455055 |
| 54 | 1 | 0 | 6.140856 | 0.928390 | -0.011948 |
| 55 | 1 | 0 | 5.250240 | 1.252692 | 1.472879 |
| 56 | 7 | 0 | 2.186922 | -1.368843 | -1.061131 |
| 57 | 1 | 0 | 2.386496 | -0.385831 | -0.855401 |
| 58 | 6 | 0 | 2.889045 | 2.073099 | 0.445484 |
| 59 | 1 | 0 | 2.436910 | 1.174130 | 0.878436 |

| | | | | | |
|---|---|---|---|---|---|
| 60 | 1 | 0 | 3.188649 | 2.697551 | 1.299038 |
| 61 | 6 | 0 | 4.144768 | 1.694677 | -0.341277 |
| 62 | 1 | 0 | 3.870336 | 1.150224 | -1.257600 |
| 63 | 1 | 0 | 4.631427 | 2.618359 | -0.679935 |
| 64 | 6 | 0 | 4.807502 | -0.623908 | 0.531464 |
| 65 | 1 | 0 | 4.649278 | -1.021895 | -0.481066 |
| 66 | 1 | 0 | 5.638474 | -1.183667 | 0.988537 |

## 16(C8)-*trans*

Energy at the level of M06-2X/6-311++G(d,p) (SCRF = SMD, methanol) // M06-2X/6-31G(d): -1234.339704

Number of imaginary frequency: 0

Correction for Gibbs free energy at 25 °C (M06-2X/6-311++G(d,p), SCRF = SMD, methanol): 0.545359

Standard orientation (M06-2X/6-31G(d))

| Center Number | Atomic Number | Atomic Type | Coordinates (Angstroms) X | Y | Z |
|---|---|---|---|---|---|
| 1 | 6 | 0 | -0.546554 | -1.944301 | -0.655398 |
| 2 | 6 | 0 | 0.931750 | -1.862823 | -1.106173 |
| 3 | 6 | 0 | 1.678357 | -2.024728 | 0.233550 |
| 4 | 6 | 0 | 1.413302 | -0.774398 | 1.100781 |
| 5 | 6 | 0 | -0.015455 | -1.025787 | 1.642455 |
| 6 | 6 | 0 | -0.396077 | -2.296264 | 0.868658 |
| 7 | 6 | 0 | 3.151205 | -2.342915 | 0.109814 |
| 8 | 6 | 0 | -1.296017 | -0.633047 | -0.903447 |
| 9 | 6 | 0 | -4.598155 | -1.406909 | -1.042829 |
| 10 | 6 | 0 | -4.574628 | 0.135457 | -1.243454 |
| 11 | 6 | 0 | -3.461228 | 0.580871 | -0.269171 |
| 12 | 6 | 0 | -3.988950 | 0.404666 | 1.176790 |
| 13 | 6 | 0 | -3.979503 | -1.135008 | 1.376770 |
| 14 | 6 | 0 | -3.481522 | -1.626210 | 0.006147 |
| 15 | 6 | 0 | -2.881548 | 1.950182 | -0.544497 |
| 16 | 6 | 0 | -1.319491 | 3.519499 | 0.241160 |
| 17 | 7 | 0 | -2.536866 | -0.564219 | -0.349560 |
| 18 | 8 | 0 | 3.739043 | -1.216411 | -0.497579 |
| 19 | 8 | 0 | -0.778337 | 0.312159 | -1.480746 |
| 20 | 8 | 0 | -1.845835 | 2.211743 | 0.367545 |
| 21 | 1 | 0 | -1.076370 | -2.754929 | -1.171008 |
| 22 | 1 | 0 | 1.147713 | -0.923424 | -1.617100 |
| 23 | 1 | 0 | 1.191186 | -2.691380 | -1.775843 |
| 24 | 1 | 0 | 1.473594 | 0.132149 | 0.498846 |
| 25 | 1 | 0 | 2.151859 | -0.702680 | 1.905985 |
| 26 | 1 | 0 | -0.000080 | -1.247891 | 2.712198 |
| 27 | 1 | 0 | -0.692208 | -0.185032 | 1.467621 |
| 28 | 1 | 0 | -1.233996 | -2.855777 | 1.291944 |
| 29 | 1 | 0 | 3.572442 | -2.512655 | 1.112816 |
| 30 | 1 | 0 | 3.323453 | -3.251185 | -0.492520 |
| 31 | 1 | 0 | -5.564043 | -1.779477 | -0.690749 |
| 32 | 1 | 0 | -4.298798 | 0.394989 | -2.270375 |
| 33 | 1 | 0 | -5.531566 | 0.612766 | -1.014626 |
| 34 | 1 | 0 | -4.983842 | 0.846277 | 1.287154 |
| 35 | 1 | 0 | -3.308120 | 0.904761 | 1.868601 |
| 36 | 1 | 0 | -3.274604 | -1.424334 | 2.161576 |
| 37 | 1 | 0 | -4.960967 | -1.546506 | 1.626923 |
| 38 | 1 | 0 | -3.042469 | -2.623043 | -0.001128 |
| 39 | 1 | 0 | -2.507653 | 2.007124 | -1.573715 |

| | | | | | |
|---|---|---|---|---|---|
| 40 | 1 | 0 | -3.699470 | 2.683053 | -0.425085 |
| 41 | 1 | 0 | -1.307679 | 3.815010 | -0.819937 |
| 42 | 1 | 0 | -4.352808 | -1.926293 | -1.972935 |
| 43 | 1 | 0 | 0.091640 | 3.030186 | 1.764355 |
| 44 | 1 | 0 | -1.968400 | 4.229214 | 0.778040 |
| 45 | 6 | 0 | 0.099550 | 3.532277 | 0.788001 |
| 46 | 1 | 0 | 0.409037 | 4.570273 | 0.964527 |
| 47 | 6 | 0 | 1.077894 | 2.834274 | -0.156190 |
| 48 | 1 | 0 | 1.173024 | 3.426476 | -1.077603 |
| 49 | 6 | 0 | 2.462410 | 2.610073 | 0.449915 |
| 50 | 1 | 0 | 0.641634 | 1.873483 | -0.458031 |
| 51 | 1 | 0 | 2.915180 | 3.572236 | 0.729878 |
| 52 | 1 | 0 | 2.351901 | 2.043236 | 1.385736 |
| 53 | 6 | 0 | 5.501521 | 0.374986 | -0.634323 |
| 54 | 1 | 0 | 5.344046 | 0.549012 | -1.705947 |
| 55 | 1 | 0 | 6.576466 | 0.484853 | -0.449700 |
| 56 | 7 | 0 | 0.878294 | -3.038535 | 0.956246 |
| 57 | 1 | 0 | 0.820947 | -3.889907 | 0.391652 |
| 58 | 6 | 0 | 3.396342 | 1.842531 | -0.487407 |
| 59 | 1 | 0 | 3.632775 | 2.463861 | -1.362501 |
| 60 | 1 | 0 | 2.878156 | 0.957158 | -0.870817 |
| 61 | 6 | 0 | 4.697487 | 1.397621 | 0.186931 |
| 62 | 1 | 0 | 5.314590 | 2.280057 | 0.396546 |
| 63 | 1 | 0 | 4.457137 | 0.952840 | 1.163680 |
| 64 | 6 | 0 | 5.124129 | -1.061846 | -0.298224 |
| 65 | 1 | 0 | 5.689446 | -1.777954 | -0.915640 |
| 66 | 1 | 0 | 5.368958 | -1.266402 | 0.757465 |

## 17(C9)-*cis*

Energy at the level of M06-2X/6-311++G(d,p) (SCRF = SMD, methanol) // M06-2X/6-31G(d): -1273.64915

Number of imaginary frequency: 0

Correction for Gibbs free energy at 25 °C (M06-2X/6-311++G(d,p), SCRF = SMD, methanol): 0.572956

Standard orientation (M06-2X/6-31G(d))

| Center Number | Atomic Number | Atomic Type | Coordinates (Angstroms) X | Y | Z |
|---|---|---|---|---|---|
| 1 | 6 | 0 | 0.514965 | -0.751500 | -0.127091 |
| 2 | 6 | 0 | -0.687136 | -1.298877 | -0.946756 |
| 3 | 6 | 0 | -1.439982 | -2.152154 | 0.098067 |
| 4 | 6 | 0 | -0.603125 | -3.413217 | 0.398514 |
| 5 | 6 | 0 | 0.509110 | -2.856052 | 1.325786 |
| 6 | 6 | 0 | 0.205547 | -1.351439 | 1.293828 |
| 7 | 6 | 0 | -2.889508 | -2.433880 | -0.212155 |
| 8 | 6 | 0 | 1.839617 | -1.204197 | -0.717634 |
| 9 | 6 | 0 | 4.746579 | -1.382666 | 0.362356 |
| 10 | 6 | 0 | 4.143123 | -0.320871 | 1.323165 |
| 11 | 6 | 0 | 3.372113 | 0.611793 | 0.354868 |
| 12 | 6 | 0 | 4.403241 | 1.423662 | -0.459718 |
| 13 | 6 | 0 | 5.003851 | 0.369118 | -1.421949 |
| 14 | 6 | 0 | 4.246419 | -0.901540 | -1.016240 |
| 15 | 6 | 0 | 2.373217 | 1.502113 | 1.058251 |
| 16 | 6 | 0 | 0.968189 | 3.349156 | 0.753923 |
| 17 | 7 | 0 | 2.919102 | -0.352705 | -0.679004 |
| 18 | 8 | 0 | -3.551403 | -1.211132 | -0.442450 |
| 19 | 8 | 0 | 1.934525 | -2.294346 | -1.269413 |

| | | | | | |
|---|---|---|---|---|---|
| 20 | 8 | 0 | 1.666783 | 2.287990 | 0.133424 |
| 21 | 1 | 0 | 0.484447 | 0.340285 | -0.081739 |
| 22 | 1 | 0 | -0.360480 | -1.878579 | -1.812245 |
| 23 | 1 | 0 | -1.329284 | -0.479317 | -1.284725 |
| 24 | 1 | 0 | -0.194086 | -3.845754 | -0.517115 |
| 25 | 1 | 0 | -1.209878 | -4.166309 | 0.911221 |
| 26 | 1 | 0 | 0.381737 | -3.217538 | 2.348761 |
| 27 | 1 | 0 | 1.512197 | -3.108883 | 0.980671 |
| 28 | 1 | 0 | 0.642646 | -0.792514 | 2.124921 |
| 29 | 1 | 0 | -2.973081 | -3.086484 | -1.097135 |
| 30 | 1 | 0 | -3.341746 | -2.957300 | 0.644841 |
| 31 | 1 | 0 | 5.837522 | -1.434710 | 0.413391 |
| 32 | 1 | 0 | 3.445694 | -0.779708 | 2.034289 |
| 33 | 1 | 0 | 4.898135 | 0.224907 | 1.897055 |
| 34 | 1 | 0 | 5.144187 | 1.881063 | 0.203114 |
| 35 | 1 | 0 | 3.884903 | 2.217247 | -1.001749 |
| 36 | 1 | 0 | 4.795033 | 0.623337 | -2.463515 |
| 37 | 1 | 0 | 6.084504 | 0.248543 | -1.308858 |
| 38 | 1 | 0 | 4.175723 | -1.677805 | -1.773643 |
| 39 | 1 | 0 | 1.678498 | 0.922686 | 1.682567 |
| 40 | 1 | 0 | 2.956994 | 2.145016 | 1.739222 |
| 41 | 1 | 0 | 0.376257 | 2.965457 | 1.600439 |
| 42 | 1 | 0 | 4.345908 | -2.377787 | 0.573381 |
| 43 | 1 | 0 | 0.691878 | 4.363044 | -1.102001 |
| 44 | 1 | 0 | 1.688905 | 4.075633 | 1.161287 |
| 45 | 6 | 0 | 0.067035 | 4.003736 | -0.276598 |
| 46 | 1 | 0 | -0.393874 | 4.885064 | 0.187585 |
| 47 | 6 | 0 | -1.027151 | 3.076537 | -0.810691 |
| 48 | 1 | 0 | -0.568163 | 2.237712 | -1.348819 |
| 49 | 6 | 0 | -1.961303 | 2.541757 | 0.277407 |
| 50 | 1 | 0 | -1.621482 | 3.631368 | -1.547675 |
| 51 | 1 | 0 | -1.441324 | 1.772161 | 0.868624 |
| 52 | 1 | 0 | -2.206889 | 3.350213 | 0.982387 |
| 53 | 6 | 0 | -5.572266 | -0.006804 | -0.885644 |
| 54 | 6 | 0 | -5.564299 | 0.999520 | 0.270394 |
| 55 | 1 | 0 | -6.155448 | 0.587273 | 1.098664 |
| 56 | 1 | 0 | -6.090541 | 1.903852 | -0.062535 |
| 57 | 1 | 0 | -5.058382 | 0.405889 | -1.761760 |
| 58 | 1 | 0 | -6.611583 | -0.182923 | -1.188043 |
| 59 | 7 | 0 | -1.264783 | -1.392339 | 1.355094 |
| 60 | 1 | 0 | -1.656753 | -0.455977 | 1.224913 |
| 61 | 6 | 0 | -3.254913 | 1.955857 | -0.285040 |
| 62 | 1 | 0 | -3.012279 | 1.158868 | -0.998018 |
| 63 | 1 | 0 | -3.781024 | 2.738260 | -0.852317 |
| 64 | 6 | 0 | -4.181994 | 1.403157 | 0.796057 |
| 65 | 1 | 0 | -3.706197 | 0.541765 | 1.278637 |
| 66 | 1 | 0 | -4.319599 | 2.165646 | 1.574808 |
| 67 | 6 | 0 | -4.949406 | -1.355076 | -0.561804 |
| 68 | 1 | 0 | -5.366164 | -1.756917 | 0.377748 |
| 69 | 1 | 0 | -5.177932 | -2.080385 | -1.359523 |

---------------------------------------------------------------------

## 17(C9)-TS

Energy at the level of M06-2X/6-311++G(d,p) (SCRF = SMD, methanol) // M06-2X/6-31G(d): -1273.628531

Number of imaginary frequency: 1

Correction for Gibbs free energy at 25 °C (M06-2X/6-311++G(d,p), SCRF = SMD, methanol): 0.571277

Standard orientation (M06-2X/6-31G(d))

| Center Number | Atomic Number | Atomic Type | Coordinates (Angstroms) X | Y | Z |
|---|---|---|---|---|---|
| 1 | 6 | 0 | 0.294379 | -0.849087 | -0.167951 |
| 2 | 6 | 0 | -0.793558 | -1.410152 | -1.104645 |
| 3 | 6 | 0 | -1.592666 | -2.341309 | -0.153095 |
| 4 | 6 | 0 | -0.773583 | -3.624982 | 0.057045 |
| 5 | 6 | 0 | 0.388156 | -3.112237 | 0.955409 |
| 6 | 6 | 0 | 0.014504 | -1.629318 | 1.154001 |
| 7 | 6 | 0 | -3.043042 | -2.547701 | -0.515205 |
| 8 | 6 | 0 | 1.722425 | -0.964813 | -0.624644 |
| 9 | 6 | 0 | 4.290444 | -0.195838 | 1.898981 |
| 10 | 6 | 0 | 4.244040 | 1.102288 | 1.045981 |
| 11 | 6 | 0 | 3.605451 | 0.605657 | -0.264010 |
| 12 | 6 | 0 | 4.646503 | -0.273233 | -1.004843 |
| 13 | 6 | 0 | 4.631469 | -1.578371 | -0.168475 |
| 14 | 6 | 0 | 3.635955 | -1.225348 | 0.957579 |
| 15 | 6 | 0 | 2.963181 | 1.668848 | -1.125482 |
| 16 | 6 | 0 | 1.290815 | 3.307060 | -1.158024 |
| 17 | 7 | 0 | 2.624082 | -0.351847 | 0.313763 |
| 18 | 8 | 0 | -3.656366 | -1.278713 | -0.570080 |
| 19 | 8 | 0 | 2.067957 | -1.481220 | -1.664828 |
| 20 | 8 | 0 | 1.939227 | 2.308369 | -0.402499 |
| 21 | 1 | 0 | 0.151604 | 0.218465 | 0.038181 |
| 22 | 1 | 0 | -0.359610 | -1.944840 | -1.954592 |
| 23 | 1 | 0 | -1.438790 | -0.614705 | -1.489031 |
| 24 | 1 | 0 | -0.427514 | -4.051030 | -0.888064 |
| 25 | 1 | 0 | -1.371919 | -4.375819 | 0.581861 |
| 26 | 1 | 0 | 0.401162 | -3.629377 | 1.917312 |
| 27 | 1 | 0 | 1.366795 | -3.242723 | 0.483798 |
| 28 | 1 | 0 | 0.446897 | -1.159482 | 2.038859 |
| 29 | 1 | 0 | -3.140676 | -3.067623 | -1.481872 |
| 30 | 1 | 0 | -3.518584 | -3.166543 | 0.261389 |
| 31 | 1 | 0 | 5.305878 | -0.493393 | 2.175033 |
| 32 | 1 | 0 | 3.598275 | 1.856508 | 1.499815 |
| 33 | 1 | 0 | 5.231437 | 1.539362 | 0.870418 |
| 34 | 1 | 0 | 5.627408 | 0.211409 | -1.025468 |
| 35 | 1 | 0 | 4.331589 | -0.467617 | -2.032579 |
| 36 | 1 | 0 | 4.260349 | -2.412404 | -0.767840 |
| 37 | 1 | 0 | 5.613543 | -1.847673 | 0.231018 |
| 38 | 1 | 0 | 3.187265 | -2.081693 | 1.466788 |
| 39 | 1 | 0 | 3.733258 | 2.394712 | -1.437415 |
| 40 | 1 | 0 | 2.554080 | 1.209548 | -2.041079 |
| 41 | 1 | 0 | 2.016628 | 4.076631 | -1.467317 |
| 42 | 1 | 0 | 3.702999 | -0.085800 | 2.813117 |
| 43 | 1 | 0 | -0.295576 | 4.709209 | -0.910972 |
| 44 | 1 | 0 | 0.868647 | 2.863616 | -2.076167 |
| 45 | 6 | 0 | 0.183548 | 3.918837 | -0.318541 |
| 46 | 1 | 0 | 0.626544 | 4.401960 | 0.560826 |
| 47 | 6 | 0 | -0.863715 | 2.897236 | 0.122415 |
| 48 | 1 | 0 | -0.396939 | 2.181073 | 0.810544 |
| 49 | 6 | 0 | -2.068086 | 3.547916 | 0.801373 |
| 50 | 1 | 0 | -1.192701 | 2.322997 | -0.755441 |
| 51 | 1 | 0 | -1.716911 | 4.141340 | 1.655669 |
| 52 | 1 | 0 | -2.533080 | 4.258332 | 0.102984 |
| 53 | 6 | 0 | -5.604791 | 0.095147 | -0.531291 |
| 54 | 6 | 0 | -5.002247 | 0.968402 | 0.580706 |
| 55 | 1 | 0 | -4.759456 | 0.337922 | 1.447822 |
| 56 | 1 | 0 | -5.749482 | 1.693310 | 0.926758 |
| 57 | 1 | 0 | -5.410599 | 0.555768 | -1.507802 |
| 58 | 1 | 0 | -6.693517 | 0.030054 | -0.423629 |
| 59 | 7 | 0 | -1.450478 | -1.726658 | 1.186065 |
| 60 | 1 | 0 | -1.875355 | -0.796162 | 1.169299 |
| 61 | 6 | 0 | -3.130165 | 2.552723 | 1.275381 |

| | | | | | |
|---|---|---|---|---|---|
| 62 | 1 | 0 | -3.924532 | 3.103632 | 1.796528 |
| 63 | 1 | 0 | -2.691660 | 1.872143 | 2.019386 |
| 64 | 6 | 0 | -3.752375 | 1.734848 | 0.142701 |
| 65 | 1 | 0 | -4.023658 | 2.410847 | -0.681986 |
| 66 | 1 | 0 | -3.024607 | 1.024677 | -0.265411 |
| 67 | 6 | 0 | -5.063675 | -1.325400 | -0.531006 |
| 68 | 1 | 0 | -5.386115 | -1.848228 | 0.384924 |
| 69 | 1 | 0 | -5.452652 | -1.893399 | -1.391362 |

## 17(C9)-*trans*

Energy at the level of M06-2X/6-311++G(d,p) (SCRF = SMD, methanol) // M06-2X/6-31G(d): -1273.64736

Number of imaginary frequency: 0

Correction for Gibbs free energy at 25 °C (M06-2X/6-311++G(d,p), SCRF = SMD, methanol): 0.573464

| Center Number | Atomic Number | Atomic Type | Coordinates (Angstroms) X | Y | Z |
|---|---|---|---|---|---|
| 1 | 6 | 0 | -0.108287 | -1.133397 | 0.425573 |
| 2 | 6 | 0 | -1.433615 | -0.650088 | -0.206425 |
| 3 | 6 | 0 | -2.152980 | -1.977241 | -0.544125 |
| 4 | 6 | 0 | -1.457848 | -2.596716 | -1.770973 |
| 5 | 6 | 0 | -0.128560 | -3.119984 | -1.158092 |
| 6 | 6 | 0 | -0.269787 | -2.687714 | 0.311160 |
| 7 | 6 | 0 | -3.656379 | -1.862387 | -0.649366 |
| 8 | 6 | 0 | 1.114057 | -0.557593 | -0.276315 |
| 9 | 6 | 0 | 3.644815 | -0.621084 | 2.336403 |
| 10 | 6 | 0 | 4.291894 | 0.293896 | 1.264753 |
| 11 | 6 | 0 | 3.589814 | -0.135531 | -0.042302 |
| 12 | 6 | 0 | 4.112504 | -1.531235 | -0.448894 |
| 13 | 6 | 0 | 3.474556 | -2.456510 | 0.629382 |
| 14 | 6 | 0 | 2.669780 | -1.455085 | 1.492201 |
| 15 | 6 | 0 | 3.619833 | 0.934592 | -1.115411 |
| 16 | 6 | 0 | 2.245073 | 2.802479 | -1.627749 |
| 17 | 7 | 0 | 2.246774 | -0.466845 | 0.485361 |
| 18 | 8 | 0 | -4.167884 | -1.325292 | 0.556078 |
| 19 | 8 | 0 | 1.056503 | -0.164319 | -1.435449 |
| 20 | 8 | 0 | 2.895961 | 2.039613 | -0.633265 |
| 21 | 1 | 0 | -0.064759 | -0.863810 | 1.486629 |
| 22 | 1 | 0 | -1.252804 | -0.032624 | -1.089760 |
| 23 | 1 | 0 | -2.021672 | -0.081126 | 0.515740 |
| 24 | 1 | 0 | -1.291914 | -1.855531 | -2.556678 |
| 25 | 1 | 0 | -2.055832 | -3.418041 | -2.177680 |
| 26 | 1 | 0 | -0.073458 | -4.209428 | -1.212611 |
| 27 | 1 | 0 | 0.753019 | -2.697508 | -1.645339 |
| 28 | 1 | 0 | 0.349091 | -3.256760 | 1.010120 |
| 29 | 1 | 0 | -3.903503 | -1.219000 | -1.506284 |
| 30 | 1 | 0 | -4.093150 | -2.855364 | -0.829037 |
| 31 | 1 | 0 | 4.366103 | -1.253842 | 2.860040 |
| 32 | 1 | 0 | 4.075361 | 1.347217 | 1.454801 |
| 33 | 1 | 0 | 5.375568 | 0.167857 | 1.185329 |
| 34 | 1 | 0 | 5.205156 | -1.574927 | -0.459625 |
| 35 | 1 | 0 | 3.749251 | -1.786360 | -1.449811 |
| 36 | 1 | 0 | 2.800968 | -3.186361 | 0.168353 |
| 37 | 1 | 0 | 4.213548 | -3.005407 | 1.219717 |
| 38 | 1 | 0 | 1.840253 | -1.891059 | 2.048303 |
| 39 | 1 | 0 | 4.671279 | 1.207532 | -1.307252 |
| 40 | 1 | 0 | 3.181791 | 0.554182 | -2.042159 |

| | | | | | |
|---|---|---|---|---|---|
| 41 | 1 | 0 | 2.979350 | 3.390908 | -2.200805 |
| 42 | 1 | 0 | 3.100376 | -0.033925 | 3.079639 |
| 43 | 1 | 0 | 0.799367 | 4.380535 | -1.679086 |
| 44 | 1 | 0 | 1.721444 | 2.128296 | -2.320695 |
| 45 | 6 | 0 | 1.247777 | 3.713699 | -0.931192 |
| 46 | 1 | 0 | 1.789596 | 4.350185 | -0.219737 |
| 47 | 6 | 0 | 0.152655 | 2.934684 | -0.202803 |
| 48 | 1 | 0 | 0.624414 | 2.242129 | 0.506933 |
| 49 | 6 | 0 | -0.822677 | 3.849216 | 0.541473 |
| 50 | 1 | 0 | -0.379137 | 2.310113 | -0.933024 |
| 51 | 1 | 0 | -0.259573 | 4.427223 | 1.285548 |
| 52 | 1 | 0 | -1.247520 | 4.583195 | -0.158697 |
| 53 | 6 | 0 | -4.932120 | 0.901764 | -0.075451 |
| 54 | 6 | 0 | -4.039985 | 1.637597 | 0.934476 |
| 55 | 1 | 0 | -3.509651 | 0.890523 | 1.538065 |
| 56 | 1 | 0 | -4.655886 | 2.213186 | 1.637259 |
| 57 | 1 | 0 | -4.401476 | 0.816575 | -1.033475 |
| 58 | 1 | 0 | -5.845209 | 1.471289 | -0.285407 |
| 59 | 7 | 0 | -1.709397 | -2.910813 | 0.512607 |
| 60 | 1 | 0 | -2.012154 | -2.551421 | 1.420603 |
| 61 | 6 | 0 | -1.962815 | 3.096784 | 1.236389 |
| 62 | 1 | 0 | -2.454024 | 3.752638 | 1.966625 |
| 63 | 1 | 0 | -1.537674 | 2.259787 | 1.810296 |
| 64 | 6 | 0 | -3.011296 | 2.551617 | 0.267066 |
| 65 | 1 | 0 | -3.520089 | 3.387114 | -0.233646 |
| 66 | 1 | 0 | -2.508567 | 1.983200 | -0.525411 |
| 67 | 6 | 0 | -5.309619 | -0.501719 | 0.394958 |
| 68 | 1 | 0 | -5.786712 | -0.456098 | 1.379878 |
| 69 | 1 | 0 | -6.020206 | -0.971762 | -0.300770 |

## 18(C10)-*cis*

Energy at the level of M06-2X/6-311++G(d,p) (SCRF = SMD, methanol) // M06-2X/6-31G(d): -1312.951228

Number of imaginary frequency: 0

Correction for Gibbs free energy at 25 °C (M06-2X/6-311++G(d,p), SCRF = SMD, methanol): 0.599395

Standard orientation (M06-2X/6-31G(d))

| Center Number | Atomic Number | Atomic Type | Coordinates (Angstroms) X | Y | Z |
|---|---|---|---|---|---|
| 1 | 6 | 0 | -0.694474 | 2.522845 | -0.422205 |
| 2 | 6 | 0 | 0.337128 | 3.662948 | -0.550702 |
| 3 | 1 | 0 | 0.678007 | 4.002599 | 0.430014 |
| 4 | 1 | 0 | -0.090843 | 4.511161 | -1.094316 |
| 5 | 6 | 0 | 1.474602 | 2.987099 | -1.360569 |
| 6 | 1 | 0 | 1.542370 | 3.409611 | -2.365678 |
| 7 | 1 | 0 | 2.445339 | 3.067759 | -0.871211 |
| 8 | 6 | 0 | 0.960189 | 1.544520 | -1.463635 |
| 9 | 1 | 0 | 1.415327 | 0.968023 | -2.273181 |
| 10 | 6 | 0 | 0.999490 | 0.836670 | -0.063612 |
| 11 | 1 | 0 | 0.792528 | -0.227372 | -0.185014 |
| 12 | 6 | 0 | -0.192703 | 1.534050 | 0.654063 |
| 13 | 1 | 0 | -0.980656 | 0.811471 | 0.889067 |
| 14 | 1 | 0 | 0.119245 | 2.034435 | 1.573057 |
| 15 | 6 | 0 | -2.115758 | 3.000548 | -0.249641 |
| 16 | 1 | 0 | -2.193154 | 3.615287 | 0.662740 |
| 17 | 1 | 0 | -2.391894 | 3.629971 | -1.110332 |

| | | | | | |
|---|---|---|---|---|---|
| 18 | 6 | 0 | 2.290637 | 1.004563 | 0.721194 |
| 19 | 6 | 0 | 3.580698 | -1.113176 | -0.114861 |
| 20 | 6 | 0 | 4.785662 | -0.405987 | -0.789873 |
| 21 | 1 | 0 | 4.436036 | 0.211528 | -1.625597 |
| 22 | 1 | 0 | 5.505384 | -1.129219 | -1.184896 |
| 23 | 6 | 0 | 5.358260 | 0.470972 | 0.356421 |
| 24 | 1 | 0 | 6.407898 | 0.257762 | 0.575929 |
| 25 | 1 | 0 | 5.264045 | 1.534721 | 0.123092 |
| 26 | 6 | 0 | 4.427108 | 0.114164 | 1.531615 |
| 27 | 1 | 0 | 4.360966 | 0.870153 | 2.310078 |
| 28 | 6 | 0 | 4.724087 | -1.316916 | 2.006251 |
| 29 | 1 | 0 | 4.218806 | -1.519309 | 2.953210 |
| 30 | 1 | 0 | 5.795816 | -1.478774 | 2.149563 |
| 31 | 6 | 0 | 4.125934 | -2.175014 | 0.861540 |
| 32 | 1 | 0 | 4.861030 | -2.817570 | 0.367672 |
| 33 | 1 | 0 | 3.300531 | -2.798025 | 1.212218 |
| 34 | 6 | 0 | 2.601912 | -1.663604 | -1.123667 |
| 35 | 1 | 0 | 2.246577 | -0.862329 | -1.787129 |
| 36 | 1 | 0 | 3.147943 | -2.385187 | -1.754633 |
| 37 | 6 | 0 | 0.465547 | -2.594066 | -1.370940 |
| 38 | 1 | 0 | 0.746319 | -3.438016 | -2.021504 |
| 39 | 1 | 0 | 0.271919 | -1.726804 | -2.027310 |
| 40 | 6 | 0 | -0.783308 | -2.900419 | -0.566723 |
| 41 | 1 | 0 | -0.944367 | -2.061660 | 0.121397 |
| 42 | 1 | 0 | -0.618088 | -3.791861 | 0.050552 |
| 43 | 6 | 0 | -2.002771 | -3.084146 | -1.468306 |
| 44 | 1 | 0 | -2.105171 | -2.195248 | -2.107422 |
| 45 | 1 | 0 | -1.838478 | -3.933873 | -2.143318 |
| 46 | 1 | 0 | -6.453746 | -0.945443 | 1.909622 |
| 47 | 6 | 0 | -4.732506 | 0.322664 | 1.572356 |
| 48 | 1 | 0 | -3.640753 | 0.231387 | 1.587262 |
| 49 | 1 | 0 | -4.995499 | 0.978784 | 2.412189 |
| 50 | 6 | 0 | -5.142451 | 1.004418 | 0.266939 |
| 51 | 1 | 0 | -4.994758 | 0.330618 | -0.585473 |
| 52 | 1 | 0 | -6.209004 | 1.261477 | 0.289033 |
| 53 | 6 | 0 | -4.329693 | 2.261814 | 0.009566 |
| 54 | 1 | 0 | -4.688033 | 2.790567 | -0.887611 |
| 55 | 1 | 0 | -4.419721 | 2.956102 | 0.862262 |
| 56 | 7 | 0 | -0.471671 | 1.801731 | -1.693988 |
| 57 | 7 | 0 | 3.134578 | -0.074273 | 0.849791 |
| 58 | 8 | 0 | -2.978733 | 1.888956 | -0.159578 |
| 59 | 8 | 0 | 2.531334 | 2.052629 | 1.309321 |
| 60 | 8 | 0 | 1.518512 | -2.285287 | -0.482072 |
| 61 | 6 | 0 | -5.367816 | -1.053341 | 1.791294 |
| 62 | 1 | 0 | -4.992129 | -1.460255 | 2.738737 |
| 63 | 6 | 0 | -5.082002 | -2.061115 | 0.670964 |
| 64 | 1 | 0 | -5.425995 | -3.054395 | 0.987780 |
| 65 | 1 | 0 | -5.671024 | -1.810391 | -0.221245 |
| 66 | 6 | 0 | -3.606900 | -2.137350 | 0.278516 |
| 67 | 6 | 0 | -3.311861 | -3.277358 | -0.700196 |
| 68 | 1 | 0 | -2.995339 | -2.251542 | 1.184037 |
| 69 | 1 | 0 | -3.298318 | -1.183401 | -0.174825 |
| 70 | 1 | 0 | -3.292729 | -4.231882 | -0.157844 |
| 71 | 1 | 0 | -4.133550 | -3.351411 | -1.424373 |
| 72 | 1 | 0 | -1.006152 | 0.929267 | -1.673300 |

---------------------------------------------------------------------

## 18(C10)-TS

Energy at the level of M06-2X/6-311++G(d,p) (SCRF = SMD, methanol) // M06-2X/6-31G(d): -1312.931317

Number of imaginary frequency: 1

Correction for Gibbs free energy at 25 °C (M06-2X/6-311++G(d,p), SCRF = SMD, methanol): 0.598334

Standard orientation (M06-2X/6-31G(d))

| Center Number | Atomic Number | Atomic Type | Coordinates (Angstroms) X | Y | Z |
|---|---|---|---|---|---|
| 1 | 6 | 0 | -1.168609 | -2.550733 | -0.120922 |
| 2 | 6 | 0 | -0.205556 | -3.718893 | -0.401882 |
| 3 | 1 | 0 | 0.079766 | -3.761996 | -1.456202 |
| 4 | 1 | 0 | -0.669952 | -4.670669 | -0.125966 |
| 5 | 6 | 0 | 0.988117 | -3.386851 | 0.534907 |
| 6 | 1 | 0 | 1.115981 | -4.156519 | 1.299096 |
| 7 | 1 | 0 | 1.930960 | -3.283238 | -0.009466 |
| 8 | 6 | 0 | 0.521837 | -2.074274 | 1.189515 |
| 9 | 1 | 0 | 1.012984 | -1.829537 | 2.132679 |
| 10 | 6 | 0 | 0.578676 | -0.917421 | 0.135591 |
| 11 | 1 | 0 | 0.425858 | 0.025602 | 0.673681 |
| 12 | 6 | 0 | -0.597346 | -1.277530 | -0.794462 |
| 13 | 1 | 0 | -1.353390 | -0.487071 | -0.807247 |
| 14 | 1 | 0 | -0.261379 | -1.453866 | -1.819709 |
| 15 | 6 | 0 | -2.612234 | -2.854194 | -0.440353 |
| 16 | 1 | 0 | -2.702668 | -3.165066 | -1.494767 |
| 17 | 1 | 0 | -2.956812 | -3.686557 | 0.193184 |
| 18 | 6 | 0 | 1.921103 | -0.791927 | -0.534079 |
| 19 | 6 | 0 | 3.744195 | 0.844417 | -0.171815 |
| 20 | 6 | 0 | 4.609379 | 1.116401 | 1.073141 |
| 21 | 1 | 0 | 4.043064 | 1.720600 | 1.783121 |
| 22 | 1 | 0 | 5.529428 | 1.646822 | 0.810354 |
| 23 | 6 | 0 | 4.862612 | -0.316052 | 1.618112 |
| 24 | 1 | 0 | 5.922083 | -0.584682 | 1.650521 |
| 25 | 1 | 0 | 4.446107 | -0.432514 | 2.620934 |
| 26 | 6 | 0 | 4.090087 | -1.185455 | 0.609698 |
| 27 | 1 | 0 | 3.785378 | -2.161975 | 0.994081 |
| 28 | 6 | 0 | 4.872201 | -1.218405 | -0.722272 |
| 29 | 1 | 0 | 4.445795 | -1.952541 | -1.408685 |
| 30 | 1 | 0 | 5.925665 | -1.464968 | -0.560907 |
| 31 | 6 | 0 | 4.656688 | 0.219598 | -1.258660 |
| 32 | 1 | 0 | 5.588623 | 0.784843 | -1.356537 |
| 33 | 1 | 0 | 4.156077 | 0.202006 | -2.229226 |
| 34 | 6 | 0 | 2.917941 | 2.016992 | -0.656565 |
| 35 | 1 | 0 | 3.603013 | 2.783204 | -1.056642 |
| 36 | 1 | 0 | 2.265949 | 1.695076 | -1.485355 |
| 37 | 6 | 0 | 1.607498 | 3.813601 | 0.164140 |
| 38 | 1 | 0 | 2.341082 | 4.453097 | -0.351358 |
| 39 | 1 | 0 | 1.419603 | 4.249014 | 1.152002 |
| 40 | 6 | 0 | 0.295884 | 3.759237 | -0.613581 |
| 41 | 1 | 0 | 0.468260 | 3.413353 | -1.640933 |
| 42 | 1 | 0 | -0.085172 | 4.786553 | -0.692818 |
| 43 | 6 | 0 | -0.740288 | 2.872210 | 0.075836 |
| 44 | 1 | 0 | -0.459584 | 1.820206 | -0.055239 |
| 45 | 1 | 0 | -0.708334 | 3.061449 | 1.158509 |
| 46 | 1 | 0 | -5.633066 | 2.090574 | 1.793625 |
| 47 | 6 | 0 | -5.375166 | 0.183848 | 0.835937 |
| 48 | 1 | 0 | -6.119546 | -0.290531 | 1.487706 |
| 49 | 1 | 0 | -4.400452 | 0.003250 | 1.302032 |
| 50 | 6 | 0 | -5.399746 | -0.499311 | -0.532002 |
| 51 | 1 | 0 | -6.430700 | -0.582622 | -0.897267 |
| 52 | 1 | 0 | -4.846985 | 0.096826 | -1.267404 |
| 53 | 6 | 0 | -4.754793 | -1.873860 | -0.509449 |
| 54 | 1 | 0 | -5.226122 | -2.527395 | 0.242975 |
| 55 | 1 | 0 | -4.860541 | -2.363976 | -1.490015 |
| 56 | 7 | 0 | -0.917402 | -2.346622 | 1.323166 |
| 57 | 7 | 0 | 2.932128 | -0.289224 | 0.355913 |

| | | | | | |
|---|---|---|---|---|---|
| 58 | 8 | 0 | -3.385631 | -1.699462 | -0.210368 |
| 59 | 8 | 0 | 2.117375 | -1.072872 | -1.696883 |
| 60 | 8 | 0 | 2.170011 | 2.541597 | 0.414156 |
| 61 | 6 | 0 | -5.642016 | 1.690762 | 0.771544 |
| 62 | 1 | 0 | -6.651199 | 1.869493 | 0.377904 |
| 63 | 6 | 0 | -4.626175 | 2.474599 | -0.070959 |
| 64 | 1 | 0 | -4.766263 | 2.257601 | -1.137752 |
| 65 | 1 | 0 | -4.828520 | 3.547321 | 0.044371 |
| 66 | 6 | 0 | -3.173215 | 2.195309 | 0.312625 |
| 67 | 6 | 0 | -2.169272 | 3.085227 | -0.418679 |
| 68 | 1 | 0 | -2.931713 | 1.141566 | 0.112992 |
| 69 | 1 | 0 | -3.055444 | 2.336355 | 1.397614 |
| 70 | 1 | 0 | -2.224288 | 2.893202 | -1.499394 |
| 71 | 1 | 0 | -2.446281 | 4.139733 | -0.278593 |
| 72 | 1 | 0 | -1.415206 | -1.508951 | 1.633105 |

## 18(C10)-*trans*

Energy at the level of M06-2X/6-311++G(d,p) (SCRF = SMD, methanol) // M06-2X/6-31G(d): -1312.954919

Number of imaginary frequency: 0

Correction for Gibbs free energy at 25 °C (M06-2X/6-311++G(d,p), SCRF = SMD, methanol): 0.599586

Standard orientation (M06-2X/6-31G(d))

| Center Number | Atomic Number | Atomic Type | Coordinates (Angstroms) X | Y | Z |
|---|---|---|---|---|---|
| 1 | 6 | 0 | -1.833634 | -2.267321 | 0.117331 |
| 2 | 6 | 0 | -1.451415 | -2.471084 | -1.369059 |
| 3 | 1 | 0 | -1.570584 | -1.553642 | -1.949467 |
| 4 | 1 | 0 | -2.081544 | -3.244263 | -1.823232 |
| 5 | 6 | 0 | 0.029799 | -2.925993 | -1.261329 |
| 6 | 1 | 0 | 0.159938 | -3.953914 | -1.615848 |
| 7 | 1 | 0 | 0.708452 | -2.285442 | -1.829735 |
| 8 | 6 | 0 | 0.264460 | -2.872050 | 0.265937 |
| 9 | 1 | 0 | 1.091483 | -3.491402 | 0.623310 |
| 10 | 6 | 0 | 0.322496 | -1.395964 | 0.746223 |
| 11 | 1 | 0 | 0.616038 | -1.387384 | 1.798822 |
| 12 | 6 | 0 | -1.161816 | -0.969559 | 0.603978 |
| 13 | 1 | 0 | -1.570012 | -0.672921 | 1.575228 |
| 14 | 1 | 0 | -1.284386 | -0.146561 | -0.103602 |
| 15 | 6 | 0 | -3.306256 | -2.369443 | 0.438279 |
| 16 | 1 | 0 | -3.690009 | -3.375738 | 0.194813 |
| 17 | 1 | 0 | -3.448620 | -2.208655 | 1.518744 |
| 18 | 6 | 0 | 1.286259 | -0.537534 | -0.065347 |
| 19 | 6 | 0 | 3.749484 | 0.004154 | -0.352140 |
| 20 | 6 | 0 | 4.708311 | 0.284758 | 0.825537 |
| 21 | 1 | 0 | 4.491322 | 1.274075 | 1.233018 |
| 22 | 1 | 0 | 5.750518 | 0.263492 | 0.493869 |
| 23 | 6 | 0 | 4.364543 | -0.837774 | 1.838533 |
| 24 | 1 | 0 | 5.215956 | -1.478802 | 2.081732 |
| 25 | 1 | 0 | 3.971211 | -0.422186 | 2.769125 |
| 26 | 6 | 0 | 3.267303 | -1.613509 | 1.092766 |
| 27 | 1 | 0 | 2.612019 | -2.202662 | 1.732786 |
| 28 | 6 | 0 | 3.910005 | -2.394204 | -0.078202 |
| 29 | 1 | 0 | 3.193299 | -3.103281 | -0.504566 |
| 30 | 1 | 0 | 4.792025 | -2.956588 | 0.240506 |
| 31 | 6 | 0 | 4.237852 | -1.261256 | -1.092373 |

| | | | | | |
|---|---|---|---|---|---|
| 32 | 1 | 0 | 5.300464 | -1.204030 | -1.344566 |
| 33 | 1 | 0 | 3.672545 | -1.389226 | -2.021555 |
| 34 | 6 | 0 | 3.499178 | 1.209865 | -1.228900 |
| 35 | 1 | 0 | 4.469635 | 1.550455 | -1.632003 |
| 36 | 1 | 0 | 2.847831 | 0.939258 | -2.066694 |
| 37 | 6 | 0 | 2.337705 | 3.255353 | -1.193098 |
| 38 | 1 | 0 | 1.782843 | 2.836419 | -2.047350 |
| 39 | 1 | 0 | 3.131001 | 3.909166 | -1.590611 |
| 40 | 6 | 0 | 1.381584 | 4.023067 | -0.297264 |
| 41 | 1 | 0 | 0.990303 | 4.879220 | -0.862479 |
| 42 | 1 | 0 | 1.934369 | 4.429970 | 0.558847 |
| 43 | 6 | 0 | 0.229490 | 3.141047 | 0.180003 |
| 44 | 1 | 0 | -0.213523 | 2.650514 | -0.695412 |
| 45 | 1 | 0 | 0.632540 | 2.330260 | 0.798992 |
| 46 | 1 | 0 | -3.585324 | 0.809442 | 0.955866 |
| 47 | 6 | 0 | -4.752607 | 1.211093 | -0.808356 |
| 48 | 1 | 0 | -4.004566 | 0.817097 | -1.505422 |
| 49 | 1 | 0 | -5.224962 | 2.069526 | -1.302523 |
| 50 | 6 | 0 | -5.804802 | 0.122938 | -0.573687 |
| 51 | 1 | 0 | -6.667156 | 0.525200 | -0.027506 |
| 52 | 1 | 0 | -6.175378 | -0.231832 | -1.541746 |
| 53 | 6 | 0 | -5.250138 | -1.053534 | 0.219586 |
| 54 | 1 | 0 | -5.141702 | -0.780052 | 1.281488 |
| 55 | 1 | 0 | -5.931358 | -1.917791 | 0.173112 |
| 56 | 7 | 0 | -1.029752 | -3.270698 | 0.851522 |
| 57 | 7 | 0 | 2.577624 | -0.521201 | 0.385360 |
| 58 | 8 | 0 | -3.985366 | -1.389163 | -0.306285 |
| 59 | 8 | 0 | 0.908095 | 0.103736 | -1.037265 |
| 60 | 8 | 0 | 2.903198 | 2.205334 | -0.440646 |
| 61 | 6 | 0 | -4.025418 | 1.679508 | 0.451307 |
| 62 | 1 | 0 | -4.733124 | 2.134741 | 1.159097 |
| 63 | 6 | 0 | -2.900832 | 2.658258 | 0.121866 |
| 64 | 1 | 0 | -2.266642 | 2.195880 | -0.645215 |
| 65 | 1 | 0 | -3.318023 | 3.567979 | -0.333208 |
| 66 | 6 | 0 | -2.046289 | 3.042297 | 1.329492 |
| 67 | 6 | 0 | -0.837560 | 3.908372 | 0.959864 |
| 68 | 1 | 0 | -1.692583 | 2.126542 | 1.825281 |
| 69 | 1 | 0 | -2.673671 | 3.568344 | 2.060691 |
| 70 | 1 | 0 | -1.180519 | 4.768674 | 0.366661 |
| 71 | 1 | 0 | -0.386930 | 4.323628 | 1.870700 |
| 72 | 1 | 0 | -1.271902 | -4.208397 | 0.519777 |
